# A Proteome-Wide Immunoinformatics Tool to Accelerate T-Cell Epitope Discovery and Vaccine Design in the Context of Emerging Infectious Diseases: An Ethnicity-Oriented Approach

**DOI:** 10.3389/fimmu.2021.598778

**Published:** 2021-02-26

**Authors:** Patricio Oyarzun, Manju Kashyap, Victor Fica, Alexis Salas-Burgos, Faviel F. Gonzalez-Galarza, Antony McCabe, Andrew R. Jones, Derek Middleton, Bostjan Kobe

**Affiliations:** ^1^ Facultad de Ingeniería y Tecnología, Universidad San Sebastián, Sede Concepción, Concepción, Chile; ^2^ Departmento of Farmacología, Universidad de Concepción, Concepción, Chile; ^3^ Center for Biomedical Research, Faculty of Medicine, Autonomous University of Coahuila, Torreon, Mexico; ^4^ Institute of Systems, Molecular and Integrative Biology, University of Liverpool, Liverpool, United Kingdom; ^5^ School of Chemistry and Molecular Biosciences, Institute for Molecular Bioscience and Australian Infectious Diseases Research Centre, University of Queensland, Brisbane, QLD, Australia

**Keywords:** immunoinformatics, T-cell epitope, ethnicity, emerging-infectious disease, epitope discovery, vaccine design, SARS-CoV-2

## Abstract

Emerging infectious diseases (EIDs) caused by viruses are increasing in frequency, causing a high disease burden and mortality world-wide. The COVID-19 pandemic caused by the novel SARS-like coronavirus (SARS-CoV-2) underscores the need to innovate and accelerate the development of effective vaccination strategies against EIDs. Human leukocyte antigen (HLA) molecules play a central role in the immune system by determining the peptide repertoire displayed to the T-cell compartment. Genetic polymorphisms of the HLA system thus confer a strong variability in vaccine-induced immune responses and may complicate the selection of vaccine candidates, because the distribution and frequencies of HLA alleles are highly variable among different ethnic groups. Herein, we build on the emerging paradigm of rational epitope-based vaccine design, by describing an immunoinformatics tool (Predivac-3.0) for proteome-wide T-cell epitope discovery that accounts for ethnic-level variations in immune responsiveness. Predivac-3.0 implements both CD8+ and CD4+ T-cell epitope predictions based on HLA allele frequencies retrieved from the Allele Frequency Net Database. The tool was thoroughly assessed, proving comparable performances (AUC ~0.9) against four state-of-the-art pan-specific immunoinformatics methods capable of population-level analysis (NetMHCPan-4.0, Pickpocket, PSSMHCPan and SMM), as well as a strong accuracy on proteome-wide T-cell epitope predictions for HIV-specific immune responses in the Japanese population. The utility of the method was investigated for the COVID-19 pandemic, by performing *in silico* T-cell epitope mapping of the SARS-CoV-2 spike glycoprotein according to the ethnic context of the countries where the ChAdOx1 vaccine is currently initiating phase III clinical trials. Potentially immunodominant CD8+ and CD4+ T-cell epitopes and population coverages were predicted for each population (the Epitope Discovery mode), along with optimized sets of broadly recognized (promiscuous) T-cell epitopes maximizing coverage in the target populations (the Epitope Optimization mode). Population-specific epitope-rich regions (T-cell epitope clusters) were further predicted in protein antigens based on combined criteria of epitope density and population coverage. Overall, we conclude that Predivac-3.0 holds potential to contribute in the understanding of ethnic-level variations of vaccine-induced immune responsiveness and to guide the development of epitope-based next-generation vaccines against emerging pathogens, whose geographic distributions and populations in need of vaccinations are often well-defined for regional epidemics.

## Introduction

Emerging infectious diseases (EIDs) are defined as infections whose incidence or geographic range is rapidly increasing or threatens to increase in the near future. EIDs have emerged at an unprecedented rate due to a plethora of factors driven by globalization and climate change, posing serious threats to public health and economies (1). Wildlife is considered to be the major source of viral pathogens causing emerging zoonotic outbreaks (2), including mosquito-borne diseases (e.g., dengue, Zika fever) (3), rodent-borne hantaviruses (4) and bat-borne diseases (5), such as Ebola hemorrhagic fever, Nipah virus encephalitis and severe acute respiratory syndrome (SARS). According to the World Health Organization (WHO), disease outbreaks and epidemics caused by emerging pathogens are increasing in frequency over the past decades (6). In late 2019, the novel SARS-like CoV designated as 2019-nCoV (SARS-CoV-2) emerged in the city of Wuhan, China, causing a global pandemic with high morbidity and mortality (7). As of August 23^nd^ 2020, SARS-CoV-2 has caused ~23 million cases of the disease (COVID-19) and ~800,000 deaths across the world.

Vaccination is a critical tool in the response to unpredictable outbreaks of EIDs, but the complete process for bringing a vaccine from the research laboratory to the market is long, complex, and expensive (8). Traditional live-attenuated or whole-inactivated viral vaccines are slow to develop and have biosafety issues that make them poorly suited to respond to a rapidly evolving pandemic crisis, especially without the advantage of time and prior knowledge or experience with viral growth or pathogenesis mechanisms (1). Vaccine development for emerging pathogens is thus moving onto faster and more advanced recombinant and nucleic acid-based (DNA/RNA-based) approaches that address these issues by incorporating modern technologies and a rational design basis (9, 10). Accordingly, among the most advanced COVID-19 vaccine candidates are those encoding the SARS-CoV-2 spike (S) protein, which have proved to be safe and immunogenic over clinical development stages (11–13). These type of vaccines has recently initiated phase III clinical trials to evaluate protective efficacy at population level, including a recombinant adenovirus-vectored vaccine (ChAdOx1; NCT04400838) and a lipid nanoparticle-encapsulated mRNA-based vaccine (mRNA-1273; NCT04470427).

HLA class I and class II molecules play a central role in the immune response by presenting peptide antigens to CD8+ cytotoxic T-cells (CD8+ T-cell epitopes) and to CD4+ helper T-cells (CD4+ T-cell epitopes). However, the huge variability of the HLA system is a major issue for epitope-based vaccine design, since individuals display different sets of HLA alleles with variable ligand specificities (HLA-epitope restriction) and expression frequencies that substantially differ among ethnicities (14). Careful consideration of the HLA genetic background is thus paramount to ensure effectiveness and ethnically unbiased population coverage during vaccine development, especially considering variations in T-cell responses across multiple ethnicities (15). This problem is underscored by a significant body of evidence accounting for population-level associations of HLA polymorphisms with vaccine-induced immune responses (16) and also with vaccine failure (17, 18). Likewise, COVID-19 has been associated with disproportionate mortality amongst world populations (19, 20) and recent literature indicates that individuals from minority ethnic communities are at increased risk of infection from SARS-CoV-2 and subsequently adverse clinical outcome (21, 22). Individual genetic variations of the HLA system (different genotypes) may help explain differential T-cell mediated immune responses to the virus and could potentially alter the course of this disease (23), which has been well-described for the closely related SARS-CoV (24, 25).

Epitope-based vaccination is gaining interest in the scientific community, which allows for rational design of the immunogens based on short protein regions or peptides that avoid non-essential viral components and potentially toxic or immunosuppressive protein fragments (26, 27). These vaccines offer the prospect for a more prominent role of HLA-restricted T-cell immune responses (“T-cell vaccines”), by inducing large repertoires of T-cell specificities and further enabling rapid and economic large-scale production through recombinant DNA technology (28). Predicting the specificity of HLA class I-restricted CD8+ T-cell epitopes and HLA class II-restricted CD4+ T-cell epitopes is also a major consideration for epitope-based vaccine design, due to the influence of the HLA phenotype in the ability to mount effective immune responses (16). Therefore, immunoinformatics tools play a key role in this arena, as they allow to accelerate epitope discovery and vaccine design through *in silico* mapping of thousands of peptides (proteome-wide analysis) and by helping reduce the time and cost involved in experimental testing (21). In addition, these tools offer a framework to rationally deal with the enormous diversity HLA proteins, which reached 27,599 HLA alleles as of July 2020 (20,192 HLA class I and 7,407 HLA class II alleles), according to the IMGT/HLA Database (Release 3.41.0) (29).

A few immunoinformatics methods have been developed to aid the selection of T-cell epitopes by considering the fraction of individuals potentially covered by epitope-based vaccines (30, 31). Our previously reported method Predivac-2.0 optimizes the selection of HLA class II-restricted CD4+ T-cell epitopes predicted for specific target populations (32, 33). Herein, we extended our specificity-determining residues (SDRs) approach to CD8+ T-cell epitope prediction and subsequently describe a substantial enhancement of the method to build on the emerging paradigm of rational epitope-based vaccine design. The new Predivac-3.0 tool was successfully cross-validated and benchmarked against state-of-the-art pan-specific methods suited for population level analyses [NetMHCPan 4.0 (34), Pickpocket (35), PSSMHCPan (36) and SMM (37)], which are capable of using available experimental MHC binding data to infer binding preferences toward uncharacterized MHC molecules (38).

Predivac-3.0 was investigated for proteome-wide ethnicity-driven predictions to guide the discovery and selection of immunodominant HIV-1 specific T-cell epitopes, as well as the identification of epitope-dense regions (clusters) of CD8+ and CD4+ T-cell epitopes associated with high-population coverages (hotspots), in agreement with previous work showing the utility of *in silico* tools to identify epitope hotspots in the sequence of protein immunogens tested in subjects from different ethnic backgrounds (39, 40). We finally demonstrate the utility of the tool in the context of vaccine development for COVID-19 pandemic, by providing insight into putative T-cell epitopes and hotspots in the SARS-CoV-2 spike glycoprotein that are potentially immunodominant for the countries where the ChAdOx1 vaccine (University of Oxford/AstraZeneca) is currently carrying out phase III clinical trials (The United Kingdom, South Africa and Brazil).

To the best of our knowledge this is the first computational approach for ethnicity-driven proteome-wide discovery of T-cell epitopes and hotspots capable of inducing large repertoires of immune specificities in populations at risk of emerging pathogens, especially because the geographic distributions of the zoonotic viruses and populations in need of vaccinations are often well-defined for regional epidemics. Therefore, Predivac-3.0 holds potential to contribute in the understanding of vaccine-induced immune responsiveness in population contexts and to aid the rational design of epitope-based next-generation immunogens considering ethnic-level variations of vaccine induced immune responses for EIDs.

## Materials and Methods

### Semi-Automated Identification of SDR Positions

The identification of specificity-determining residues (SDRs) involved in peptide ligand-protein recognition events has been described previously for protein kinases (the Predikin tool) (41, 42) and for HLA class II proteins (the Predivac tool) (33), based on the inspection of crystal structures. Herein we introduce an improvement to the method for SDR determination, by implementing a Python-based semi-automated workflow to assist and simplify the identification of SDR positions in the peptide-HLA protein interface. We first constructed a dataset comprising 57 peptide-HLA class I complex structures (19 unique allotypes) available at the Protein Data Bank (PDB) (43) (**Table S1**). The structures were manually processed to select only the α chain with the corresponding bound peptide, focusing the analysis on the recognition region (groove) formed by the floor (eight antiparallel β-sheet folds) flanked by two polymorphic helical regions (α1 and α2 domains). The interaction interfaces were analyzed with the standalone version of the Arpeggio tool (44), which uses geometrical and biochemical features to automatically calculate and classify interatomic interactions between each pairs of atoms for a wide range of contact types (hydrogen bonds, halogen bonds, carbonyl interactions, hydrophobic interactions, among others). We assessed the role of each α-chain residues in contributing to peptide binding by considering all possible non-covalent pairwise interactions to extract nearest-neighbor atoms at each peptide position (p1 to p9), using Arpeggio´s default cut-off distance (5 Å). A consensus list of positions mainly involved in determining the interactions was built with a threshold of 30% occurrence, i.e. the residue in the interaction was present in 30% or more of the structures. Subsequently, analysis of conservation/variability and identification of polymorphic positions were carried out by means of two metrics: (i) Shannon entropy (45) and (ii) conservation score metrics described by Valdar in 2002 (46), which is implemented in the AACon tool^1^. AACon receives as input a list of aligned sequences in Clustal format, which was performed using MAFFT with default parameters (47) over a dataset of 10,089 HLA class I protein sequences (allotypes) from the Immuno Polymorphism Database (IPD)-IMGT/HLA Database release 3.37 (29). Finally, a small set of critical and polymorphic residue positions from the consensus list was selected as those dictating specific interactions in HLA class I proteins.

### Software Implementation

The new Predivac-3.0 method was re-written in Python 3.7 It consists of a main module that queries a purposed-built database of HLA class I and II specificity-determining residues (SDRs) that are associated with HLA protein sequences with high-affinity peptide binders (PredivacDB) and a database of HLA allele frequencies available at the Allele Frequency Net Database^2^ (AFND) (48). The PredivacDB was updated to include both experimentally validated high-affinity peptide ligands for HLA class I and class II proteins (**Table S2**). In total, the database contains 26,068 peptides, accounting for 77 HLA class I alleles (23,373 peptides) and 29 HLA class II alleles (2,695 peptides) that were exported and filtered from the Immune Epitope Database (IEDB) (49). HLA class I peptides with sequence length of 9 residues and experimentally determined binding affinity (K_D_/IC_50_/EC_50_) < 500 nM were considered, while sequences were removed if their binding affinity was determined by whole-cell based assays, non-natural atoms were present or Ala percentage > 50%. The method implemented by Predivac-3.0 requires from the user to provide the query proteome (Fasta file with multiple sequences) and to select the target population (country/region). The tool then fetches HLA allele data for this population (from the AFND) and automatically extracts SDRs information from the HLA query proteins to perform *in silico* T-cell epitope mapping. This is carried out by implementing the Predivac scoring scheme based on peptide ligands available in the PredivacDB. The whole procedure allows to predict ethnicity-driven CD8+ and CD4+ T-cell epitopes along with performing population coverage analysis, through a workflow that is subsequently explained.

### Scoring Scheme 

The Predivac binding score is calculated by establishing a predictive correlation between the SDRs in the HLA query protein(s) and the SDRs associated with HLA class I or class II proteins of known specificity from a pre-generated database (PredivacDB), by following the next steps: (i) SDRs are identified in the HLA protein sequence; (ii) PredivacDB is queried with the SDRs to retrieve peptide ligands associated with HLA proteins sharing similar residues in these positions (SDRs are considered similar if their sequence comparison using the BLOSUM62 substitution matrix returns a positive score); (iii) amino acid frequencies and weights are calculated from the binding data and (iv) *in silico* T-cell epitope mapping is carried out by parsing the protein sequence (query) into overlapping 9-mer segments (peptides), which are recursively assigned a binding score with the SDR-derived position weight matrix (sliding window technique). Predivac-3.0 selects by default T-cell epitopes scoring in the top 1% of the full set of peptides for a given protein, i.e. it employs a Peptide Percentile Rank (PPR) of binding scores of 1 (PPR = 1). However, the user is allowed to retrieve a greater number of putative T-cell epitopes by setting higher stringencies of 1, 2 or 3.

### Ethnicity-Driven T-Cell Epitope Mapping of Viral Proteomes

**Figure 1:**
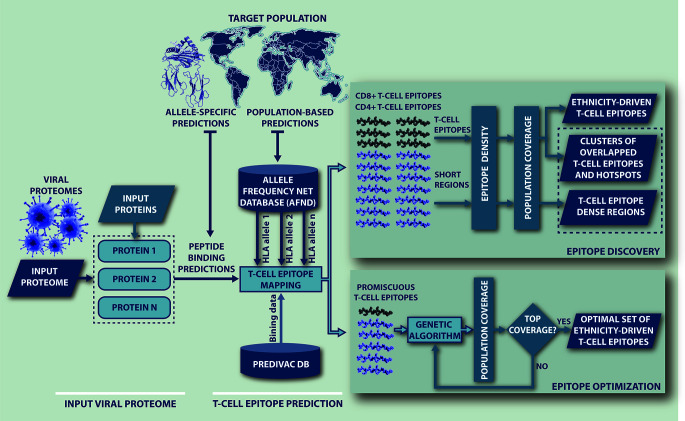
Flow-chart representing the steps followed by Predivac-3.0 to perform *in silico* ethnicity-driven T-cell epitope mapping over viral proteomes. The user must input the sequence of the query proteome (or individual proteins) and set the target population (country or geographic region), upon which the program retrieves from the AFND the HLA class I and class II allele frequency data available for population samples occurring in this country/region. Then, it searches the input proteome/proteins for putative CD8+ and CD4+ T-cell epitopes and epitope-rich regions (clusters/hotspots) by applying the SDR-based approach and querying the PredivacDB (Epitope Discovery mode). Predivac-3.0 also implements a genetic algorithm that explores and optimizes T-cell epitope combinations maximizing population coverage (Epitope Optimization mode).

A workflow of the algorithm is presented in **Figure 1**, showing that Predivac-3.0 accepts as input both protein sequences (Fasta file) and full proteome sequences (multi-Fasta file). The method runs for single HLA alleles (allele-specific T-cell epitope prediction) and for specific target populations (ethnicity-driven T-cell epitope prediction), by fetching HLA allele frequency distributions from ethnic populations available at the AFND. Ethnicity-driven T-cell epitope mapping follows a five-step process: (i) the user sets a target geographic region or country; (ii) the program retrieves from the AFND all available HLA class I and class II allele frequencies for population samples occurring in this country/region; (iii) the program applies the Predivac scoring scheme to predict T-cell epitopes for each HLA allele and then it searches for promiscuous epitopes restricted to as many alleles as possible in the target population; (iv) population coverage is calculated for each promiscuous T-cell epitope as the fraction of individuals that would be potentially covered in the selected target population, by implementing a previously reported algorithm (50); and (v) two alternative methods are implemented to select T-cell epitopes based on population coverage: (a) Epitope Discovery and (b) Epitope Optimization.

#### Epitope Discovery

This method outputs a full list of single putative T-cell epitopes with their corresponding positions in the query proteins sorted by population coverage, providing thus a top-down peptide ranking from the highest to minimal coverage calculated for the target population. In addition, the user is allowed to set a particular Population Coverage Threshold (PCT) to filter the report for T-cell epitopes delivering population coverage values higher than a given threshold (%). By default, the method retrieves all predicted T-cell epitopes (PCT = 0%).

#### Epitope Optimization

Predivac-3.0 implements a genetic algorithm (GA) that explores numerous combinations of putative epitopes to find a combination of *l* epitopes that maximizes the target population coverage. The pseudocode of the GA is shown below:

**Table:** 

Algorithm 1 Genetic Algorithm	
Input: Initial parameter for GA	
*PopSize* ← 100	
*MaxIteration* ← 50	
*Stopcicle* ← *False*	
*l* ← 1	
1:	*PopFitness* ← **Genetic.fitness**(*epitopeHits*)
2:	*MaxFitness*←**max**(*PopFitness*)
3:	*BestIndividual* ← **GetIndividual**(*PopFitness*)
4:	**while** *MaxFitness* ≤ **99 and not** stopcicle **do**
5:	*stopcond* ← *False*
6:	*i* ← 1
7:	*Population* ← **InitPopulation**(*epitopesHits,PopSize*)
8:	*Population* ← **PairwiseComb**(*Population,BestIndividual*)
9:	**while** *i < MaxIteration* **and not** *stopcond* **do**
10:	*PopFitness* ← **Genetic.fitness**(*Population*)
11:	*Parents* ← **Genetics.selection**(*Population,PopFitness,0.2*)
12:	*O f f spring* ← **Genetic.crossover**(*Parents,Population*)
13:	*Population* ← **Genetic.mutatePopulation**(*O f f spring,mutation Rate = 0.2*)
14:	*NewPopFitness* ← **Genetic.Fitness**(*Population*)
15:	*MaxFitness* ← **max**(*NewPopFitness*)
16:	*BestIndividual* ← **GetIndividual**(NewPopFitness)
17:	**if StopCondition**() **then**
18:	*stopcond* ← True
19:	**end if**
20:	*i* ← *i*+ 1
21:	**end while**
22:	*i* ← *i* + 1
23:	**If StopCicle**() **then**
24:	*stopcicle* ← *True*
25:	**end if**
16:	**end while**
27:	**return** the best solution

Predivac-3.0 seeds a first epitope (*l* = 1) to start the iterative process, which corresponds to the epitope (or individual) delivering the highest population coverage in the target population (*BestIndividual*). Each individual represents an epitope/HLA restricted alleles predicted by Predivac-3.0 (*EpitopesHits*). Then, a random population of 100 individuals is generated at each GA cycle (loop in lines 4-26) and subsequently paired with the previous *BestIndividual*. At each GA iteration (inner loop in lines 9-21), each individual is assigned a fitness score equal to the population coverage calculated for the target population (region/country). The top-20 individuals are selected to breed a new set of individuals by random, pairwise crossover, i.e. the top quintile of epitope combinations that retrieve the highest population coverage. Inner iterations are run until *MaxIteration* is achieved or until fitness score does not change during last 10 iterations. The GA runs until population coverage reaches a *MaxFitness* ≥ 99% or until the *MaxFitness* value does not change in two consecutive cycles by considering 3 significant figures (*StopCycle*), upon which the list of epitopes (best solution) is returned.

### Immunodominant T-Cell Epitope Clusters and Hotspots

For proteome-wide analysis, the program scans the protein sequences to detect regions with high-epitope density that are associated with a high population coverage in the target country/region. This process is performed through the Epitope Discovery mode of Predivac-3.0, by detecting clusters of epitope overlaps (in 9-mer regions) or by detecting epitope-rich regions in windows-frames of user-defined length (in 30-mer regions, by default). In 9-mer regions, epitope density was determined by considering both partially and completely overlapping epitopes, while in 30-mer regions only completely (full-length) overlapping epitopes were employed. Clusters meeting the following criteria were selected for proteome-wide analysis: (i) epitope density ≥ 90% of the top amount of T-cell epitope overlaps (in a proteome basis) and (ii) population coverage ≥ 20% in the target population. The statistical significance of these clusters was determined through a simulation procedure consisting of randomly selecting (1000 times) 10% of same-length regions, using the average epitope density of each simulation as the epitope distribution to calculate p-values. Those regions having p-value < 0.001 were considered as clusters. Further, overlapping clusters were merged together and potentially the most reactive (immunodominant) regions with population coverages ≥ 80% were denoted as hotspots.

### Validation of Allele-Specific Predictions

The predictive performance in identification of CD8+ T-cell epitopes was measured in terms of the area under the receiver operating characteristic curve (AUC), which is a graphical plot of the sensitivity versus the false positive rate (1 - specificity) as the discrimination threshold is varied. The AUC provides an indication of the accuracy of a prediction method, where an AUC = 1 corresponds to perfect predictions and AUC = 0.5 reflects random predictions. The method was assessed by leave-one-allele-out cross-validation (LOOCV) using a dataset of 17,425 high-affinity peptide binders restricted by 46 HLA class I alleles with 25 or more peptide ligands in PredivacDB, as previously reported (33). In addition, the method was benchmarked against the pan-specific methods NetMHCpan 4.0 (34), PickPocket (35), PSSMHCpan (36), and SMM (37). Two datasets were employed: (i) the IEDB-dataset, 5750 experimentally determined CD8+ T-cell epitopes (restricted by 47 HLA class I alleles) selected from the IEDB database and (ii) the DFRMLI-dataset, 887 high-affinity viral peptide ligands (tumor antigens were excluded) restricted by 7 HLA class I alleles (HLA-A*01:01, A*02:01, A*03:01, A*11:01, A*24:02, B*07:02, B*08:01, and B*15:01) (dataset available in **Supplementary Data Sheet 1**). The DFRMLI-dataset was built from high-throughput binding affinity data available at the Dana-Farber Repository for Machine Learning in Immunology^3^, which accounts for the cytomegalovirus (CMV) matrix protein pp65 (51), the human respiratory syncytial virus (RSV) and the human metapneumovirus (MPV) (52).

### Assessment of Population-Based Predictions

The ability of Predivac-3.0 to identify ethnicity-driven T-cell epitopes was tested on the HIV-1 proteome, by comparing CD8+ T-cell epitope predictions against a validation dataset derived from Los Alamos HIV Molecular Immunology Database^4^ (here referred as the HIV-dataset), which consists of 103 unique CD8+ T-cell epitopes that were experimentally determined from *in vivo/in vitro* studies carried out in Japan (53) (dataset available in **Supplementary Data Sheet 2**). This dataset includes immunodominant T-cell epitopes from the following HIV-1 proteins, using the reference strain HXB2 (GenBank K03455): Integrase (Pol; UniProt ID: P04585), envelope glycoprotein (gp160; UniProt ID: P04578), Gag polyprotein (Gag; UniProt ID: P04591), Nef protein (Nef; UniProt ID: P04601), viral protein R (Vpr; UniProt ID: P69726), and viral infectivity factor (Vif; UniProt ID: P69723). The predictive accuracy and efficiency were calculated by the following equations:

1 $$Accuracy\;(\%)=\left(\frac{number\;of\;correct\;matches}{total\;number\;of\;validation\;epitopes}\right)\times 100$$

2 $$Efficiency\;(\%)=(\frac{number\;of\;correct\;matches}{total\;number\;of\;predicted\;epitopes})\times 100$$

A correct match means that a predicted 9-mer T-cell epitope is equal to or it is contained in the sequence of an experimentally determined T-cell epitope from the validation dataset. Several analyses were further carried out regarding the capability of Predivac-3.0 to identify well-described immunodominant T-cell epitopes, including Japanese-specific protective epitopes from Gag and Pol protein regions included in the T-cell mosaic vaccine tHIVconsvX (**Table S3**) (44) and a number of T-cell epitopes recognized across multiple ethnicities (**Table S4**) (15).

### Proteome-Wide Visualization

Circular representations of the viral proteomes were generated to visualize ethnicity-driven T-cell epitope distributions, population coverage and immunodominant clusters (hotspots) across the viral proteins. Proteome maps were constructed using the Circos package (54), which renders concentric layers of information in the following data dimensions (from outside inward): (i) location of CD8+ and CD4+ T-cell epitopes relative to the reference strain HXB2 (epitope mapping); (ii) number of T-cell epitopes spanning each amino acid position (epitope density maps); (iii) percentage of individuals potentially covered by predicted T-cell epitopes in user-defined target populations (population coverage), both at each amino acid position (in nonameric clusters) and for windows frames of user-defined amino acid length (epitope-rich regions); and (iv) short epitope-rich regions associated with high-population coverages in the target population (hospots).

### SARS-CoV-2 Case Study

**Figure 2:**
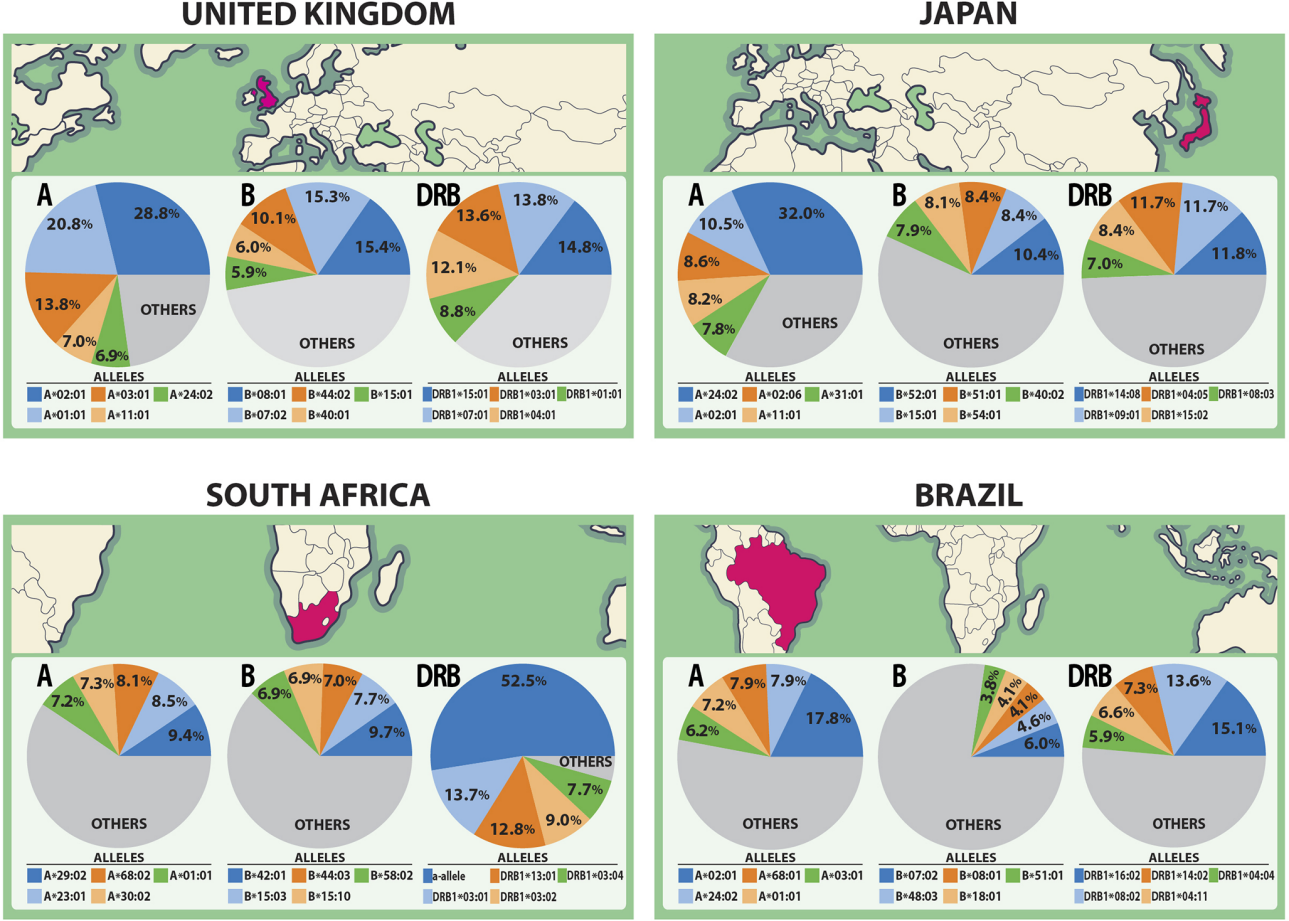
HLA class I and class II allele distributions in Japan and the countries where the ChAdOx1 vaccine (University of Oxford/AstraZeneca) is currently developing phase III clinical trials (The United Kingdom, South Africa and Brazil). The pie charts highlight the five most frequent HLA class I alleles (A and B loci) and class II alleles (DRB locus) in each population according to data from the AFND.

The spike glycoprotein of SARS-CoV-2 (UniProt ID: P0DTC2) was investigated with the Predivac-3.0 tools (using the Epitope Discovery and Epitope Optimization modes). The goal of this analysis was to identify immunodominant CD8+ and CD4+ T-cell epitopes and putative clusters/hotspots that are potentially specific or common to the populations of the three countries (The United Kingdom, South Africa and Brazil) where phase III clinical trials are currently underway for the ChAdOx1 adenovirus-vectored vaccine (University of Oxford/AstraZeneca). The Japanese population was additionally considered for comparison purposes to include an Asian ethnic background. HLA allele frequency distributions in the four target populations are illustrated in **Figure 2** (AFND data), including HLA class I (loci A and B) and HLA class II alleles (locus DRB).

## Results

### Allele-Specific T-Cell Epitope Predictions

Predivac-3.0 was implemented and assessed for its new capability of CD8+ T-cell epitope prediction, based on SDR positions that were determined in the peptide-HLA (pHLA) class I interaction interface through a combination of structural analysis of pHLA complex crystal structures and sequence analysis of HLA polymorphisms (see *Materials and Methods*). SDRs in the HLA protein sequence that were selected and implemented in the software are the following positions (for each P1-P9 peptide ligand position): P1 (62, 163), P2 (7, 9, 62, 99), P3 (66, 156, 159), P6 (70, 73, 156), P7 (152, 155, 156), P8 (76, 77) and P9 (77, 97, 116). Interactions for P4 and P5 were not considered, since the side-chains of amino acid residues in the middle of the peptides protrude out of the binding groove, delivering only marginal contributions to specificity.

**Figure 3:**
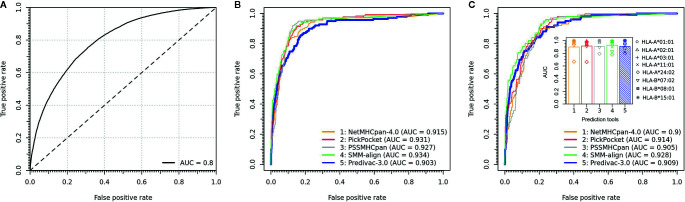
Validation of CD8+ T-cell epitope predictions. **(A)** Overall predictive performance of Predivac-3.0 measured by leave-one-out cross-validation over 46 HLA class I alleles having more than 25 associated peptide ligands in PredivacDB. The benchmark against pan-specific methods (NetMHCpan 4.0, Pickpocket, PSSMHCpan and SMM-align) using **(B)** a dataset of experimentally validated CD8+ T-cell epitopes derived from the IEDB Analysis Resource (IEDB-dataset) and **(C)** a dataset of high-affinity peptide ligands derived from the Dana-Farber Repository for Machine Learning in Immunology (DFRMLI-dataset) (See *Material and Methods*).

The LOOCV procedure to determine the accuracy on HLA class I alleles involves the exclusion of a single allele from the database and then assessing the performance using the binding data associated with that particular excluded allele. To build balanced datasets for AUC calculation, we followed an established validation strategy based on splitting the source protein of each epitope (positive) into overlapping peptides of the same length, and all peptides except the annotated peptide were taken as negatives. The predictive performance is shown in **Figure 3A**, proving a strong capability to predict high-affinity peptide ligands (overall AUC = 0.8). Predivac-3.0 was also benchmarked against state-of-the-art pan-specific methods for 8 HLA class I alleles using the DFRMLI-dataset (high-affinity peptide binders), yielding AUC values of 0.909 (Predivac-3.0), 0.900 (NetMHCpan-4.0), 0.914 (Pickpocket), 0.905 (PSSMHCpan) and 0.928 (SMM-align) (**Figure 3B**). Finally, performance comparison for CD8+ T-cell epitope predictions (IEDB-dataset) resulted in AUC values of 0.903 (Predivac-3.0), 0.915 (NetMHCpan4.0), 0.931 (PickPocket), 0.927 (PSSMHCpan) and 0.934 (SMM-align) (**Figure 3C**).

### Ethnicity-Driven T-Cell Epitope Prediction

**Figure 4:**
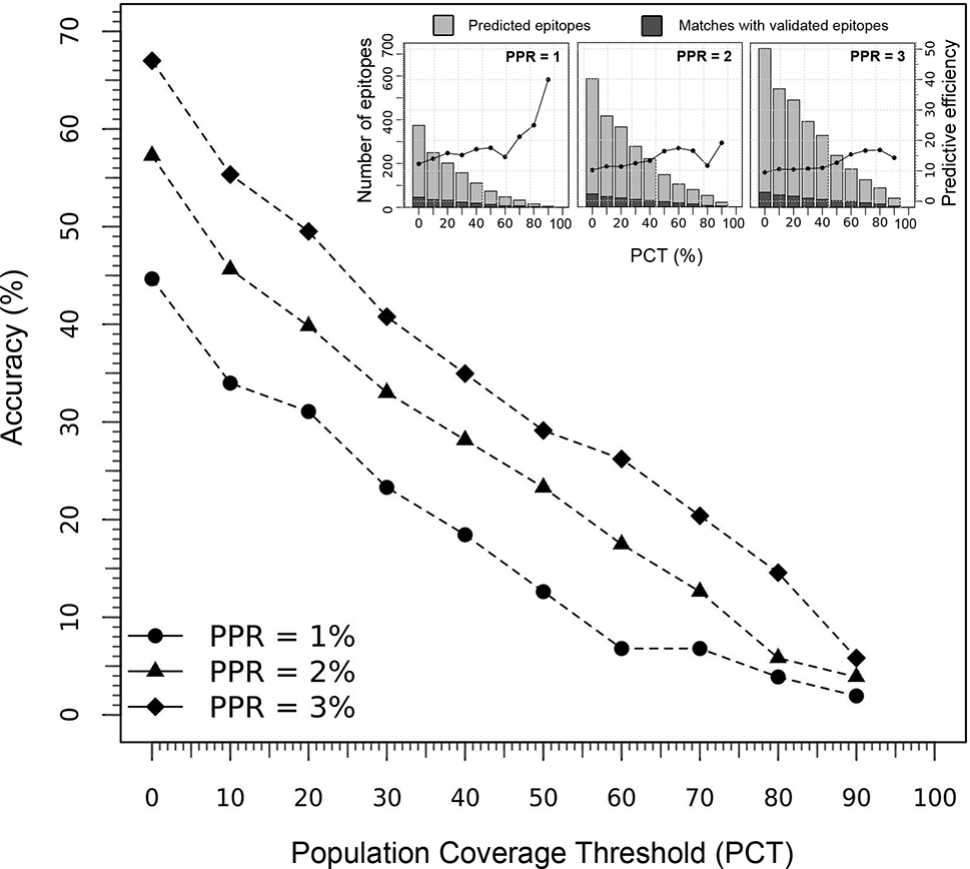
Predictive performance of Predivac-3.0 in identification of HIV-1 CD8+ T-cell epitopes specific for the Japanese population. The main figure illustrates the accuracy delivered by the tool at Peptide Percentile Rank (PPR) values of 1, 2 and 3, while the threshold of individuals potentially covered by the epitopes in this population was varied between 0-100% (PCT). The inset figures show the predictive efficiency of the tool at each PCT (for PPR 1, 2 and 3), while stacked bars represent the proportion of positive predictions or matches (dark grey) with respect to the total number of predictions (light grey).

The performance of the tool to deliver correct predictions of CD8+ T-cell epitopes and immunodominant hotspot was evaluated for the specific ethnic context of the Japanese population (using the Epitope Discovery and Epitope Optimized modes), by determining the accuracy and efficiency of the T-cell epitope mapping algorithm on the HIV-1 proteome (**Figure 4**). Using default parameters (PPR = 1; PCT = 0%), Predivac-3.0 predicted 374 putative CD8+ T-cell epitopes and detected 46 epitopes out of a top number of 103 T-cell epitopes in the HIV-dataset (accuracy = 44.7%; efficiency = 12.3%). The accuracy curves followed a comparable declination slope for the three PPR values (1, 2 and 3) and behaved similarly in the identification of CD8+ T-cell epitopes as the PCT was varied from 0 to 100%, with top accuracies (at PCT = 0%) of 44.7% (PPR = 1), 57.3% (PPR = 2) and 67% (PPR = 3). The search reduced up to 202 peptides by increasing the PCT to 20%, reaching a slightly lower accuracy of 31.1% (32 correct matches) with an increase in the predictive efficiency up to 15.8%. The predictive efficiency for PPR 1 continues to rise as the PCT increases, unlike for PPR 2 and PPR 3 that tend to maintain around average values. For PPR 1, the average efficiency (19.3%) proved statistically higher (p<0.05) than that for PPR 2 (13.9%) and PPR 3 (12.8%). For details, see **Table S5**.

### Proteome-Wide Analysis

**Figure 5:**
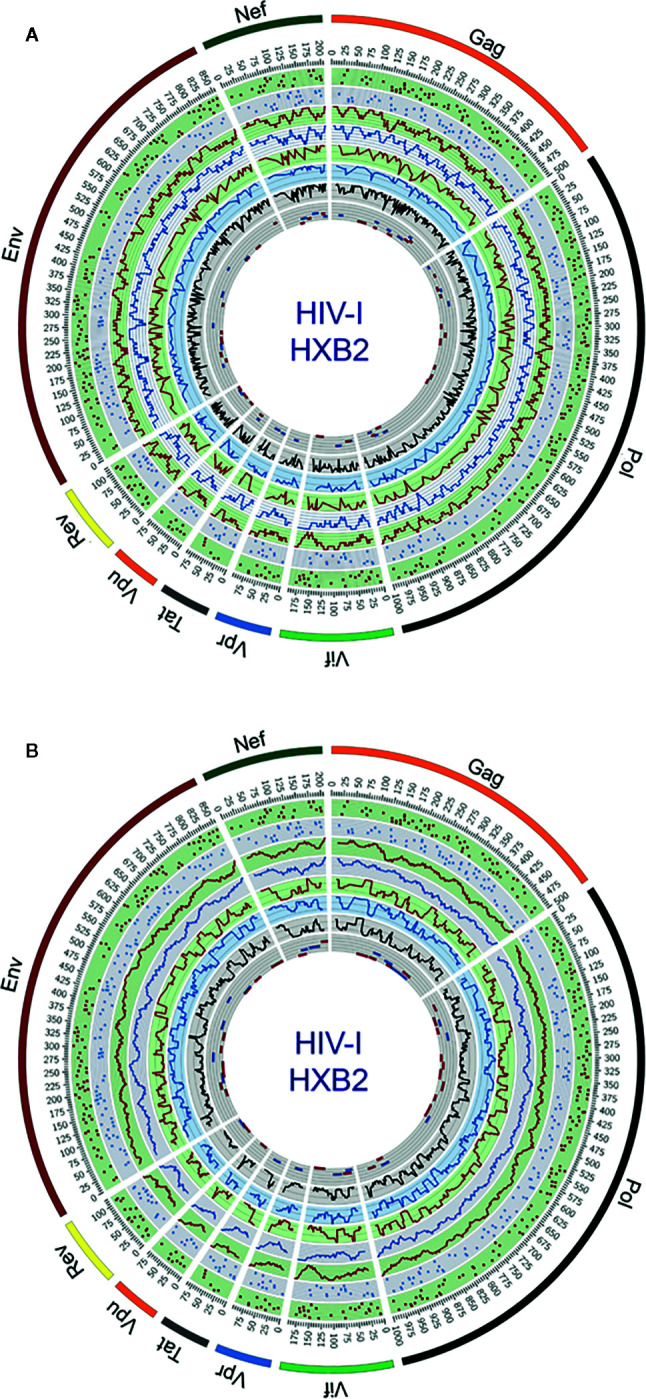
Circular plots showing the distribution of putative T-cell epitope clusters (hotspots) mapped onto the HIV-1 proteome and their corresponding population coverages predicted for the Japanese population, either for **(A)** nonameric windows frames of overlapped T-cell epitopes and **(B)** 30 residues long window frames of epitope-rich regions. Circles display the following features from the outside inward, based on the numbering standard of the reference strain HXB2 (GenBank K03455) for amino acid coordinates: ring 1, CD8+ T-cell epitope map (magenta dots), ring 2, CD4+ T-cell epitope map (blue dots); ring 3, CD8+ T-cell epitope density plot (magenta plot); ring 4, CD4+ T-cell epitope density map (blue plot); ring 5, population coverage calculated for CD8+ T-cell epitope clusters (magenta plot); ring 6, population coverage calculated for CD4+ T-cell epitope clusters (blue plot); ring 7, population coverage calculated for the combined set of CD8+ and CD4+ T-cell epitope clusters (black plot) and ring 8, putative hotpots for CD8+ T-cell epitope clusters (magenta lines) and CD4+ T-cell epitope clusters (blue lines). The innermost ring is divided by five parallel lines delimiting segments of population coverage ranges between 0-19%, 20-39%, 40-59%, 60-79% and 80-100%.

Circular maps for visualization of ethnicity-driven CD8+ and CD4+ T-cell epitopes are presented in **Figure 5** for the Japanese population, which provide information on the distribution of T-cell epitopes density across the HIV-1 proteome (rings 1–4), population coverage potentially afforded in the Japanese population (rings 5–7) and putative T-cell epitope clusters and hotspots (ring 8), both for nonameric T-cell epitope overlaps (**Figure 5A**) and for short regions (30 residues long) with high-concentration of putative T-cell epitopes (**Figure 5B**). As shown in proteome-wide plots, T-cell epitopes are concentrated in epitope-dense regions across the HIV-1 proteome, allowing the detection of relevant interactions between CD8+ and CD4+ T-cell epitope clusters located in Pol (111–141, 879–950), Env (619–646), Gag (267–301), Nef (68–109, 172–202), Vif (11–44), Vpu (26–49), and Rev (44–97). These regions are potential immunodominant hotspots delivering high population coverages in the Japanese population.

### Epitope Discovery and Epitope Optimization

**Figure 6:**
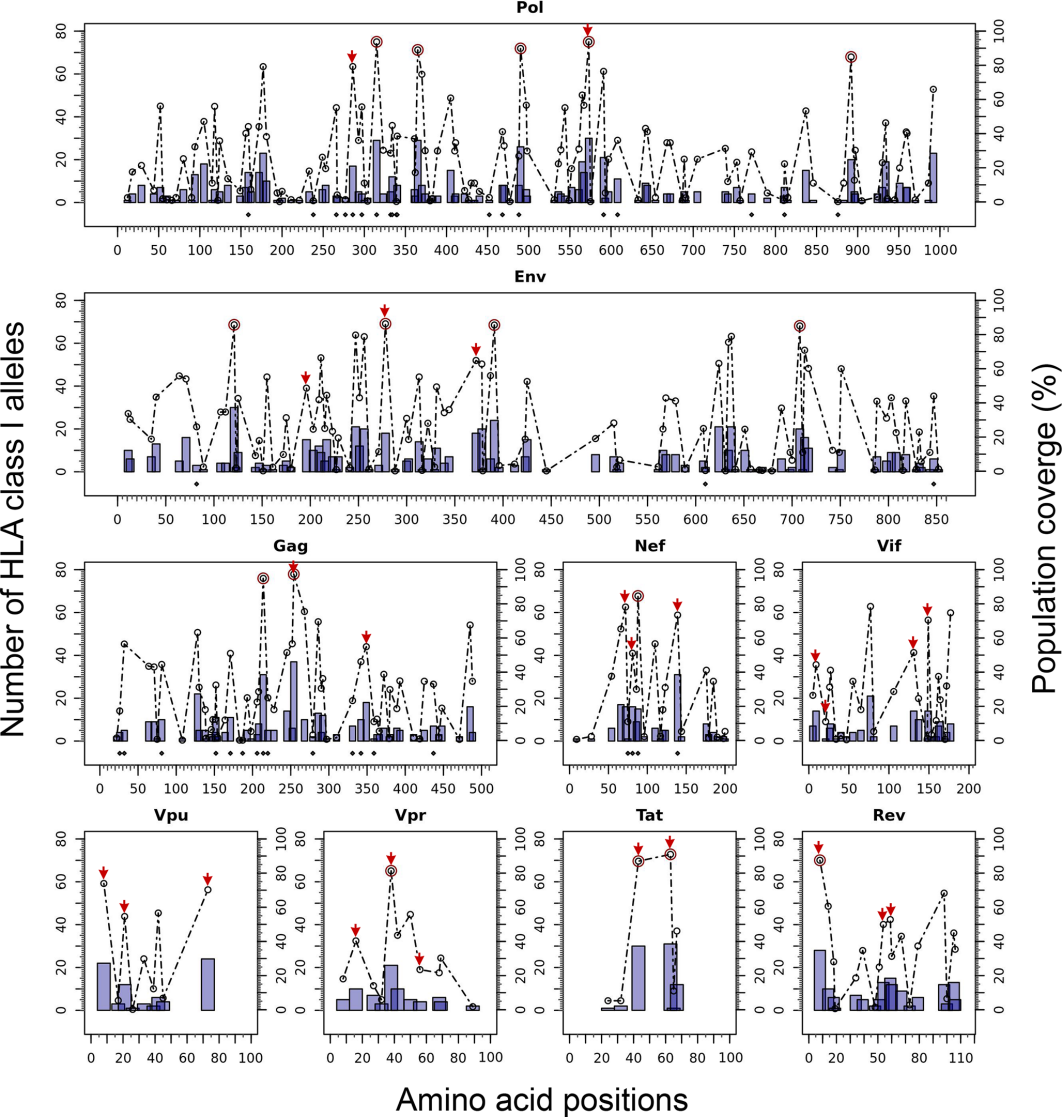
*In silico* HLA class I-restricted T-cell epitope mapping of the HIV-1 proteome (isolate HXB2) targeting the Japanese population, with the Epitope Discovery and Epitope Optimization modes of Predivac-3.0. Bars represent the position of putative CD8+ T-cell epitopes in Pol, Env, Gag, Nef, Vif, Vpu, Vpr, Tat and Rev proteins, whose height accounts for the number of HLA class I alleles restricted by each epitope and black diamonds denote matches with immunodominant CD8+ T-cell epitopes from the HIV-dataset. Circles correspond to the population coverage potentially afforded by each epitope, using red color to highlight specific epitopes delivering coverages ≥ 80% in the target population. Red arrows correspond to CD8+ T-cell epitopes predicted by Predivac-3.0 with the optimization algorithm.

The Predivac-3.0 output for the Epitope Discovery mode is presented in **Figure 6**, showing the position of all predicted CD8+ T-cell epitopes in the HIV-1 proteome (PPR = 1; PCT = 0), as well as the putative number of HLA class I alleles restricted by each epitope and the corresponding population coverages in the Japanese population. Promiscuous CD8+ T-cell epitopes predicted to cover ≥ 80% of this population are highlighted with red circles and listed in **Table 1** for the proteins Env (4 epitopes), Gag (2), Nef (1), Pol (5), Rev (1), Tat (2) and Vpr (1). The total number of CD8+ T-cell epitopes matching epitopes in the HIV-dataset (46 epitopes) is presented in **Table S6**, including Pol (21), Env (3), Gag (17) and Nef (5). Reactive T-cell epitopes predicted to cover ≥20% of the Japanese population are listed and described in **Tables S7** (CD8+ T-cell epitopes) and **Table S8** (CD4+ T-cell epitopes). The most reactive CD8+ T-cell epitopes (and population coverages) predicted in Gag are MTNNPPIPV (97%), AEWDRVHPV (94.9%), and ILDIRQGPK (69.5%); in Nef AVDLSHFLK (84.6%), FPVRPQVPL (78.1%), YPLTFGWCF (73.4%) and EEEEVGFPV (62.5%); and in Vpr FPRIWLHSL (81.3%). Likewise, a putative CD4+ T-cell epitope predicted inside a Gag cluster (RWIILGLNK) is predicted to cover 75.5% of the Japanese population. Interestingly, the top epitope predicted by Predivac-3.0 in the Nef protein (AVDLSHFLK) is also located within a peptide sequence (TYKA**AVDLSHFLK**EK) that was reported as the most frequently targeted (47%) from a cohort of HIV-1 infected US individuals (n=47) and also found within the predicted epitope cluster FPVTPQVPLRPMTYKA**AVDLSHFLK**EKGGLEGLIHSQRRQDI. Finally, **Table 2** describes the optimal set of promiscuous CD8+ T-cell epitopes predicted with the Epitope Optimization mode to maximize the population coverage in this country.

**Table 1:** Putative CD8+ T-cell epitopes from the HIV-1 proteome (HXB2 isolate) predicted by Predivac-3.0 to cover over 80% of the Japanese population (Epitope Discovery mode).

Protein	Peptide	Protein amino acid position	No of HLA class I alleles	Coverage (%)
Env	SVNFTDNAK	274–282	18	86.3
Env	KPCVKLTPL	117–125	30	85.7
Env	STQLFNSTW	387–395	24	85.6
Env	IVNRVRQGY	704–712	20	85.6
Gag	MTNNPPIPV	250–258	37	97.4
Gag	AEWDRVHPV	210–218	31	94.9
Nef	AVDLSHFLK	84–92	15	84.6
Pol	WEFVNTPPL	569–577	30	93.7
Pol	SPAIFQSSM	311–319	29	93.7
Pol	KQGQGQWTY	486–494	26	89.9
Pol	RQHLLRWGL	361–369	29	89.0
Pol	KTAVQMAVF	888–896	20	84.9
Rev	RSGDSDEEL	4–12	28	87.5
Tat	HQNSQTHQA	59–67	31	91.0
Tat	ITKALGISY	39–47	30	87.0
Vpr	FPRIWLHGL	34–42	21	81.3

**Table 2:** Optimal combinations of CD8+ T-cell epitopes predicted by Predivac-3.0 in each HIV-1 protein to maximize population coverage (≥ 99%) in the Japanese population (Epitope Optimization mode).

GA generation	T-cell epitope	Protein amino acid position	Individual population coverage	Combined population coverage
**Env**				
3	SVNFTDNAK	274–282	0.863	1.0
	KLTSCNTSV	192–200	0.486	
	DPEIVTHSF	368–376	0.648	
**Gag**				
2	MTNNPPIPV	250–258	0.974	0.996
	GPAATLEEM	338–346	0.462	
**Nef**				
3	FPVTPQVPL	68–76	0.781	1.0
	YPLTFGWCY	135–143	0.734	
	RPMTYKAAV	77–85	0.511	
**Pol**				
2	WEFVNTPPL	569–577	0.937	0.997
	YTAFTIPSI	282–290	0.793	
**Rev**				
3	RSGDSDEEL	4–12	0.875	1.0
	RQIHSISER	50–58	0.499	
	YLGRSAEPV	63–71	0.43	
**Tat**				
2	HQNSQTHQA	59–67	0.91	1.0
	ITKALGISY	39–47	0.87	
**Vif**				
**4**	WQVMIVWQV	5–13	0.444	1.0
	HIVSPRCEY	127–135	0.515	
	LQYLALAAL	145–153	0.705	
	RIRTWKSLV	17–25	0.11	
**Vpu**				
3	IPIVAIVAL	4–12	0.739	0.999
	VEMGHHAPW	69–77	0.702	
	IIAIVVWSI	17–25	0.546	
**Vpr**				
3	FPRIWLHGL	34–42	0.813	0.992
	REPHNEWTL	12–20	0.403	
	DTWAGVEAI	52–60	0.235	

### T-Cell Epitope Clusters and Immunodominant Hotspots

**Figure 7:**
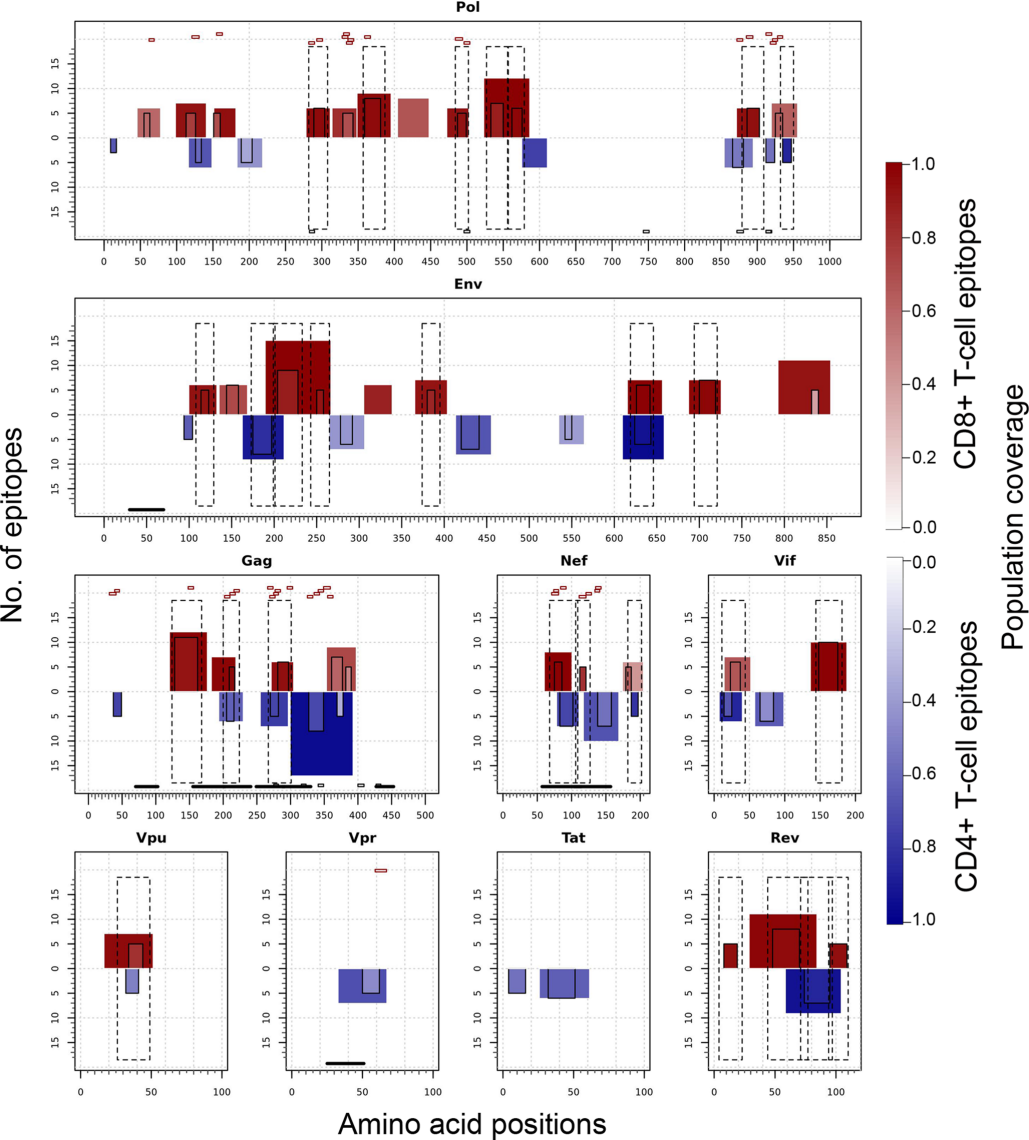
T-cell epitope clusters and hotspots predicted by Predivac-3.0 in the HIV-1 proteome, both for CD8+ T-cell epitopes (magenta bars) and CD4+ T-cell epitopes (blue bars). The left axis indicates the number of T-cell epitopes associated with each cluster and population coverage potentially afforded by the clusters in the Japanese population is represented according to a color scale in the range 0-100%. Potentially the most reactive (immunodominant) regions with population coverages ≥ 80% (hotspots) are highlighted inside dotted-line rectangles, while horizontal bars in the bottom denote ethnicity relevant sequences identified in a previous work (15) (black) and Japanese-specific HIV-1 vaccine-induced CD8+ T-cell epitopes (dashed). Small red rectangles on top of the clusters indicate the location of CD8+ T-cell epitopes from the HIV-dataset that colocalize with these regions.

**Figure 8:**
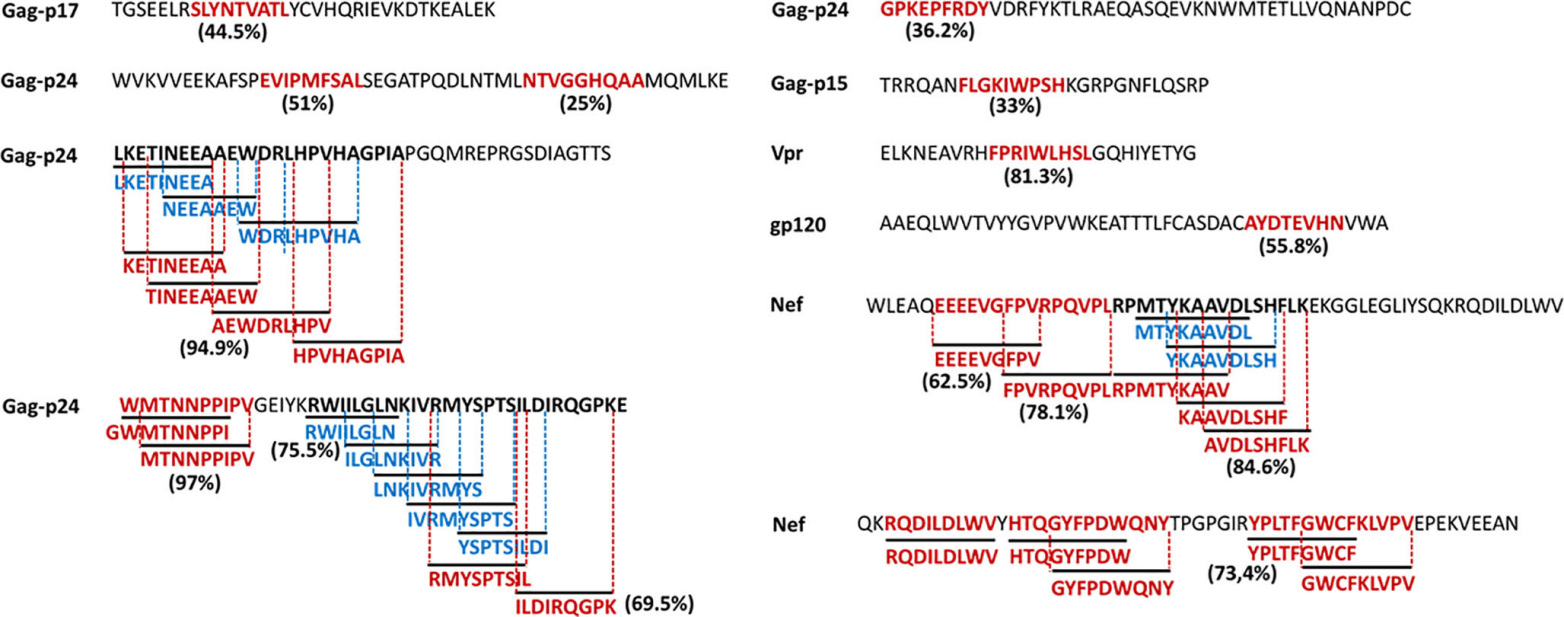
HIV-1 specific T-cell epitopes predicted by Predivac-3.0 for the Japanese population (Epitope Discovery mode), which match immunodominant regions identified across several ethnicities (15). Clusters of overlapped CD8+ (magenta) and CD4+ (blue) T-cell epitopes are highlighted for Gag and Nef, while population coverages associated with the most reactive T-cell epitopes are indicated next to the corresponding sequences.

**Figure 7** illustrates the position, number of associated T-cell epitopes and predicted population coverage (color scale 0-100%) of 66 HIV-1 specific T-cell epitope clusters spanning Env (14 epitopes), Gag (13), Nef (7), Pol (17), Rev (4), Tat (2), Vif (5), Vpr (1) and Vpu (3), both for CD8+ and CD4+ T-cell epitopes (magenta and blue bars, respectively). Detailed information about these T-cell epitope clusters is provided in **Supplementary Material** (**Table S9**). In addition, 48 epitope-rich regions are highlighted along with the nonameric T-cell epitope overlaps, according to information provided in the inner ring of Circos plots (see *Materials and Methods*). Potentially most reactive T-cell epitope clusters (hotspots) are highlighted inside dotted-line rectangles, which are regions predicted to deliver ≥ 80% of population coverage in Japan (**Table 3**). This figure also shows the colocalization of 42 T-cell epitopes from the HIV-dataset with several putative clusters (**Table S10**), which predictively would represent an accuracy of 40.8% and an efficiency of 63.6%. Detailed information on the position and statistical significance of CD8+ and CD4+ T-cell epitopes in each cluster predicted in the HIV-1 proteome are presented in **Tables S11** and **S12**. In addition, **Figure 7** highlights the position of 11 Japanese-specific vaccine-induced CD8+ T-cell epitopes (5 from Gag and 6 from Pol) that have proved protective in this population in response to the mosaic bivalent T-cell vaccine tHIVconsvX (55). While the four T-cell epitopes directly predicted by Predivac-3.0 were RMYSPTSIL, IYQEPFKNL, ELKKIIGQVR and TAFTIPSI, T-cell epitope clusters were capable of capturing knowledge on the position of 7 out of 11 epitopes. As shown for Pol, five epitopes were colocalized with putative clusters predicted in this protein: YTAFTIPSI (**YTAFTIPSI**NNETPGIRYQYNVLPQGW), IYQEPFKNL (IQKQGQGQWTYQ**IYQEPFK**), ELKKIIGQVR (VVESMNK**ELKKIIGQVR**DQA), GERIVDII and GERIVDIIA (YSA**GERIVDIIA**TDIQTKE) and two additional epitopes were found within Gag clusters: RMYSPTSI (ILGLNKIV**RMYSPTSI**LDIRQGPKEPFRDYVDRFY) and ATLEEMMTA (LLVQNANPDCKTILKALGPA**ATLEEMMTA**CQGVGG), providing insight into the validity of these broadly protective clusters and T-cell epitopes for the Japanese population. In addition, the position of several putative T-cell epitope clusters (in Gag and Nef proteins) overlapped with previously identified regions that were frequently recognized in HIV-tested subjects from four ethnicities (African-Americans, Caucasians, Hispanics, and West Indians) (15). **Figure 8** depicts the sequence and position of putative CD8+ and CD4+ T-cell epitope clusters overlapping these immunodominant regions, showing their colocalization with 4 protein regions (2 in Gag and 2 in Nef), as well as several putative CD8+ T-cell epitopes with high population coverages predicted for the Japanese population, such as AEWDRVHPV (Gag 210–218; 94.9%), MTNNPPIPV (Gag 250–258; 97%) and AVDLSHFLK (Nef 84–92; 84.6%).

**Table 3:** Immunodominant T-cell epitope clusters (hotspots) predicted by Predivac-3.0 in the HIV-1 proteome (HXB2 isolate) that are associated with population coverages ≥ 80% in the Japanese population.

Prot	Cluster		T-cell epitopes		Population coverage (%)
	Sequence	Position (start-end)	CD8+ T-cell epitopes	CD4+ T-cell epitopes	
Env	IISLWD**QSLKPCVKLT**PLCVSL	108–129 (114–123)	4	–	92.6
Env	YA**FFYKLDIIPIDNDTTSYKLTSCN**TSVI	173–201 (175–197)	–	7	81.9
Env	SVITQ**ACPKVSFEPIPIHYCAPAGFAILKC**NNKTF	199–233 (204–228)	8	–	88.5
Env	STVQCTH**GIRPVVSTQ**LLLNGSL	243–265 (250–258)	4	–	92.0
Env	HSFNCG**GEFFYCNSTQ**LFNSTW	374–395 (380–389)	4	–	85.7
Env	LEQIW**NHTTWMEWDREINNYTSLIH**SLI	619–646 (624–643)	5	5	99.9
Env	GLRIVF**AVLSIVNRVRQGYSP** **LSFQT**HL	694–721 (700–719)	6	–	97.1
Gag	HSNQ**VSQNYPIVQNIQGQMVHQAISPRTLNAWVKVVEEK**AFSPEV	124–168 (128–162)	10	–	92.0
Gag	MLKET**INEEAAEWDRVHP**VHAGPIA	200–224 (205–217)	4	5	99.0
Gag	ILG**LNKIVRMYSPTSILDIRQGPKEPFRDYV**DRFY	267–301 (270–297)	5	4	98.4
Nef	FPVTPQV**PLRPMTYKAAVDLSHFLK** **EKGGLEGLIH**SQRRQDI	68–109 (75–102)	5	6	99.9
Nef	RQDILD**LWIYHTQGYF**PDWQNY	106–127 (112–121)	4	–	81.4
Nef	EWRFD**SRLAFHHVARE**LHPEY	182–202 (187–197)	–	4	80.5
Pol	YTAFTIP**SINNETPGIRYQYNVL**PQGW	282–308 (289–304)	5	–	95.6
Pol	IE**ELRQHLLRWGLTTPD** **KKHQKEPP**FLWMGY	357–387 (359–381)	7	–	96.1
Pol	IQK**QGQGQWTYQIYQE**PFK	484–502 (487–499)	4	–	931
Pol	VQKITT**ESIVIWGKTPKFKLPIQK**ETWETW	527–556 (533–550)	6	–	88.1
Pol	WTEYW**QATWIPEWEFVNTPP**LVK	557–579 (562–576)	5	–	97.1
Pol	QVRDQAE**HLKTAVQMAVFIHNFKRK**GGIGGY	879–909 (886–903)	5	–	94.2
Pol	ITK**IQNFRVYYRDSRN**PLW	932–950 (935–947)	–	4	85.7
Rev	RSGD**SDEELIRTVRLI**KLLY	4–23 (8–19)	4	–	94.0
Rev	RWRE**RQRQIHSISERILGTYLGRSAEP**VPLQLPP	44–77 (48–70)	7	–	95.2
Rev	VPL**QLPPLERLTLDCNEDCGTSGTQ**GV	71–97 (74–95)	–	6	84.6
Rev	T**QGVGSPQILVESPTV**L	94–110 (95–109)	4	–	93.9
Vif	VWQV**DRMRIRTWKSLVKHHMYVSGKARG**WFYRHHY	11–44 (14–37)	5	4	88.5
Vif	SLQY**LALAALITPKKIKPPLPSVTKLTEDRWN**KPQKTK	144–181 (148–175)	9	–	99.5
Vpu	VIIEYR**KILRQRKIDRLID**RLIER	26–49 (32–44)	4	4	90.7

In each cluster sequence the core region of overlapped T-cell epitopes is highlighted in bold and underscored, providing further information on the amino acid position in each protein, the number of putative T-cell epitopes and the predicted population coverage in the target population.

### Application to SARS-CoV-2

**Figure 9:**
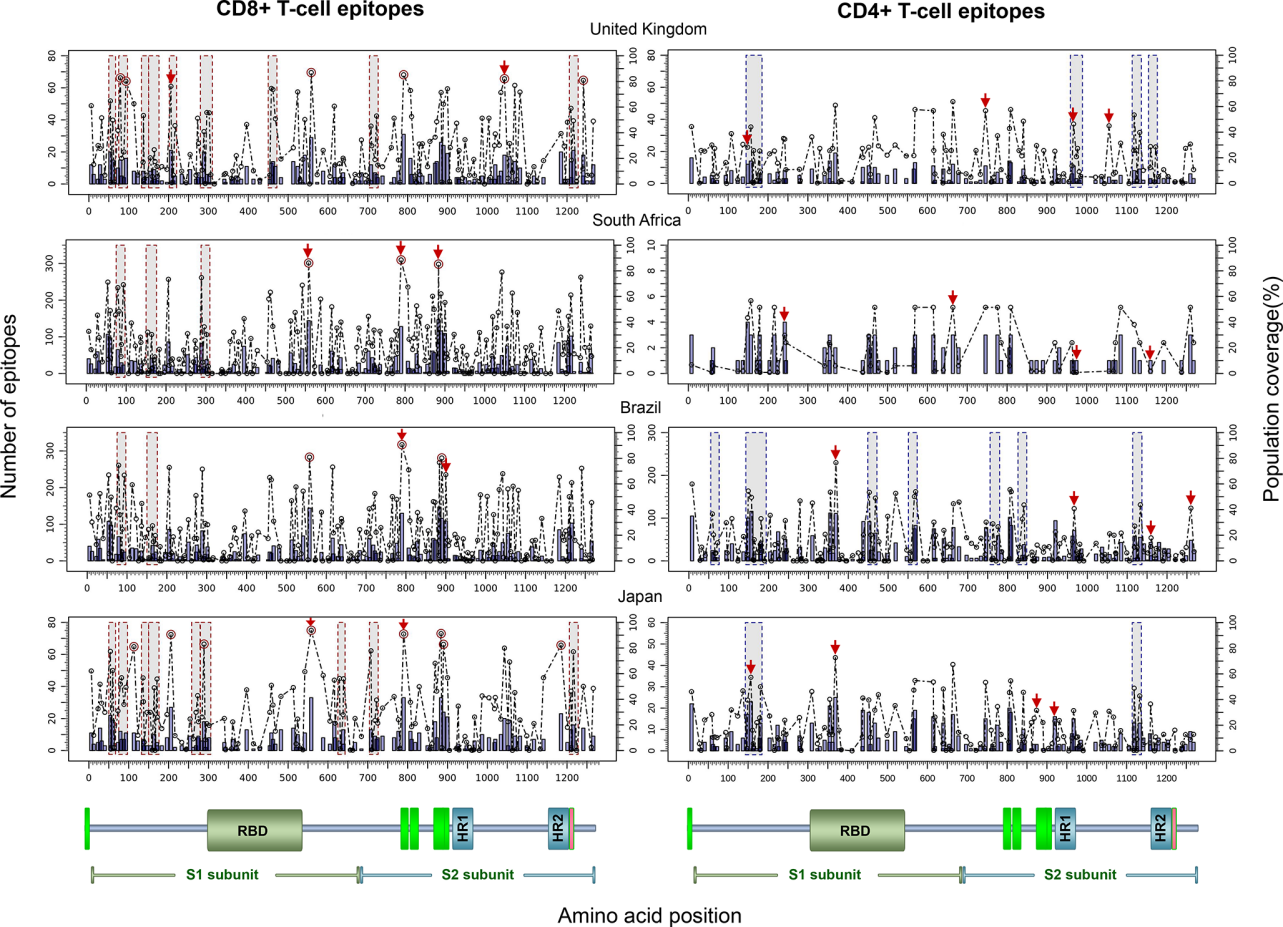
*In silico* T-cell epitope mapping of the SARS-CoV-2 spike glycoprotein (UniProt ID: P0DTC2) targeting the populations of The United Kingdom, South Africa, Brazil and Japan, using the Epitope Discovery and Epitope Optimization modes of Predivac-3.0. Bars in the plots represent the position of putative T-cell epitopes, whose height accounts for the number of CD8+ and CD4+ T-cell epitopes. Circles correspond to the population coverage potentially afforded by the epitopes in the target populations, with epitopes delivering coverages ≥ 60% indicated with red color. Shaded regions mark the position of putative T-cell epitope clusters delivering population coverages ≥ 60% (hotspots). In addition, T-cell epitopes predicted by Predivac-3.0 using the optimization algorithm are indicated over the circles with red arrows. A protein scheme with domain organization is presented on the bottom the figure, highlighting the position of the receptor-binding domain (RBD) and heptad repeat 1 and 2 (HR1 and HR2). Aromatic amino-acid rich pre transmembrane regions (PTM) involved in the mechanism of viral entry are represented in green color.

Finally, to test the utility of the method we performed *in silico* mapping on the spike glycoprotein of SARS-CoV-2. **Figure 9** presents the output for the Epitope Discovery mode by targeting the United Kingdom, South Africa, Brazil and Japan, showing the position of CD8+ and CD4+ T-cell epitopes and clusters potentially immunodominant for these populations, as well as the number of restricting HLAs and predicted population coverages. Most reactive T-cell epitopes in each country (population coverage ≥ 80%) are highlighted in red circles and listed in **Table 4**, showing the presence of two CD8+ T-cell epitopes that would afford high coverages for all the populations (ESNKKFLPF and KQIYKTPPI) and the epitope GTITSGWTF would be highly promiscuous for the South African, Brazilian and Japanese populations. **Figure 9** also highlights with red arrows the set of T-cell epitopes that was selected through the genetic algorithm (the Epitope Optimization mode) in order to maximize the coverage in each target population (**Table 5**). Top coverages (≥ 99%) could potentially be reached with 2 to 4 T-cell epitopes, with the exception of CD4+ T-cell epitopes for the South African population (72.4% coverage). **Table S13** shows with more detail the combination of epitopes selected by the algorithm at each generation. Finally, **Table 6** summarizes T-cell epitope clusters potentially delivering population coverages ≥ 80% in each target region (hotspots), providing a comprehensive description in **Supplementary Material** for putative clusters specific for the populations of the United Kingdom (**Table S14**), South Africa (**Table S15**), Brazil (**Table S16**) and Japan (**Table S17**). One particular region that rises interest spans positions 150 to 185 (KSWMESEFRVYSSANNCTFEYVSQPFLMDLEGKQ), which comprises both CD8+ and CD4+ T-cell epitope clusters consistently predicted for the populations of the United Kingdom, South Africa, Brazil and Japan.

**Table 4:** List of the top promiscuous CD8+ T-cell epitopes in the SARS-CoV-2 spike glycoprotein predicted by Predivac-3.0 to deliver above 80% coverage in the populations of the United Kingdom, South Africa, Brazil, and Japan (using the Epitope Discovery mode).

Protein	Peptide	Amino acid position	Coverage (%)	No. of HLA class I alleles
**The United Kingdom**				
**Spike**	**ESNKKFLPF**	**554–562**	**86.9**	**29**
**Spike**	**KQIYKTPPI**	**786–794**	**85.2**	**31**
Spike	GTKRFDNPV	75–83	82.8	15
**Spike**	**RVDFCGKGY**	**1039–1047**	**82.1**	**18**
Spike	MTSCCSCLK	1237–1245	80.9	18
Spike	GVYFASTEK	89–97	80.2	16
**South Africa**				
**Spike**	**KQIYKTPPI**	**786–794**	**88.5**	**128**
**Spike**	**ESNKKFLPF**	**554–562**	**86.1**	**144**
**Spike**	**GTITSGWTF**	**880–888**	**85.2**	**147**
**Brazil**				
**Spike**	**KQIYKTPPI**	**786–794**	**90.6**	**130**
**Spike**	**ESNKKFLPF**	**554–562**	**80.9**	**144**
Spike	WTFGAGAAL	886–894	80.2	116
**Japan**				
**Spike**	**ESNKKFLPF**	**554–562**	**93.8**	**33**
**Spike**	**GTITSGWTF**	**880–888**	**91.2**	**33**
**Spike**	**KQIYKTPPI**	**786–794**	**90.9**	**33**
Spike	KIYSKHTPI	202–210	90.5	27
Spike	ITDAVDCAL	285–293	83.0	18
Spike	WTFGAGAAL	886–894	82.9	24
Spike	KEIDRLNEV	1181–1189	82.2	23
Spike	TLDSKTQSL	109–117	81.0	11

In bold, epitope sequences that are potentially immunodominant in most of the countries.

**Table 5:** Optimal combinations of CD8+ and CD4+ T-cell epitopes selected by Predivac-3.0 in the SARS-CoV-2 spike glycoprotein at the last generation of the genetic (GA) algorithm, which maximize the population coverage in the United Kingdom, South Africa, Brazil, and Japan (Epitope Optimization mode).

T-cell epitopes	Amino acid position	Individual population coverage	Combined population coverage (%)	Putative allele restriction
**The United Kingdom**				
**CD8+ T-cell epitopes**				
KIYSKHTPI	202–210	0.762	99.2	A*02:01(0.288),B*07:02(0.152),B*51:01(0.046),A*01:01(0.207),A*03:01(0.137),A*11:01(0.069),A*24:02(0.068),A*25:01(0.021),A*26:01(0.019),B*57:01(0.036)
RVDFCGKGY	1039–1047	0.821		
**CD4+ T-cell epitopes**				
YHKNNKSWM	145–153	0.283	99.9	DRB1*11:01(0.045),DRB1*13:01(0.043),DRB1*13:02(0.032),DRB1*07:01(0.137),DRB1*01:01(0.088),DRB1*15:01(0.147),DRB1*04:04(0.040),DRB1*04:03(0.024),DRB1*03:01(0.135),DRB1*04:01(0.120),DRB1*04:04(0.040)
FPQSAPHGV	1052–1060	0.449		
VKQLSSNFG	963–971	0.464		
ICGDSTECS	742–750	0.567		
**South Africa**				
**CD8+ T-cell epitopes**				
KQIYKTPPI	786–794	0.885	100%	A*23:01(0.085),A*68:02(0.081),A*30:02(0.072),A*30:01(0.056),B*42:01(0.097),B*58:02(0.068),B*08:01(0.059),B*40:06(0.039),B*57:03(0.032),A*23:01(0.085),A*30:02(0.072),A*24:02(0.051),B*42:01(0.097),B*15:03(0.076),B*44:03(0.070),B*08:01(0.059),B*15:10(0.068),B*07:02(0.036),B*14:01(0.032).
ESNKKFLPF	554–562	0.861		
MIAQYTSAL	869–877	0.499		
**CD4+ T-cell epitopes**				
FQTLLALHR	238–246	0.298	72.4	DRB1*13:01(0.128),DRB1*13:02(0.03),DRB1*10:01(0.004),DRB1*13:05(0),DRB1*03:01(0.137),DRB1*03:02(0.09),DRB1*03:04(0.077),DRB1*09:01(0.008), DRB1*10:01(0.004)
YECDIPIGA	660–668	0.516		
FKNHTSPDV	1156–1164	0.0178		
FGAISSVLN	970–978	0.008		
**Brazil**				
**CD8+ T-cell epitopes**				
KQIYKTPPI	786–794	0.906	99.2	A*02:01(0.178),A*23:01(0.037),B*48:03(0.045),B*08:01(0.041),B*18:01(0.040),B*51:01(0.038),B*35:06(0.035),B*35:03(0.033),A*68:01(0.079),A*03:01(0.062),B*18:01(0.040),B*35:01(0.033),B*44:03(0.031),
IPFAMQMAY	896–904	0.672		
**CD4+ T-cell epitopes**				
YSVLYNSAS	365–373	0.766	99.2	DRB1*16:02(0.151),DRB1*08:02(0.136),DRB1*14:02(0.073),DRB1*11:01(0.032),DRB1*04:11(0.066),DRB1*04:04(0.058),DRB1*07:01(0.054),DRB1*09:01(0.034),DRB1*16:02(0.151),DRB1*03:01(0.057)
VKQLSSNFG	963–971	0.408		
FKNHTSPDV	1156–1164	0.179		
FDEDDSEPV	1256–1264	0.411		
**Japan**				
**CD8+ T-cell epitopes**				
ESNKKFLPF	554–562	0.938	99.9	A*24:02(0.319),A*26:01(0.072),A*26:02(0.018),B*52:01(0.104),B*15:01(0.083),B*51:01(0.083),B*35:01(0.078),B*44:03(0.070),A*02:01(0.105),A*02:06(0.085),B*52:01(0.104),B*51:01(0.083),B*54:01(0.081),B*40:02(0.078),B*40:01(0.051),B*40:06(0.047)
KQIYKTPPI	786–794	0.909		
**CD4+ T-cell epitopes**				
YSVLYNSAS	365–373	0.727	99.4	DRB1*04:05(0.117),DRB1*15:02(0.084),DRB1*08:03(0.069),DRB1*13:02(0.052),DRB1*01:01(0.048),DRB1*08:02(0.039),DRB1*14:08(0.118),DRB1*08:03(0.069),DRB1*08:02(0.039),DRB1*14:01(0.032),DRB1*09:01(0.117),DRB1*15:01(0.055)
MESEFRVYS	153–161	0.574		
IAQYTSALL	870–878	0.316		
LYENQKLIA	916–924	0.231		

Putative HLA class I-allele restriction is provided along with their corresponding population frequency in each country (ANFD data).

**Table 6:** Potentially immunodominant T-cell epitope clusters in the spike glycoprotein of SARS-CoV-2, predicted by Predivac-3.0 to yield coverages ≥ 80% in the populations of the United Kingdom, South Africa, Brazil and Japan.

Cluster			T-cell epitopes	
Sequence	Amino acid position	Population coverage (%)	CD8+ T-cell epitopes	CD4+ T-cell epitopes
**The United Kingdom**				
GTKRFDN**PVLPFNDGV**YFASTEK	82–90 (75–97)	99.9	GTKRFDNPV,RFDNPVLPF, LPFNDGVYF,GVYFASTEK	–
KIY**SKHTPINLVRDLP**QGF	205–217 (202–220)	85.0	KIYSKHTPI,YSKHTPINL, TPINLVRDL,LVRDLPQGF	–
NENGT**ITDAVDCALDPLSETKCTL**KSFTVEK	285–303 (280–310)	93.7	NENGTITDA,ITDAVDCAL, AVDCALDPL,ALDPLSETK, ETKCTLKSF,TLKSFTVEK	–
NYLYRLF**RKSNLKPFE**RDISTEI	457–465 (450–472)	90.6	NYLYRLFRK,RLFRKSNLK, KSNLKPFER,FERDISTEI	–
SVAYSNN**SIAIPTNFT**ISVTTEI	711–719 (704–726)	88.7	SVAYSNNSI,AYSNNSIAI,IPTNFTISV,FTISVTTEI	–
I**ITTDNTFVSGNCDVVI**GIVNNTV	1115–1130 (1114–1137)	89.7	–	IITTDNTFV,ITTDNTFVS, FVSGNCDVV,VSGNCDVVI, VIGIVNNTV
**South Africa**				
GTKRFDN**PVLPFNDGV**YFASTEK	82–90 (75–97)	99.9	GTKRFDNPV,RFDNPVLPF, VLPFNDGVY,LPFNDGVYF, GVYFASTEK	–
**Brazil**				
GTKRFDN**PVLPFNDGV**YFASTEK	82–90 (75–97)	99.9	GTKRFDNPV,RFDNPVLPF, VLPFNDGVY,LPFNDGVYF, GVYFASTEK	–
VYYHKNN**KSWMESEFRVYSSANNCTFEYVSQPFLMDLEGKQGNFKNLR**EFVF	150–190 (143–194)	84.2	–	VYYHKNNKS,YHKNNKSW,MESEFRVYS,FRVYSSANN, VYSSANNCT,YSSANNCTF, FEYVSQPFL,FLMDLEGKQ, LMDLEGKQG,MDLEGKQGN,FKNLREFVF
YNYLY**RLFRKSNLKPFERD**ISTEI	454–467 (449–472)	84.3	–	YNYLYRLFR,LYRLFRKSN, YRLFRKSNL,LKPFERDIS, FERDISTEI
**Japan**				
ST**QDLFLPFFSNVTWF**HA	52–65 (50–67)	86.8	STQDLFLPF,TQDLFLPFF, LPFFSNVTW,FSNVTWFHA	-
GTKRFDN**PVLPFNDGV**YFASTEK	82–90 (75–97)	99.0	GTKRFDNPV,RFDNPVLPF, LPFNDGVYF,GVYFASTEK	-
EFQ**FCNDPFLGVYYHK**NNK	135–147 (132–150)	93.5	EFQFCNDPF,FQFCNDPFL, FCNDPFLGV,GVYYHKNNK	-
KSWME**SEFRVYSSANNCTFEYVSQPF**L	155–175 (150–176)	93.3	KSWMESEFR,SEFRVYSSA, YSSANNCTF,SANNCTFEY, TFEYVSQPF,FEYVSQPFL,	-
NENGT**ITDAVDCALDPLSET**KCTLKSF	285–300 (280–306)	95.6	NENGTITDA,ITDAVDCAL, AVDCALDPL,ALDPLSETK, ETKCTLKSF	-
SVAYSNN**SIAIPTNFT**ISVTTEI	711–719 (704–726)	92.6	SVAYSNNSI,AYSNNSIAI, IPTNFTISV,FTISVTTEI	-
YEQYIKW**PWYIWLGFI**AGLIAIV	1213–1221 (1206–1228)	85.6	YEQYIKWPW,EQYIKWPWY,WPWYIWLGF,FIAGLIAIV	-
VYYHKNN**KSWMESEFRVYSSANNCTFEYVSQPFLMDLEGKQ**GN	150–183 (143–185)	88.8		VYYHKNNKS,YHKNNKSW,MESEFRVYS,FRVYSSANN, VYSSANNCT,YSSANNCTF, FEYVSQPFL,FLMDLEGKQ, LMDLEGKQG,MDLEGKQGN

In each cluster, the core region of overlapped T-cell epitopes is highlighted in bold/underscored, showing the amino acid position in each protein, the population coverage in the target populations and the CD8+ or CD4+ T-cell epitopes associated to each cluster.

## Discussion

The impact of HLA polymorphism on viral replicative capacity and disease progression has been widely documented in patients infected by HIV-1 (56, 57). For example, HLA-B*57 and HLA-B*27 (protective alleles) are well-known to associate with successful immune control of HIV-1 or slow progression to disease both in Caucasians and African populations, but not in Asians where the frequencies of these alleles are very low (<1%) (58). By contrast, HLA-B*18:01, HLA-B*45:01 and HLA-B*58:02 (disease-susceptible alleles) are strongly associated with high viral load and rapid disease progression in African populations (59). In addition, considerable work has been conducted to determine population-level HLA associations with vaccine-induced immune responses, which are hypothesized as relevant parameters contributing to vaccine failure (17, 60). For example, the lack of response of a recombinant HIV-1 vaccine (ALVAC Env-gp120) designed to induce clade-specific neutralizing antibodies to HIV-1 in the RV144 phase III trial (Thailand) was strongly associated with the presence of certain HLA class II alleles (DRB1*11 and DRB1*16:02) (18), and a recent study assessing the relationship between HLA genotypes and RTS,S vaccine-mediated protection (malaria infections) showed a strong protective association with three allele groups (HLA-A*01, HLA-B*08, and HLA-DRB1*15/*16) (61). In the latter work, the authors discussed the potential impact of these HLA correlations on vaccine immunogenicity and efficacy in risk populations (such as the sub-Saharan African region) where these alleles are present at a lower prevalence than in the UK or USA where these Phase II trials were carried out. This problem is further complicated by the fact that racial and ethnic minority groups generally remain underrepresented in clinical trials (62), limiting the capability to test the efficacy and safety of new clinical interventions across diverse populations and leading to a lack of T-cell data for ethnicities in which viral epidemic currently spreads (15). Therefore, a vaccine that delivers good results for certain groups in Phase I and II trials does not necessarily guarantee strong protective responses in ethnic minority populations that are in more urgent need of new vaccine initiatives (63).

Genetic variability of the HLA system may affect susceptibility and severity of the disease caused by SARS-CoV-2, as recently discussed in a comprehensive *in silico* analysis of viral peptide-HLA class I binding affinity across 145 HLA types (23). Although it is still early within the SARS-CoV-2 pandemic for broad association studies with HLA markers, recent work found that HLA-C*07:29 and HLA-B*15:27 alleles statistically correlated with the occurrence of COVID‐19 from a sample of 82 Chinese individuals with COVID‐19 that were genotyped for HLA‐A, ‐B, ‐C, ‐DRB1, ‐DRB3/4/5, ‐DQA1, ‐DQB1, ‐DPA1, and ‐DPB1 loci (20). In addition, several associations between HLA alleles provide susceptibility [e.g., B*46:01 (24), HLA‐B*07:03 (64) and HLA-DRB1*12 (65)] or protection [e.g., DRB1*03:01 (66)] to the closely related SARS-CoV-1 have been previously described in Asian populations.

Our immunoinformatics approach addresses this challenge by providing a computational framework to deal with the extent of HLA diversity. We have previously reported the pan-specific tools Predivac-1.0 (33) and Predivac-2.0 (32) to aid CD4+ T-cell epitope-based vaccine design in the context of genetically heterogeneous human populations. Herein, we describe a significant enhancement in our Predivac approach to contribute to the development of genetics-driven immunization strategies that take into account the ethnic diversity of T-cell recognition in the population to be vaccinated. Predivac-3.0 enables proteome-wide ethnicity-driven CD8+ and CD4+ T-cell epitope prediction to select promiscuous T-cell epitopes priming broad immune responses in target groups, with an additional focus on the identification of immunologically relevant T-cell epitope-rich antigen regions potentially affording high-coverage in the target population (referred as hotspots). As shown in **Figure 3A**, the method was successfully validated in LOOCV experiments for 45 HLA class I alleles (17,425 peptides) represented in PredivacDB (AUC = 0.8), by employing a highly rigorous LOOCV methodology that involves excluding an individual allele from the database and then evaluating performance using the dataset of high-affinity peptides restricted by that particular allele (positive dataset). In addition, the tool proved outstanding performance in the benchmarks (**Figures 3B, C** **)**, delivering comparable accuracies against top-performing state-of-the art methods using the IEDB-dataset (average AUC = 0.915) and DFRMLI-dataset (average AUC = 0.909). These AUC values (~0.9) are indicative of excellent discrimination capability, while the lesser AUC (0.8) obtained in LOOCV is typically expected as a consequence of the more stringent experimental condition of systematically removing 100% of the data for the specific tested allele. To maintain balanced datasets for AUC calculation (LOOCV and IEDB benchmark), negative examples were taken from the epitope source protein, by splitting the sequence into overlapping peptides of the same length as the epitope and all peptides except the annotated peptide were assumed as negatives.

We then focused on proteome-wide identification of immunodominant hotspots in order to improve the value of the method for vaccination purposes, in agreement with numerous studies supporting that immunodominant T-cell epitopes are not randomly distributed along the protein sequence, but tend to cluster in limited regions of the antigen undergoing efficient processing (39). The rationale behind this approach is that only a few peptides from complete antigens are7nbsp;capable of inducing significant responses following immunization, which are those peptides presented to T-cells in association with HLA class I and II molecules (67). Accordingly, a significant body of literature underscores the influence of three-dimensional structure of antigens over the likelihood of peptides to be proteolytically released from the source protein, either through the proteasome-mediated endogenous pathway (CD8+ T-cell epitopes) (68) and cathepsin-mediated exogenous pathway (69). However, an alternate reasoning path correlates immunodominant hotspots with promiscuous binding in antigen regions containing a certain density of peptides that bind multiple HLA types (40, 70). Because promiscuous peptides can be presented to T-cells by many individuals (promiscuous T-cell recognition), the identification of regions that are highly enriched in MHC ligands holds potential to define population-based biomarkers as correlates of immunological protection to compare candidate vaccines in efficacy clinical trials (39, 71).

An earlier approach to select promiscuous epitopes is based on the concept of supertypes, which are clusters of HLA molecules sharing overlapping peptide repertories (72). Pepvac is a computational tool based on this approach (30), which depends on pre-calculated population coverages for five HLA class I supertypes (A2, A3, A24, B7, and B15) and accounts for five major American ethnic groups (Black, Caucasian, Hispanic, Native American and Asian). By contrast, allele-based selection methods such as Predivac (32, 33) and OptiTope (31) define promiscuous epitopes as those restricted to as many HLA alleles as possible in a given target population. However, instead of providing population coverage as a function of allele frequency distributions, OptiTope performed “allele coverage” by summing up for each locus the fraction of alleles targeted by predicted T-cell epitopes in a given population. Although we could not compare Predivac-3.0 with OptiTope, since the web-based tool is not currently available, our method offers a more accurate framework by implementing a population coverage algorithm (50) based on HLA genotypic frequencies from the AFND (73), which is the most comprehensive repository of immune gene frequencies of worldwide populations. Therefore, our approach takes into account the fact individuals display different sets of HLA alleles with particular binding specificities and expression frequencies that dramatically differ among different ethnicities (32).

HIV-specific T-cell responses play a pivotal role in the anti-HIV immune response (74). Therefore, the successful identification of HIV-1 specific CD8+ T-cell epitopes in the exploratory analysis for the Japanese population lends support to the utility of the tool in ethnicity-driven T-cell epitope discovery (**Figure 4**). We showed Predivac-3.0 was capable of identifying 46 out of 103 immunodominant T-cell epitopes (44.7% efficiency) from the HIV-dataset with default parameters (PPR = 1; PCT = 0%). Prediction accuracies gradually declined as the population coverage threshold (%) increases for each PPR value (1, 2 and 3), accounting for the growing number of CD8+ T-cell epitopes that are missed as the tool stops selecting epitopes below that threshold limit. However, by filtering T-cell epitopes covering ≥ 20% of the Japanese population (PCT = 20%) the universe of predicted CD8+ T-cell epitopes to be searched decreased to 46% (from 373 to 201 epitopes), with a slight reduction in the accuracy (from 44.2 % to 31.8 %). A good trade-off must balance the predictive accuracy with a reasonable amount of predicted T-cell epitopes to be experimentally tested in the laboratory, because this number must be kept low enough to make the tool useful in accelerating epitope discovery. Inset plots in **Figure 4** show that for PCT = 0% the number of putative CD8+ T-cell epitopes increased from 373 (PPR = 1) to 583 (PPR = 2) and to 719 (PPR = 3), increasingly loosing utility for experimental validation purposes. This is considered a good and sensitive result, especially because the HIV-1 positive dataset (103 CD8+ T-cell epitopes) accounts for a tiny portion (2.4%) of the HIV-1 proteome (4234 amino acids), providing insight into the methods capability to guide epitope discovery in population context. Furthermore, the HIV-dataset only accounts for currently characterized immunodominant T-cell epitopes, leading to the reasonable supposition that accuracy could potentially become higher as new Japanese-specific CD8+ T-cell epitopes are identified in the future. This assumption is underscored by comprehensive HIV-specific epitope mapping studies showing CD4+ and CD8+ T-cell responses across the entire viral proteome (75, 76).

An interesting study case to explore the utility of the tool is the mosaic bivalent T-cell vaccine tHIVconsvX, which comprises 5 Gag-specific and 6 Pol-specific T-cell epitopes (**Table S3**) with the ability to suppress HIV-1 replication *in vivo* and correlate with better clinical outcome (low pVLs and high CD4 counts) in treatment-naïve HIV-1 clade B-infected Japanese individuals (55). The reactivity of this vaccine has been recently characterized in the Japanese population, proving that Gag-specific T cell epitopes (5 epitopes) were found restricted by HLA-B* 52:01, HLA-A*02:06, HLA-A*33:03, and HLA-B*40:02, while the Pol-specific T-cell epitopes (6 epitopes) were restricted by HLA-A*24:02, HLA-A*33:03, HLA-B*40:02, HLA-B*40:06, HLA-B*51:01 and HLA-B*52:01. HLA-B*57, HLA-B*58, and HLA-B*27 are well-known protective alleles for AIDS progression in Caucasians and Africans infected with HIV-1 (65–67). However, the less-characterized HLA*B*52:01 allele is prevalent in the Japanese population (68) and the HLA-B*52:01-C*12:02 haplotype has been suggested to be protective in Japanese individuals, where HLA-B*57, HLA-B*58, or HLA-B*27 are present at very low frequencies in this population (50). The CD8+ T-cell epitopes predicted by Predivac-3.0 to cover 79.3% of the Japanese population (YTAFTIPSI; 282-290) was consistently predicted with HLA*B*52 allele restriction, which in previous studies has been associated with low viral loads in HIV-infected Japanese individuals (69) and also elicited HLA-B*52:01-restricted CD8+ T-cells with strong ability to suppress HIV-1 replication in this population (70).

Predivac-3.0 was effective in identifying immunodominant T-cell epitopes in the HIV dataset, but also guided the detection of Japanese-specific T-cell epitope clusters (hotspots) in the HIV-1 proteome (**Figures 5** and **6**), in agreement with a recent work that provided evidence in favor of the utility of immunoinformatics tools to identify these regions exclusively based on promiscuous HLA peptide binding (40). Indeed, the method was sensitive to capture information on the location of additional CD8+ T-cell epitopes from the HIV-dataset that overlapped putative CD8+ and CD4+ T-cell epitope clusters (**Figure 7**). Putative clusters predicted by Predivac-3.0 are additionally colocalized with 4 immunodominant regions (2 in Gag and 2 in Nef) that are broadly recognized by HIV-infected subjects from several ethnicities, showing the clustering of several CD8+ and CD4+ T-cell epitopes predicted by Predivac 3.0 (**Figure 8** **;** **Table S9**). These results lend support to the reactivity of these regions in the Japanese population, but also about their potential for “universal” T-cell-based vaccination against HIV-1 in heterogeneous ethnic populations (15, 77).

An interesting finding of the current study is the detection of strong immunodominant hotspot signals in the regulatory protein Rev, with four T-cell epitope clusters potentially delivering population coverages above 80% in Japan (positions 4–23; 44–77; 71–97; 94–110). These regions hold interest for vaccine development in this particular ethnic population, suggesting crosslinking between CD8+ and CD4+ T-cell epitopes between positions 71 and 110 of the protein. Indeed, the research has largely focused on the assessment of immune responses directed against Nef and late-expressed HIV-1 structural proteins (Gag, Pol and Env), which concentrate the vast majority of well-defined T-cell epitopes (53). By contrast, regulatory (Tat and Rev) and accessory proteins (Vpr, Vpu and Vif) are less frequently targeted by cytotoxic T-cells in study subjects from clinical interventions (75). This result is consistent with previous works suggesting these proteins might be promising targets for vaccine development (78). A substantial amount of evidence points out that cytotoxic T-cell responses directed against non-structural proteins (Tat, Rev, Vpr, Vpu and Vif) contribute importantly to the total magnitude of the HIV-1 specific cellular immune response (79, 80). Because these proteins are expressed earlier in the viral life cycle, their recognition may occur before Nef down-modulates HLA class I molecules on the surface of the infected cells and thus provide a window of opportunity to the immune system to clear the infected cell before the virus is released (81). Another CD8+ T-cell epitope predicted by Predivac-3.0 within a cluster region of Vpr is the HLA-A*02:01-restricted peptide AIIRILQQL (82, 83), which is located in a functionally important region involved with perinuclear localization of the protein (84, 85). This peptide has been shown to correlate inversely with plasma viral load and positively with CD4 count in a study involving a cohort of HIV-1 infected individuals expressing the HLA-A*02:01 allele (80). A study about HLA haplotype frequencies in the Japanese population states that ~80% of this population could be responsive for a vaccine containing T-cell epitopes presented by HLA-A*02:01 (86).

HLA diversity is likewise a crucial host genetic factor in determining variations in the T-cell responses of HIV-infected patients across multiple ethnicities (15). In this regard, the Japanese-specific density maps (T-cell epitope clusters/hotspots) predicted in the HIV-1 proteome are different to those available at Los Alamos HIV Molecular Immunology Database, accounting for population-level specificities in HLA class I and II frequencies. Interestingly, T-cell epitope clusters allowed a higher efficiency (63.6%) in detecting the position of immunodominant T-cell epitopes from the HIV-dataset (by means of colocalization) than that obtained through direct epitope prediction (12.3%), while both approaches delivered similar accuracies around 40–45% (default parameters PPR = 1; PCT = 0%). Ethnic specificity of HIV immunodominant patterns has been also discussed in a meta-analysis of epitope mapping data from three large vaccination clinical trials carried out in different countries (Merck16, HVTN 054 and HVTN 502/Step), which showed that HIV-1 T-cell responses clustered into distinct hotspot patterns associated with study subjects with different ethnic background (39). Similarly, our analysis carried out on the SARS-CoV-2 spike glycoprotein showed immunodominance patterns accounting for population-specific T-cell epitopes and clusters in the four studied populations (The United Kingdom, South Africa, Brazil and Japan), but also for a few putative immunodominant T-cell epitopes and regions that are of interest for “universal” vaccination purposes as they bind to multiple HLA alleles of high prevalence in all the populations (**Figure 9**). This is also consistent with a recent work showing association of COVID-19 disease with disproportionate mortality among ethnic populations, as specifically observed by the lower mortality rate in the Indian and South Asian subcontinent than in the West (19). To the best of our knowledge these sequences and regions have not been previously reported and can be of interest in the light of the ChAdOx1 vaccine development, which is currently undergoing phase III clinical trials in human volunteers (The United Kingdom, South Africa and Brazil). These results might be also of value for the mRNA-1273 vaccine and for other adenoviral vaccine candidates encoding the S protein antigen.

In short, we support the perspective that this is an immunoinformatics approach that can provide valuable knowledge of T-cell epitopes and immunodominant regions (clusters) to help understand how variation in HLA may affect vaccine-induced immune responses in a population context. Understanding this layer of complexity is also relevant in the context of vaccination trials, since underrepresentation of minorities is an issue that might lead to a resulting body of clinical knowledge that is not generalizable (skewed findings) and a lesser discovery rate of protective T-cell epitopes in certain populations. Predivac-3.0 provides tools to guide the discovery of population-specific epitopes and clusters in the context of SARS-CoV-2 and of other emerging pathogens (EIDs), holding potential to improve vaccine design and clinical trial protocols for evaluation of vaccine candidates in individuals with different genetic or ethnic backgrounds (phase II/III trials).

## Conclusions

Population-level HLA associations are crucial factors determining variations of vaccine-induced immune responses across multiple ethnicities. Predivac-3.0 addresses this problem by implementing a computational framework for rational design of CD8+ and CD4+ T-cell epitope-based vaccination, which allows guiding epitope discovery according to HLA allele frequencies in specific ethnic populations. Our immunoinformatics tool showed a strong performance in the identification of CD8+ T-cell epitopes by leave-one-out cross-validation (AUC ~0.8) and comparable accuracies when benchmarked against state-of-the-art pan-specific methods (AUC ~0.9). We further proved that Predivac-3.0 was accurate and sensitive for *in silico* identification of HIV-1 specific CD8+ T-cell epitopes that are immunodominant in the Japanese population. The method also captured information at proteome-level of epitope-rich areas of HLA promiscuity (hotspots), shedding light onto its capability to identify HIV-1 vaccine-induced and protective T-cell epitopes. We finally showed the utility of Predivac-3.0 in the context of the current COVID-19 pandemics, by applying the Epitope Discovery and Epitope Optimization tools to predict comprehensive lists of population-specific T-cell epitopes and clusters in the SARS-CoV-2 spike glycoprotein for the countries where phase III clinical trials of the ChAdOx1 vaccine are currently being carried out. Putative T-cell epitopes identified for HIV-1 and SARS-CoV-2 are suitable candidates to be experimentally tested for effective vaccine protection, as they hold the potential to induce broad immune responses in the corresponding target populations. In addition, proteome-wide plots (Circos and hotspots) not only allowed for better visualization of the predictions, but also provide the ability to capture knowledge on ligand enrichment areas (based on promiscuous HLA peptide binding) and to detect interactions between the distribution and density of both ethnicity-driven CD8+ and CD4+ T-cell epitopes. Overall, we propose that incorporation of knowledge about HLA prevalence in the target population and immunological hotspots into the predictive algorithm might contribute to the development of novel vaccination strategies that support a more prominent role of T-cell mediated immune responses against emerging viral pathogens, as well as to gain understanding on how variation in HLA may affect vaccine-induced immune responses in a population context. Our immunoinformatics approach is particularly suited to be applied for EIDs associated with well-defined regions or countries, as it accounts for ethnic-level variations of immune responsiveness in the populations in need of vaccination.

## Data Availability Statement

The original contributions presented in the study are included in the article/**Supplementary Material**. Further inquiries can be directed to the corresponding author.

## Author Contributions

PO: conceived, designed, supervised the experiment, and wrote the manuscript. MK: performed *in silico* experiments and prepared the figures. VF: rewrote the Predivac code in Python and performed *in silico* experiments. PO, MK, and VF: analyzed the data. All authors contributed to the article and approved the submitted version.

## Funding

This research was supported by ANID Chile/FONDECYT Iniciación (Grant No.11170638) and by supercomputing infrastructure from UDEC Southern GPU-cluster (FONDEQUIP EQM150134).

## Conflict of Interest

The authors declare that the research was conducted in the absence of any commercial or financial relationships that could be construed as a potential conflict of interest.

## Footnotes

1
http://www.compbio.dundee.ac.uk/aacon/

2
http://www.allelefrequencies.net/

3
http://projects.met-hilab.org/DFRMLI/

4
http://www.hiv.lanl.gov/content/immunology/
