# Supplementary material for: A Proteome-Wide Immunoinformatics Tool to Accelerate T-Cell Epitope Discovery and Vaccine Design in the Context of Emerging Infectious Diseases: An Ethnicity-Oriented Approach
Source: Front Immunol. 2021 Feb 26;12:598778. doi: 10.3389/fimmu.2021.598778 (PMC7952308; doi:10.3389/fimmu.2021.598778)
Supplement: Supplementary file 3 [file DataSheet_3.docx]

Supplementary Material

**Table S1**. Crystal structures of peptide:MHC class I complexes employed to determine specificity-determining residues (SDRs).

| **MHC allele** | **PDB ID** | **Resolution (Å)** | **Bound peptide** | **Protein source** | **UniProtKB** | **Position** | **References** |
| --- | --- | --- | --- | --- | --- | --- | --- |
| A*0101 | 3BO8 | 1.8 | EADPTGHSY | Melanoma-associated antigen 1 (MAGE-1) | P43355 | 362-370 | (1) |
| A*0201 | 1HHG | 2.6 | TLTSCNTSV | HIV-1 gp120 | P04582 | 197-205 | (2) |
|  | 1HHH | 3.0 | FLPSDFFPSV | HBV capside protein | P12901 | 18-27 | (2) |
|  | 1HHI | 2.5 | GILGFVFTL | Influenza A matrix protein M1 | P03485 | 58-66 | (2) |
|  | 1HHJ | 2.5 | ILKEPVHGV | HIV-1 reverse transcriptase | P04588 | 476-484 | (2) |
|  | 1HHK | 2.5 | LLFGYPVYV | protein Tax-1 from Human T-cell leukemia virus 1 (HTLV-1 Tax) | P14079 | 11-19 | (2) |
|  | 2CLR | 2.0 | MLLSVPLLLG | Calreticulina | P27797 | leader peptide | (3) |
|  | 2GUO | 1.9 | AAGIGILTV | Melanoma antigen recognized by T-cells 1 (Melan-A/MART-1) | Q16655 | 27-35 | (4) |
|  | 1TVB | 1.8 | ITDQVPFSV | Melanoma Antigen gp100 | P40967 | 209-2017 | (4) |
| A*1101 | 1Q94 | 2.4 | AIFQSSMTK | HIV-1 POL polyprotein | P12499 | 313-321 | (5) |
|  | 1X7Q | 1.45 | KTFPPTEPK | SARS nucleocapsid | P59595 | 209-217 | (6) |
| A*2402 | 2BCK | 2.8 | VYGFVRACL | Telomerase reverse transcriptase | O14746 |  | (7) |
| A*6801 | 1TMC | 2.3 | EVAPPEYHRK | Galactoside O-acetyltransferase | - | - | (8) |
| B*0801 | 1AGD | 2.05 | GGKKKYKL | HIV-1 gag protein | - | 24-31 | (9) |
|  | 1M05 | 1.9 | FLRGRAYGL | Epstein-Barr virus nuclear antigen-3 (EBNA-3 nuclear protein) | P12977 |  | (10) |
| B*1402 | 3BVN | 2.55 | RRRWRRLTV | membrane protein 2 peptide (LMP2) of Epstein-Barr virus | P13285 | 236-244 | (1) |
|  | 3BXN | 1.86 | IRAAPPPLF | Cathepsin A signal sequence octapeptide |  | 2-10 | (1) |
| B*1501 | 1XR8 | 2.3 | LEKARGSTY | Epstein-Barr virus nuclear antigen-3 (EBNA-3 nuclear protein) | P12977 | 274-282 | (11) |
|  | 1XR9 | 1.8 | ILGPPGSVY | Ubiquitin-conjugating enzyme-E2 | P51965 | 91-99 | (11) |
| B*2705 | 1UXS | 1.55 | RRRWRRLTV | Epstein-Barr virus membrane protein LMP-2A/LMP-2B | P13285 | 236-244 | (12) |
|  | 1JGE | 2.1 | GRFAAAIAK | Synthetic peptide | - | - | (13) |
|  | 1OGT | 1.47 | RRKWRRWHL | Vasoactive intestinal peptide type 1 receptor (VIPR) | P32241 | 400-408 | (13) |
|  | 2A83 | 1.4 | RRRWHRWRL | The glucagon receptor (GR) | P47871 | 412-420 | (14) |
|  | 2BSR | 2.3 | RRIYDLIEL | Epstein-Barr nuclear antigen-6 | P03204 | 258-266 | (15) |
|  | 2BSS | 2.0 | KRWIILGLNK | HIV-1 gag protein | Q98Y46 | 264-273 | (15) |
|  | 2BST | 2.1 | SRYWAIRTR | Influenza A nucleoprotein (NP) | P03466 | 383-391 | (15) |
|  | 3BP4 | 1.85 | IRAAPPPLF | Lysosomal protective protein (Cathepsin A) | P10619 | 2-10 | (1) |
| B*2709 | 1OF2 | 2.2 | RRKWRRWHL | Vasoactive intestinal peptide type 1 receptor (VIPR) | P32241 | 400-408 | (13) |
|  | 3CZF | 1.8 | RRRWHRWRL | Glucagon receptor | P47871 | 412-420 | (16) |
|  | 3D18 | 1.74 | RRRWRRLTL | Epstein–Barr virus (EBV) latent membrane protein 2 (LMP2) | P13285 |  | (16) |
| B*3501 | 1A1N | 2.0 | VPLRPMTY | HIV-1 Nef |  | 74-81 | (9) |
|  | 1QEW | 2.2 | FLWGPRALV | Melanoma-associated antigen 3 | P43357 | 271-279 | (17) |
|  | 1ZHK | 1.6 | LPEPLPQGQLTAY | Trans-activator protein BZLF1 from Human herpesvirus 4 (Epstein Barr virus). | P03206 | 52-64 | (18) |
|  | 1ZSD | 1.7 | EPLPQGQLTAY | Trans-activator protein BZLF1 from Human herpesvirus 4 (Epstein Barr virus). | P03206 | 54-64 | (19) |
|  | 2CIK | 1.75 | KPIVVLHGY | Cytochrome P450 | P11712 | 72-80 | (20) |
|  | 2AXG | 1.8 | APQPAPENAY | Trans-activator protein BZLF1 from Human herpesvirus 4 (Epstein Barr virus). | P03206 | 77-86 | (21) |
| B*3508 | 1ZHL | 1.5 | LPEPLPQGQLTAY | EBV antigen | P03206 | 52-64 | (18) |
|  | 2AXF | 2.0 | APQPAPENAY | Trans-activator protein BZLF1 from Human herpesvirus 4 (Epstein Barr virus). | P03206 | 77-86 | (18) |
|  | 2FZ3 | 1.9 | HPVGEADYFEY | Epstein-Barr nuclear antigen 1 (EBNA1) | P03211 | 407-417 | (22) |
|  | 2NW3 | 1.7 | EPLPQGQLTAY | Trans-activator protein BZLF1 from Human herpesvirus 4 (Epstein Barr virus). | P03206 | 54-64 | (21) |
|  | 3BW9 | 1.75 | CPSQEPMSIYVY | CPS peptide from 65 kDa lower matrix phosphoprotein (pp65 protein) | P18139 | 93-104 | (23) |
| B*4402 | 1M6O | 1.6 | EEFGRAFSF | HLA DPA*0201 protein | Q95HB9 | 77-85 | (24) |
|  | 3DX6 | 1.7 | EENLLDFVRF | Epstein-Barr nuclear antigen 6 | P03204 | 281-290 | (25) |
| B*4403 | 1N2R | 1.7 | EEFGRAFSF | HLA DPA*0201 protein | Q95HB9 | 77-85 | (24) |
|  | 1SYS | 2.4 | EEPTVIKKY | Sorting nexin 5 | Q9Y5X3 | 257-265 | (26) |
| B*4405 | 1SYV | 1.7 | EEFGRAYGF | MHC clase II DR alfa | Q9TQB0 | 37-44 | (26) |
|  | 3DX8 | 2.1 | EENLLDFVRF | Epstein-Barr nuclear antigen 6 | P03204 | 281-290 | (25) |
| B*5101 | 1E27 | 2.2 | LPPVVAKEI | HIV-1 Gag-Pol polyprotein | P24740 | 1168-1176 | (27) |
|  | 1E28 | 3.0 | TAFTIPSI | HIV-1 Gag-Pol polyprotein | P24740 | 708-715 | (27) |
| B*5301 | 1A1M | 2.3 | TPYDINQML | HIV-2 gag protein | P15832 | 182-190 | (28) |
|  | 1A1O | 2.3 | KPIVQYDNF | Liver-stage antigen (LSA)-1 from malaria parasite P. falciparum | GenBank CAA39663.1 | 1786-1794 | (28) |
| B*5701 | 2FRX | 2.5 | LSSPVTKSF | Immunoglobulin kappa constant | P01834 | 94-102 | (29) |
| B*5703 | 2BVO | 1.65 | KAFSPEVIPMF | HIV-1 Gag protein | Q70A61 | 48-58 | (15) |
|  | 2BVP | 1.35 | ISPRTLNAW | HIV-1 Gag protein | Q70A61 | 33-41 | (15) |
|  | 2BVQ | 2.0 | KAFSPEVI | HIV-1 Gag protein | Q70A61 | 48-55 | (15) |
|  | 2HJK | 1.85 | KGFNPEVIPMF | HIV-1 Gag protein | Q70AA1 | 51-61 | (30) |
|  | 2HJL | 1.5 | KAFNPEIIPMF | HIV-1 Gag protein | Q70AA1 | 51-61 | (30) |

**References**

1. Kumar P, Vahedi-Faridi A, Saenger W, Ziegler A, Uchanska-Ziegler B. Conformational changes within the HLA-A1:MAGE-A1 complex induced by binding of a recombinant antibody fragment with TCR-like specificity. *Protein Sci* (2009) 18:37–49. doi:10.1002/pro.4

2. Madden DR, Garboczi DN, Wiley DC. The antigenic identity of peptide-MHC complexes: a comparison of the conformations of five viral peptides presented by HLA-A2. *Cell* (1993) 75:693–708. doi:10.1016/0092-8674(93)90490-h

3. Collins EJ, Garboczi DN, Wiley DC. Three-dimensional structure of a peptide extending from one end of a class I MHC binding site. *Nature* (1994) 371:626–629. doi:10.1038/371626a0

4. Borbulevych OY, Baxter TK, Yu Z, Restifo NP, Baker BM. Increased immunogenicity of an anchor-modified tumor-associated antigen is due to the enhanced stability of the peptide/MHC complex: implications for vaccine design. *J Immunol* (2005) 174:4812–4820. doi:10.4049/jimmunol.174.8.4812

5. Li L, Bouvier M. Structures of HLA-A*1101 complexed with immunodominant nonamer and decamer HIV-1 epitopes clearly reveal the presence of a middle, secondary anchor residue. *J Immunol* (2004) 172:6175–6184. doi:10.4049/jimmunol.172.10.6175

6. Blicher T, Kastrup JS, Buus S, Gajhede M. High-resolution structure of HLA-A*1101 in complex with SARS nucleocapsid peptide. *Acta Crystallogr D Biol Crystallogr* (2005) 61:1031–1040. doi:10.1107/S0907444905013090

7. Cole DK, Rizkallah PJ, Gao F, Watson NI, Boulter JM, Bell JI, Sami M, Gao GF, Jakobsen BK. Crystal structure of HLA-A*2402 complexed with a telomerase peptide. *Eur J Immunol* (2006) 36:170–179. doi:10.1002/eji.200535424

8. Collins EJ, Garboczi DN, Karpusas MN, Wiley DC. The three-dimensional structure of a class I major histocompatibility complex molecule missing the alpha 3 domain of the heavy chain. *Proc Natl Acad Sci U S A* (1995) 92:1218–1221.

9. Reid SW, McAdam S, Smith KJ, Klenerman P, O’Callaghan CA, Harlos K, Jakobsen BK, McMichael AJ, Bell JI, Stuart DI, et al. Antagonist HIV-1 Gag Peptides Induce Structural Changes in HLA B8. *J Exp Med* (1996) 184:2279–2286.

10. Kjer-Nielsen L, Clements CS, Brooks AG, Purcell AW, Fontes MR, McCluskey J, Rossjohn J. The structure of HLA-B8 complexed to an immunodominant viral determinant: peptide-induced conformational changes and a mode of MHC class I dimerization. *J Immunol* (2002) 169:5153–5160. doi:10.4049/jimmunol.169.9.5153

11. Røder G, Blicher T, Justesen S, Johannesen B, Kristensen O, Kastrup J, Buus S, Gajhede M. Crystal structures of two peptide-HLA-B*1501 complexes; structural characterization of the HLA-B62 supertype. *Acta Crystallogr D Biol Crystallogr* (2006) 62:1300–1310. doi:10.1107/S0907444906027636

12. Fiorillo MT, Rückert C, Hülsmeyer M, Sorrentino R, Saenger W, Ziegler A, Uchanska-Ziegler B. Allele-dependent Similarity between Viral and Self-peptide Presentation by HLA-B27 Subtypes. *J Biol Chem* (2005) 280:2962–2971. doi:10.1074/jbc.M410807200

13. Hülsmeyer M, Fiorillo MT, Bettosini F, Sorrentino R, Saenger W, Ziegler A, Uchanska-Ziegler B. Dual, HLA-B27 subtype-dependent conformation of a self-peptide. *J Exp Med* (2004) 199:271–281. doi:10.1084/jem.20031690

14. Rückert C, Fiorillo MT, Loll B, Moretti R, Biesiadka J, Saenger W, Ziegler A, Sorrentino R, Uchanska-Ziegler B. Conformational dimorphism of self-peptides and molecular mimicry in a disease-associated HLA-B27 subtype. *J Biol Chem* (2006) 281:2306–2316. doi:10.1074/jbc.M508528200

15. Stewart-Jones GBE, Gillespie G, Overton IM, Kaul R, Roche P, McMichael AJ, Rowland-Jones S, Jones EY. Structures of three HIV-1 HLA-B*5703-peptide complexes and identification of related HLAs potentially associated with long-term nonprogression. *J Immunol* (2005) 175:2459–2468. doi:10.4049/jimmunol.175.4.2459

16. Beltrami A, Gabdulkhakov A, Rossmann M, Ziegler A, Uchanska-Ziegler B, Saenger W. RCSB PDB - 3D18: Crystal structure of HLA-B*2709 complexed with a variant of the latent membrane protein 2 peptide (LMP2(L)) of epstein-barr virus. Available at: https://www.rcsb.org/structure/3D18 [Accessed August 8, 2020]

17. Orth P, Alings C, Saenger W, Ziegler A. RCSB PDB - 1QEW: Human class I histocompatibility antigen (HLA-A*0201) complex with a nonameric peptide from melanoma-associated antigen 3 (residues 271-279). Available at: https://www.rcsb.org/structure/1qew [Accessed August 8, 2020]

18. Tynan FE, Borg NA, Miles JJ, Beddoe T, El-Hassen D, Silins SL, van Zuylen WJM, Purcell AW, Kjer-Nielsen L, McCluskey J, et al. High resolution structures of highly bulged viral epitopes bound to major histocompatibility complex class I. Implications for T-cell receptor engagement and T-cell immunodominance. *J Biol Chem* (2005) 280:23900–23909. doi:10.1074/jbc.M503060200

19. Miles JJ, Elhassen D, Borg NA, Silins SL, Tynan FE, Burrows JM, Purcell AW, Kjer-Nielsen L, Rossjohn J, Burrows SR, et al. CTL recognition of a bulged viral peptide involves biased TCR selection. *J Immunol* (2005) 175:3826–3834. doi:10.4049/jimmunol.175.6.3826

20. Hourigan CS, Harkiolaki M, Peterson NA, Bell JI, Jones EY, O’Callaghan CA. The structure of the human allo-ligand HLA-B*3501 in complex with a cytochrome p450 peptide: steric hindrance influences TCR allo-recognition. *Eur J Immunol* (2006) 36:3288–3293. doi:10.1002/eji.200636234

21. Tynan FE, Reid HH, Kjer-Nielsen L, Miles JJ, Wilce MCJ, Kostenko L, Borg NA, Williamson NA, Beddoe T, Purcell AW, et al. A T cell receptor flattens a bulged antigenic peptide presented by a major histocompatibility complex class I molecule. *Nat Immunol* (2007) 8:268–276. doi:10.1038/ni1432

22. Miles JJ, Borg NA, Brennan RM, Tynan FE, Kjer-Nielsen L, Silins SL, Bell MJ, Burrows JM, McCluskey J, Rossjohn J, et al. TCR alpha genes direct MHC restriction in the potent human T cell response to a class I-bound viral epitope. *J Immunol* (2006) 177:6804–6814. doi:10.4049/jimmunol.177.10.6804

23. Wynn KK, Fulton Z, Cooper L, Silins SL, Gras S, Archbold JK, Tynan FE, Miles JJ, McCluskey J, Burrows SR, et al. Impact of clonal competition for peptide-MHC complexes on the CD8+ T-cell repertoire selection in a persistent viral infection. *Blood* (2008) 111:4283–4292. doi:10.1182/blood-2007-11-122622

24. Macdonald WA, Purcell AW, Mifsud NA, Ely LK, Williams DS, Chang L, Gorman JJ, Clements CS, Kjer-Nielsen L, Koelle DM, et al. A Naturally Selected Dimorphism within the HLA-B44 Supertype Alters Class I Structure, Peptide Repertoire, and T Cell Recognition. *J Exp Med* (2003) 198:679–691. doi:10.1084/jem.20030066

25. Archbold JK, Macdonald WA, Gras S, Ely LK, Miles JJ, Bell MJ, Brennan RM, Beddoe T, Wilce MCJ, Clements CS, et al. Natural micropolymorphism in human leukocyte antigens provides a basis for genetic control of antigen recognition. *J Exp Med* (2009) 206:209–219. doi:10.1084/jem.20082136

26. Zernich D, Purcell AW, Macdonald WA, Kjer-Nielsen L, Ely LK, Laham N, Crockford T, Mifsud NA, Bharadwaj M, Chang L, et al. Natural HLA class I polymorphism controls the pathway of antigen presentation and susceptibility to viral evasion. *J Exp Med* (2004) 200:13–24. doi:10.1084/jem.20031680

27. Maenaka K, Maenaka T, Tomiyama H, Takiguchi M, Stuart DI, Jones EY. Nonstandard peptide binding revealed by crystal structures of HLA-B*5101 complexed with HIV immunodominant epitopes. *J Immunol* (2000) 165:3260–3267. doi:10.4049/jimmunol.165.6.3260

28. Smith KJ, Reid SW, Harlos K, McMichael AJ, Stuart DI, Bell JI, Jones EY. Bound water structure and polymorphic amino acids act together to allow the binding of different peptides to MHC class I HLA-B53. *Immunity* (1996) 4:215–228. doi:10.1016/s1074-7613(00)80430-6

29. Chessman D, Kostenko L, Lethborg T, Purcell AW, Williamson NA, Chen Z, Kjer-Nielsen L, Mifsud NA, Tait BD, Holdsworth R, et al. Human leukocyte antigen class I-restricted activation of CD8+ T cells provides the immunogenetic basis of a systemic drug hypersensitivity. *Immunity* (2008) 28:822–832. doi:10.1016/j.immuni.2008.04.020

30. Gillespie GMA, Stewart-Jones G, Rengasamy J, Beattie T, Bwayo JJ, Plummer FA, Kaul R, McMichael AJ, Easterbrook P, Dong T, et al. Strong TCR conservation and altered T cell cross-reactivity characterize a B*57-restricted immune response in HIV-1 infection. *J Immunol* (2006) 177:3893–3902. doi:10.4049/jimmunol.177.6.3893

**Table S2.** Predivac database (PredivacDB), showing for each restricting allele the corresponding number of high-affinity peptide ligands, both for HLA class I and class II alleles.

| **Allele** | **Ligands** | **Allele** | **Ligands** | **Allele** | **Ligands** |
| --- | --- | --- | --- | --- | --- |
| **HLA class I-restricted CD8+ T-cell epitopes** | | | | | |
| HLA-A*01:01 | 506 | HLA-A*30:02 | 337 | HLA-B*27:06 | 2 |
| HLA-A*02:01 | 2649 | HLA-A*31:01 | 927 | HLA-B*27:20 | 84 |
| HLA-A*02:02 | 877 | HLA-A*32:01 | 374 | HLA-B*35:01 | 619 |
| HLA-A*02:03 | 1129 | HLA-A*32:07 | 78 | HLA-B*35:03 | 1 |
| HLA-A*02:05 | 20 | HLA-A*32:15 | 58 | HLA-B*37:01 | 2 |
| HLA-A*02:06 | 1044 | HLA-A*33:01 | 348 | HLA-B*38:01 | 11 |
| HLA-A*02:07 | 7 | HLA-A*66:01 | 4 | HLA-B*39:01 | 231 |
| HLA-A*02:11 | 189 | HLA-A*68:01 | 750 | HLA-B*39:06 | 21 |
| HLA-A*02:12 | 151 | HLA-A*68:02 | 729 | HLA-B*40:01 | 427 |
| HLA-A*02:16 | 83 | HLA-A*68:23 | 74 | HLA-B*40:02 | 139 |
| HLA-A*02:17 | 86 | HLA-A*69:01 | 248 | HLA-B*40:13 | 50 |
| HLA-A*02:19 | 97 | HLA-A*80:01 | 116 | HLA-B*42:01 | 2 |
| HLA-A*02:50 | 87 | HLA-B*07:02 | 772 | HLA-B*44:02 | 205 |
| HLA-A*03:01 | 1187 | HLA-B*08:01 | 562 | HLA-B*44:03 | 173 |
| HLA-A*03:19 | 13 | HLA-B*08:02 | 15 | HLA-B*45:01 | 79 |
| HLA-A*11:01 | 1283 | HLA-B*08:03 | 9 | HLA-B*45:06 | 4 |
| HLA-A*11:02 | 11 | HLA-B*14:02 | 2 | HLA-B*46:01 | 64 |
| HLA-A*23:01 | 326 | HLA-B*15:01 | 984 | HLA-B*48:01 | 71 |
| HLA-A*24:02 | 453 | HLA-B*15:02 | 117 | HLA-B*51:01 | 221 |
| HLA-A*24:03 | 287 | HLA-B*15:03 | 325 | HLA-B*53:01 | 279 |
| HLA-A*25:01 | 61 | HLA-B*15:09 | 28 | HLA-B*54:01 | 133 |
| HLA-A*26:01 | 331 | HLA-B*15:17 | 358 | HLA-B*57:01 | 326 |
| HLA-A*26:02 | 98 | HLA-B*15:42 | 3 | HLA-B*58:01 | 554 |
| HLA-A*26:03 | 33 | HLA-B*18:01 | 178 | HLA-B*73:01 | 16 |
| HLA-A*29:02 | 414 | HLA-B*27:01 | 10 | HLA-B*83:01 | 40 |
| HLA-A*30:01 | 531 | HLA-B*27:05 | 260 |  |  |
| **HLA class II-restricted CD4+ T-cell epitopes** | | | | | |
| HLA-DRB1*01:01 | 815 | HLA-DRB1*07:01 | 192 | HLA-DRB1*13:02 | 73 |
| HLA-DRB1*01:02 | 25 | HLA-DRB1*08:01 | 33 | HLA-DRB1*14:01 | 4 |
| HLA-DRB1*03:01 | 180 | HLA-DRB1*08:02 | 35 | HLA-DRB1*15:01 | 172 |
| HLA-DRB1*04:01 | 450 | HLA-DRB1*09:01 | 11 | HLA-DRB1*15:02 | 7 |
| HLA-DRB1*04:02 | 59 | HLA-DRB1*11:01 | 226 | HLA-DRB1*16:01 | 1 |
| HLA-DRB1*04:03 | 7 | HLA-DRB1*11:02 | 1 | HLA-DRB3*01:01 | 7 |
| HLA-DRB1*04:04 | 102 | HLA-DRB1*11:03 | 1 | HLA-DRB3*03:01 | 1 |
| HLA-DRB1*04:05 | 75 | HLA-DRB1*11:04 | 17 | HLA-DRB4*01:01 | 49 |
| HLA-DRB1*04:06 | 8 | HLA-DRB1*12:01 | 9 | HLA-DRB5*01:01 | 109 |
| HLA-DRB1*04:07 | 9 | HLA-DRB1*13:01 | 17 |  |  |

**Table S3**. Multi-ethnic immunodominant CD8+ T-cell epitope clusters recognized in HIV-tested subjects from African-American, Caucasian, Hispanic and West Indian ethnicities (1).

| **Protein** | **Amino acid position** | **Immunodominant region** |
| --- | --- | --- |
| Env | 30-70 | AAEQLWVTVYYGVPVWKEATTTLFCASDACAYDTEVHNVWA |
| Gag | 70-103 | TGSEELRSLYNTVATLYCVHQRIEVKDTKEALEK |
| Gag | 155-203 | WVKVVEEKAFSPEVIPMFSALSEGATPQDLNTMLNTVGGHQAAMQMLKE |
| Gag | 201-241 | LKETINEEAAEWDRVHPVHAGPIAPGQMREPRGSDIAGTTS |
| Gag | 249-291 | WMTNNPPIPVGEIYKRWIILGLNKIVRMYSPTSILDIRQGPKE |
| Gag | 288-330 | GPKEPFRDYVDRFYKTLRAEQASQEVKNWMTETLLVQNANPDC |
| Gag | 427-453 | TRRQANFLGKIWPSHKGRPGNFLQSRP |
| Nef | 57-114 | WLEAQEEEEVGFPVRPQVPLRPMTYKAAVDLSHFLKEKGGLEGLIYSQKRQDILDLWV |
| Nef | 104-157 | QKRQDILDLWVYHTQGYFPDWQNYTPGPGIRYPLTFGWCFKLVPVEPEKVEEAN |
| Vpr | 25-51 | ELKNEAVRHFPRIWLHSLGQHIYETYG |

**Reference**

1. Frahm N, Korber BT, Adams CM, Szinger JJ, Draenert R, Addo MM, Feeney ME, Yusim K, Sango K, Brown NV, et al. Consistent Cytotoxic-T-Lymphocyte Targeting of Immunodominant Regions in Human Immunodeficiency Virus across Multiple Ethnicities. *J Virol* (2004) 78:2187–2200. doi:10.1128/JVI.78.5.2187-2200.2004

**Table S4**. Japanese-specific immunodominant CD8+ T-cell epitopes identified in Gag and Pol proteins, with the ability to induce a protective response in subjects immunized with the T-cell mosaic vaccine tHIVconsvX (1,2).

| **Protein** | **Amino acid position** | **T-cell epitope** |
| --- | --- | --- |
| Gag | 341-349 | ATLEEMMTA |
| Gag | 427-434 | TERQANFL |
| Gag | 316-323 | WMTETLLV |
| Gag | 275-282 | RMYSPTSI |
| Gag | 400-409 | HIAKNCRAPR |
| Pol | 496-504 | IYQEPFKNL |
| Pol | 872-881 | ELKKIIGQVR |
| Pol | 912-919 | GERIVDII |
| Pol | 919-920 | GERIVDIIA |
| Pol | 743-751 | LPPVVAKEI |
| Pol | 283-290 | TAFTIPSI |

**References**

1. Murakoshi H, Zou C, Kuse N, Akahoshi T, Chikata T, Gatanaga H, Oka S, Hanke T, Takiguchi M. CD8+ T cells specific for conserved, cross-reactive Gag epitopes with strong ability to suppress HIV-1 replication. *Retrovirology* (2018) 15:46. doi:10.1186/s12977-018-0429-y

2. Zou C, Murakoshi H, Kuse N, Akahoshi T, Chikata T, Gatanaga H, Oka S, Hanke T, Takiguchi M. Effective Suppression of HIV-1 Replication by Cytotoxic T Lymphocytes Specific for Pol Epitopes in Conserved Mosaic Vaccine Immunogens. *J Virol* (2019) 93: doi:10.1128/JVI.02142-18

**Table S5.** Predictive accuracy and efficiency delivered by Predivac-3.0 at each Peptide Percentile Rank (PPR) value (1, 2 and 3) in the identification of Japanese-specific CD8+ T-cell epitopes (HIV-dataset), by varying the threshold of individuals potentially covered in this target population between 0-100% (PCT).

| **PPR = 1 (default)** | | | | | |
| --- | --- | --- | --- | --- | --- |
| **PCT** | **Published** | **N° of predicted epitopes** | **Total Published** | **Accuracy** | **Efficiency** |
| 0 | 46 | 374 | 103 | 44.66019417 | 12.29946524 |
| 10 | 35 | 250 | 103 | 33.98058252 | 14 |
| 20 | 32 | 202 | 103 | 31.06796117 | 15.84158416 |
| 30 | 24 | 158 | 103 | 23.30097087 | 15.18987342 |
| 40 | 19 | 111 | 103 | 18.44660194 | 17.11711712 |
| 50 | 13 | 74 | 103 | 12.62135922 | 17.56756757 |
| 60 | 7 | 48 | 103 | 6.796116505 | 14.58333333 |
| 70 | 7 | 33 | 103 | 6.796116505 | 21.21212121 |
| 80 | 4 | 16 | 103 | 3.883495146 | 25 |
| 90 | 2 | 5 | 103 | 1.941747573 | 40 |
| 100 | 0 | 0 | NA | NA | NA |
| **PPR = 2** | | | | | |
| **PCT** | **Published** | **N° of predicted epitopes** | **Total Published** | **Accuracy** | **Efficiency** |
| 0 | 59 | 585 | 103 | 57.2815534 | 10.08547009 |
| 10 | 47 | 415 | 103 | 45.63106796 | 11.3253012 |
| 20 | 41 | 365 | 103 | 39.80582524 | 11.23287671 |
| 30 | 34 | 276 | 103 | 33.00970874 | 12.31884058 |
| 40 | 29 | 220 | 103 | 28.15533981 | 13.18181818 |
| 50 | 24 | 147 | 103 | 23.30097087 | 16.32653061 |
| 60 | 18 | 104 | 103 | 17.47572816 | 17.30769231 |
| 70 | 13 | 79 | 103 | 12.62135922 | 16.4556962 |
| 80 | 6 | 52 | 103 | 5.825242718 | 11.53846154 |
| 90 | 4 | 21 | 103 | 3.883495146 | 19.04761905 |
| 100 | 0 | 0 | NA | NA | NA |
| **PPR = 3** | | | | | |
| **PCT** | **Published** | **N° of predicted epitopes** | **Total Published** | **Accuracy** | **Efficiency** |
| 0 | 69 | 725 | 103 | 66.99029126 | 9.517241379 |
| 10 | 57 | 541 | 103 | 55.33980583 | 10.53604436 |
| 20 | 51 | 490 | 103 | 49.51456311 | 10.40816327 |
| 30 | 42 | 392 | 103 | 40.77669903 | 10.71428571 |
| 40 | 36 | 328 | 103 | 34.95145631 | 10.97560976 |
| 50 | 30 | 237 | 103 | 29.12621359 | 12.65822785 |
| 60 | 27 | 175 | 103 | 26.21359223 | 15.42857143 |
| 70 | 21 | 126 | 103 | 20.38834951 | 16.66666667 |
| 80 | 15 | 89 | 103 | 14.5631068 | 16.85393258 |
| 90 | 6 | 42 | 103 | 5.825242718 | 14.28571429 |
| 100 | 0 | 0 | NA | NA | NA |

**Table S6.** HIV-1 dataset based on HIV-1 specific CD8+ T-cell epitopes that are immunodominant for the Japanese population. The dataset was obtained from Los Alamos HIV Molecular Immunology Database, by selecting experimentally determined epitopes in the context of clinical studies involving Japanese subjects. Predivac-3.0 predictions targeting Japan are presented (bold characters refer to the nonameric binding core matching the published epitopes), population coverage calculated for this population and the corresponding references (Epitope Discovery mode; PPR=1).

| **No.** | **Published**  **epitopes** | **Protein** | **Amino acid position (HXB2)** | **Predicted**  **epitopes** | **Population coverage (%)** | **References** |
| --- | --- | --- | --- | --- | --- | --- |
| 1 | TVYYGVPVW | Env | 37-45 | - | - | (1) |
| 2 | VPVWKEATTTL | Env | 42-52 | - | - | (1,2) |
| 3 | EVHNVWATHA | Env | 63-72 | - | - | (3) |
| 4 | **DPNPQEVVL** | Env | 78-86 | **DPNPQEVVL** | **25.9863** | (1,2,4,5) |
| 5 | **TAVPWNASW** | Env | 606-614 | **TAVPWNASW** | **0.3774** | (1,2) |
| 6 | DLRSLCLFSY | Env | 758-767 | - | - | (6) |
| 7 | **IPRRIRQGL** | Env | 843-851 | **IPRRIRQGL** | **43.9923** | (7) |
| 8 | **RPGGKKKYK** | Gag | 22-30 | **RPGGKKKYK** | **17.3199** | (7) |
| 9 | **KYKLKHIVW** | Gag | 28-36 | **KYKLKHIVW** | 56.4811 | (8,9) |
| 10 | LKHIVWASREL | Gag | 31-41 | - | - | (10) |
| 11 | RELERFAV | Gag | 39-46 | - | - | (10) |
| 12 | **SLYNTVATL** | Gag | 77-85 | **SLYNTVATL** | **44.5183** | (9) |
| 13 | TVATLYCVH | Gag | 81-89 | - | - | (11) |
| 14 | **SPRTLNAWV** | Gag | 148-156 | **SPRTLNAWV** | 32.4645 | (7) |
| 15 | KAFSPEVIPMF | Gag | 162-172 | - | - | (6) |
| 16 | **EVIPMFSAL** | Gag | 167-175 | **EVIPMFSAL** | 51 | (3,6,12) |
| 17 | EG**ATPQDLNTM** | Gag | 177-187 | **ATPQDLNTM** | 0.1788 | (13) |
| 18 | TPQDLNTML | Gag | 180-188 | - | - | (7,13,14) |
| 19 | **DLNTMLNTV** | Gag | 183-191 | **DLNTMLNTV** | 0.1788 | (6) |
| 20 | AMQMLKETI | Gag | 197-205 |  |  | (13) |
| 21 | **KETINEEAA** | Gag | 202-210 | **KETINEEAA** | 22.5229 | (13) |
| 22 | **AEWDRVHPV** | Gag | 210-218 | **AEWDRVHPV** | 94.9018 | (10) |
| 23 | **HPVHAGPIA** | Gag | 216-224 | **HPVHAGPIA** | 25.0245 | (1,2,7) |
| 24 | DIAGTTSTL | Gag | 235-243 | - | - | (6) |
| 25 | T**STLQEQIGW** | Gag | 240-249 | **STLQEQIGW** | 51.4901 | (13,15) |
| 26 | NPPIPVGEIY | Gag | 253-262 | - | - | (1,2,15) |
| 27 | IYKRWIILG | Gag | 261-269 | - | - | (16) |
| 28 | IILGLNKIV | Gag | 266-274 | - | - | (15) |
| 29 | GLNKIVRMY | Gag | 269-277 | - | - | (15) |
| 30 | **RMYSPTSI** | Gag | 275-282 | **RMYSPTSIL** | 3.2856 | (10,11,13,14,15) |
| 31 | YSPTSILDI | Gag | 277-285 | - | - | (10,15) |
| 32 | DYVDRFYKT | Gag | 295-303 | - | - | (15) |
| 33 | DYVDRFYKTLR | Gag | 295-305 | - | - | (15) |
| 34 | VKNWMTETL | Gag | 313-321 | - | - | (17) |
| 35 | WMTETLLV | Gag | 316-323 | - | - | (11,13,14,15) |
| 36 | NANPDCKTI | Gag | 325-333 | - | - | (9) |
| 37 | **NPDCKTILK**AL | Gag | 327-337 | **NPDCKTILK** | 23.3396 | (13,14) |
| 38 | KALGPAATL | Gag | 335-343 | - | - | (15) |
| 39 | **GPAATLEEM** | Gag | 338-346 | **GPAATLEEM** | 46.15 | (14) |
| 40 | ATLEEMMTA | Gag | 341-349 | - | - | (10,1415) |
| 41 | ACQGVGGPGHK | Gag | 349-359 | - | - | (15,18) |
| 42 | **GPGHKARVL** | Gag | 355-363 | **GPGHKARVL** | 11.0205 | (7,15) |
| 43 | TERQANFL | Gag | 427-434 | - | - | (15) |
| 44 | RQANFLGKI | Gag | 429-437 | - | - | (15,17) |
| 45 | **FLGKIWPS** | Gag | 433-440 | **FLGKIWPSY** | 32.975 | (15) |
| 46 | EL**YPLTSLRSL** | Gag | 482-492 | **YPLTSLRSL** | 34.6526 | (6) |
| 47 | **TPQVPLRPM**TY | Nef | 71-81 | **TPQVPLRPM** | 11.0205 | (4) |
| 48 | VPLRPMTY | Nef | 74-81 | - | - | (1,4,19,20,21,22) |
| 49 | **RPMTYKAAV** | Nef | 77-85 | **RPMTYKAAV** | 51.0913 | (7) |
| 50 | **AVDLSHFLK** | Nef | 84-92 | **AVDLSHFLK** | 84.604 | (18) |
| 51 | DL**WIYHTQGYF** | Nef | 111-121 | **WIYHTQGYF** | 2.4892 | (6) |
| 52 | FPDWQNYTP | Nef | 121-129 | - | - | (23) |
| 53 | RYPLTFGW | Nef | 134-141 | - | - | (24) |
| 54 | **YPLTFGWCY** | Nef | 135-143 | **YPLTFGWCY** | 73.4159 | (2,16) |
| 55 | **WQRPLVTI** | Pol | 62-69 | **WQRPLVTIK** | 0.9528 | (25) |
| 56 | EICGHKAIGTV | Pol | 121-131 | - | - | (6) |
| 57 | **FPISPIETV** | Pol | 155-163 | **FPISPIETV** | 44.1213 | (18,22,13) |
| 58 | ETVPVKLKPGM | Pol | 161-171 |  |  | (6) |
| 59 | **ELNKRTQDF** | Pol | 234-242 | **ELNKRTQDF** | 0.7144 | (6) |
| 60 | E**VQLGIPHPA** | Pol | 244-253 | **VQLGIPHPA** | 26.1744 | (6) |
| 61 | **TVLDVGDAY** | Pol | 262-270 | **TVLDVGDAY** | 55.173 | (2,15,26) |
| 62 | **VPLDEDFRK**Y | Pol | 273-282 | **VPLDEDFRK** | 1.373 | (2) |
| 63 | **YTAFTIPSI** | Pol | 282-290 | **YTAFTIPSI** | 79.254 | (10,20,26) |
| 64 | **ETPGIRYQY** | Pol | 293-301 | **ETPGIRYQY** | 55.6509 | (6) |
| 65 | LPQGWKGSPA | Pol | 304-313 | - | - | (23,27) |
| 66 | **SPAIFQSSM** | Pol | 311-319 | **SPAIFQSSM** | 93.7286 | (7,26) |
| 67 | AIFQSSMTK | Pol | 313-321 | - | - | (26) |
| 68 | **KQNPDIVIY** | Pol | 328-336 | **KQNPDIVIY** | 28.5589 | (11) |
| 69 | **NPDIVIYQY** | Pol | 330-338 | **NPDIVIYQY** | 44.7693 | (2,7,26) |
| 70 | VIYQYMDDL | Pol | 334-342 | - | - | (6) |
| 71 | **YQYMDDLYV** | Pol | 336-344 | **YQYMDDLYV** | 38.5281 | (26) |
| 72 | ELRQHLLRW | Pol | 359-367 | - | - | (6) |
| 73 | DIQKLVGKL | Pol | 411-419 | - | - | (6) |
| 74 | EVIPLTEEA | Pol | 446-454 | - | - | (6) |
| 75 | **IPLTEEAEL** | Pol | 448-456 | **IPLTEEAEL** | 2.5897 | (28) |
| 76 | **ILKEPVHGV**YY | Pol | 464-474 | **ILKEPVHGV** | 41.1371 | (11,25) |
| 77 | IQ**KQGQGQWTY** | Pol | 484-494 | **KQGQGQWTY** | 89.8549 | (26) |
| 78 | IYQEPFKNL | Pol | 496-504 | - | - | (26) |
| 79 | DVKQLTEAV | Pol | 519-527 | - | - | (6) |
| 80 | **EPIVGAETF**Y | Pol | 587-596 | **EPIVGAETF** | 76.4398 | (2,15) |
| 81 | FYVDGAANR | Pol | 595-603 | - | - | (10) |
| 82 | **ETKLGKAGY** | Pol | 604-612 | **ETKLGKAGY** | 36.1029 | (6,12) |
| 83 | DTTNQKTEL | Pol | 626-634 | - | - | (6) |
| 84 | EVNIVTDSQY | Pol | 647-656 | - | -- | (6) |
| 85 | SQYALGII | Pol | 654-661 | - | - | (11,25) |
| 86 | QIIEQLIKK | Pol | 675-683 | - | - | (18) |
| 87 | LPPVVAKEI | Pol | 743-751 | - | - | (7,26) |
| 88 | EIVASCDKCQL | Pol | 750-760 |  |  | (6) |
| 89 | **GQVDCSPGI** | Pol | 767-775 | **GQVDCSPGI** | 29.3035 | (10) |
| 90 | LEGKVILVA | Pol | 783-791 | - | - | (13) |
| 91 | HVASGYIEA | Pol | 793-801 | - | - | (23,27) |
| 92 | GYIEAEVI | Pol | 797-804 | - | - | (16) |
| 93 | IEAEVIPAET | Pol | 799-808 | - | - | (14,29) |
| 94 | **ETGQETAYF**LL | Pol | 807-817 | **ETGQETAYF** | 22.3022 | (10,29) |
| 95 | GQETAYFLL | Pol | 809-817 |  |  | (10) |
| 96 | **ELKKIIGQV** | Pol | 872-880 | **ELKKIIGQV** | 0.1788 | (6,26) |
| 97 | EHLKTAVQMA | Pol | 885-894 | - | - | (26) |
| 98 | GERIVDIIA | Pol | 912-920 | - |  | (26) |
| 99 | IIATDIQTK | Pol | 918-926 | - | - | (26) |
| 100 | **TDIQTKEL** | Pol | 921-928 | **ATDIQTKEL** | 2.8952 | (14,29) |
| 101 | LQKQITKI | Pol | 928-935 | - | - | (25) |
| 102 | HPRISSEVHI | Vif | 48-57 | - | - | (7) |
| 103 | AIIRILQQL | Vpr | 59-67 | - | - | (30) |

**References**

1. Murakoshi H, Koyanagi M, Akahoshi T, Chikata T, Kuse N, Gatanaga H, Rowland-Jones SL, Oka S, Takiguchi M. Impact of a single HLA-A*24:02-associated escape mutation on the detrimental effect of HLA-B*35:01 in HIV-1 control. *EBioMedicine* (2018) 36:103–112. doi:10.1016/j.ebiom.2018.09.022

2. Matthews PC, Koyanagi M, Kløverpris HN, Harndahl M, Stryhn A, Akahoshi T, Gatanaga H, Oka S, Juarez Molina C, Valenzuela Ponce H, et al. Differential clade-specific HLA-B*3501 association with HIV-1 disease outcome is linked to immunogenicity of a single Gag epitope. *J Virol* (2012) 86:12643–12654. doi:10.1128/JVI.01381-12

3. Kawashima Y, Satoh M, Oka S, Takiguchi M. Identification and Characterization of HIV-1 Epitopes Presented by HLA-A*2603: Comparison Between HIV-1 Epitopes Presented by A*2601 and A*2603. *Human Immunology* (2005) 66:1155–1166. doi:10.1016/j.humimm.2005.10.015

4. Ueno T, Motozono C, Dohki S, Mwimanzi P, Rauch S, Fackler OT, Oka S, Takiguchi M. CTL-mediated selective pressure influences dynamic evolution and pathogenic functions of HIV-1 Nef. *J Immunol* (2008) 180:1107–1116. doi:10.4049/jimmunol.180.2.1107

5. Ueno T, Idegami Y, Motozono C, Oka S, Takiguchi M. Altering effects of antigenic variations in HIV-1 on antiviral effectiveness of HIV-specific CTLs. *J Immunol* (2007) 178:5513–5523. doi:10.4049/jimmunol.178.9.5513

6. Kawashima Y, Satoh M, Oka S, Shirasaka T, Takiguchi M. Different immunodominance of HIV-1-specific CTL epitopes among three subtypes of HLA-A∗26 associated with slow progression to AIDS. *Biochemical and Biophysical Research Communications* (2008) 366:612–616. doi:10.1016/j.bbrc.2007.11.031

7. Kløverpris HN, Adland E, Koyanagi M, Stryhn A, Harndahl M, Matthews PC, Shapiro R, Walker BD, Ndung’u T, Brander C, et al. HIV Subtype Influences HLA-B*07:02-Associated HIV Disease Outcome. *AIDS Res Hum Retroviruses* (2014) 30:468–475. doi:10.1089/aid.2013.0197

8. Akahoshi T, Chikata T, Tamura Y, Gatanaga H, Oka S, Takiguchi M. Selection and Accumulation of an HIV-1 Escape Mutant by Three Types of HIV-1-Specific Cytotoxic T Lymphocytes Recognizing Wild-Type and/or Escape Mutant Epitopes. *J Virol* (2012) 86:1971–1981. doi:10.1128/JVI.06470-11

9. Yokomaku Y, Miura H, Tomiyama H, Kawana-Tachikawa A, Takiguchi M, Kojima A, Nagai Y, Iwamoto A, Matsuda Z, Ariyoshi K. Impaired Processing and Presentation of Cytotoxic-T-Lymphocyte (CTL) Epitopes Are Major Escape Mechanisms from CTL Immune Pressure in Human Immunodeficiency Virus Type 1 Infection. *J Virol* (2004) 78:1324–1332. doi:10.1128/JVI.78.3.1324-1332.2004

10. Watanabe K, Murakoshi H, Tamura Y, Koyanagi M, Chikata T, Gatanaga H, Oka S, Takiguchi M. Identification of cross-clade CTL epitopes in HIV-1 clade A/E-infected individuals by using the clade B overlapping peptides. *Microbes Infect* (2013) 15:874–886. doi:10.1016/j.micinf.2013.08.002

11. Chikata T, Murakoshi H, Koyanagi M, Honda K, Gatanaga H, Oka S, Takiguchi M. Control of HIV-1 by an HLA-B*52:01-C*12:02 Protective Haplotype. *J Infect Dis* (2017) 216:1415–1424. doi:10.1093/infdis/jix483

12. Satoh M, Takamiya Y, Oka S, Tokunaga K, Takiguchi M. Identification and characterization of HIV-1-specific CD8+ T cell epitopes presented by HLA-A*2601. *Vaccine* (2005) 23:3783–3790. doi:10.1016/j.vaccine.2005.02.022

13. Ondondo B, Murakoshi H, Clutton G, Abdul-Jawad S, Wee EG-T, Gatanaga H, Oka S, McMichael AJ, Takiguchi M, Korber B, et al. Novel Conserved-region T-cell Mosaic Vaccine With High Global HIV-1 Coverage Is Recognized by Protective Responses in Untreated Infection. *Mol Ther* (2016) 24:832–842. doi:10.1038/mt.2016.3

14. Murakoshi H, Akahoshi T, Koyanagi M, Chikata T, Naruto T, Maruyama R, Tamura Y, Ishizuka N, Gatanaga H, Oka S, et al. Clinical Control of HIV-1 by Cytotoxic T Cells Specific for Multiple Conserved Epitopes. *J Virol* (2015) 89:5330–5339. doi:10.1128/JVI.00020-15

15. Murakoshi H, Zou C, Kuse N, Akahoshi T, Chikata T, Gatanaga H, Oka S, Hanke T, Takiguchi M. CD8+ T cells specific for conserved, cross-reactive Gag epitopes with strong ability to suppress HIV-1 replication. *Retrovirology* (2018) 15:46. doi:10.1186/s12977-018-0429-y

16. Fujiwara M, Tanuma J, Koizumi H, Kawashima Y, Honda K, Mastuoka-Aizawa S, Dohki S, Oka S, Takiguchi M. Different Abilities of Escape Mutant-Specific Cytotoxic T Cells To Suppress Replication of Escape Mutant and Wild-Type Human Immunodeficiency Virus Type 1 in New Hosts. *J Virol* (2008) 82:138–147. doi:10.1128/JVI.01452-07

17. Murakoshi H, Kitano M, Akahoshi T, Kawashima Y, Dohki S, Oka S, Takiguchi M. Identification and characterization of 2 HIV-1 Gag immunodominant epitopes restricted by Asian HLA allele HLA-B*4801. *Human Immunology* (2009) 70:170–174. doi:10.1016/j.humimm.2008.12.011

18. Koizumi H, Hashimoto M, Fujiwara M, Murakoshi H, Chikata T, Borghan MA, Hachiya A, Kawashima Y, Takata H, Ueno T, et al. Different in vivo effects of HIV-1 immunodominant epitope-specific cytotoxic T lymphocytes on selection of escape mutant viruses. *J Virol* (2010) 84:5508–5519. doi:10.1128/JVI.02483-09

19. Mwimanzi P, Hasan Z, Tokunaga M, Gatanaga H, Oka S, Ueno T. Naturally arising HIV-1 Nef variants conferring escape from cytotoxic T lymphocytes influence viral entry co-receptor expression and susceptibility to superinfection. *Biochem Biophys Res Commun* (2010) 403:422–427. doi:10.1016/j.bbrc.2010.11.047

20. Motozono C, Yokoyama M, Sato H, Ueno T. Cross-reactivity analysis of T cell receptors specific for overlapping HIV-1 Nef epitopes of different lengths. *Microbes Infect* (2014) 16:320–327. doi:10.1016/j.micinf.2013.12.005

21. Motozono C, Miles JJ, Hasan Z, Gatanaga H, Meribe SC, Price DA, Oka S, Sewell AK, Ueno T. CD8+ T Cell Cross-Reactivity Profiles and HIV-1 Immune Escape towards an HLA-B35-Restricted Immunodominant Nef Epitope. *PLoS One* (2013) 8: doi:10.1371/journal.pone.0066152

22. Hashimoto M, Akahoshi T, Murakoshi H, Ishizuka N, Oka S, Takiguchi M. CTL recognition of HIV-1-infected cells via cross-recognition of multiple overlapping peptides from a single 11-mer Pol sequence. *European Journal of Immunology* (2012) 42:2621–2631. doi:10.1002/eji.201242483

23. Kitano M, Kobayashi N, Kawashima Y, Akahoshi T, Nokihara K, Oka S, Takighuchi M. Identification and characterization of HLA-B*5401-restricted HIV-1-Nef and Pol-specific CTL epitopes. *Microbes and Infection* (2008) 10:764–772. doi:10.1016/j.micinf.2008.04.006

24. Katoh J, Kawana-Tachikawa A, Shimizu A, Zhu D, Han C, Nakamura H, Koga M, Kikuchi T, Adachi E, Koibuchi T, et al. Rapid HIV-1 Disease Progression in Individuals Infected with a Virus Adapted to Its Host Population. *PLoS One* (2016) 11: doi:10.1371/journal.pone.0150397

25. Murakoshi H, Koyanagi M, Chikata T, Rahman MA, Kuse N, Sakai K, Gatanaga H, Oka S, Takiguchi M. Accumulation of Pol Mutations Selected by HLA-B*52:01-C*12:02 Protective Haplotype-Restricted Cytotoxic T Lymphocytes Causes Low Plasma Viral Load Due to Low Viral Fitness of Mutant Viruses. *J Virol* (2017) 91: doi:10.1128/JVI.02082-16

26. Zou C, Murakoshi H, Kuse N, Akahoshi T, Chikata T, Gatanaga H, Oka S, Hanke T, Takiguchi M. Effective Suppression of HIV-1 Replication by Cytotoxic T Lymphocytes Specific for Pol Epitopes in Conserved Mosaic Vaccine Immunogens. *J Virol* (2019) 93: doi:10.1128/JVI.02142-18

27. Hashimoto M, Kitano M, Honda K, Koizumi H, Dohki S, Oka S, Takiguchi M. Selection of escape mutation by Pol154-162-specific cytotoxic T cells among chronically HIV-1-infected HLA-B*5401-positive individuals. *Hum Immunol* (2010) 71:123–127. doi:10.1016/j.humimm.2009.10.015

28. Ueno T, Tomiyama H, Fujiwara M, Oka S, Takiguchi M. HLA class I-restricted recognition of an HIV-derived epitope peptide by a human T cell receptor alpha chain having a Vdelta1 variable segment. *Eur J Immunol* (2003) 33:2910–2916. doi:10.1002/eji.200324148

29. Watanabe T, Murakoshi H, Gatanaga H, Koyanagi M, Oka S, Takiguchi M. Effective recognition of HIV-1-infected cells by HIV-1 integrase-specific HLA-B∗4002-restricted T cells. *Microbes Infect* (2011) 13:160–166. doi:10.1016/j.micinf.2010.10.006

30. Kamori D, Hasan Z, Ohashi J, Kawana-Tachikawa A, Gatanaga H, Oka S, Ueno T. Identification of two unique naturally occurring Vpr sequence polymorphisms associated with clinical parameters in HIV-1 chronic infection. *J Med Virol* (2017) 89:123–129. doi:10.1002/jmv.24612

**Table S7.** Full list of HIV-1 specific CD8+ T-cell epitopes predicted by Predivac-3.0 for the Japanese population, using the Epitope Discovery mode (PPR=1; PCT=0). Reactive T-cell epitopes are highlighted in bold for those predicted to cover ≥ 20% (black) and ≥ 80% (red) of the Japanese population.

| **Protein** | **Start** | **End** | **N° of HLA class I alleles** | **Population**  **coverage (%)** | **Putative CD8+**  **T-cell epitopes** |
| --- | --- | --- | --- | --- | --- |
| **Env** | **7** | **15** | **10** | **33.763** | **YQHLWRWGW** |
| **Env** | **9** | **17** | **6** | **30.3764** | **HLWRWGWRW** |
| Env | 31 | 39 | 7 | 18.7851 | TEKLWVTVY |
| **Env** | **36** | **44** | **13** | **43.3651** | **VTVYYGVPV** |
| **Env** | **60** | **68** | **5** | **55.8057** | **AYDTEVHNV** |
| **Env** | **67** | **75** | **16** | **54.0454** | **NVWATHACV** |
| **Env** | **78** | **86** | **3** | **25.9863** | **DPNPQEVVL** |
| Env | 85 | 93 | 1 | 2.4892 | VLVNVTENF |
| **Env** | **103** | **111** | **4** | **34.6526** | **QMHEDIISL** |
| **Env** | **108** | **116** | **4** | **34.6526** | **IISLWDQSL** |
| **Env** | **117** | **125** | **30** | **85.7145** | **KPCVKLTPL** |
| Env | 119 | 127 | 2 | 1.9585 | CVKLTPLCV |
| **Env** | **121** | **129** | **9** | **42.6344** | **KLTPLCVSL** |
| Env | 139 | 147 | 2 | 9.1446 | NTNSSSGRM |
| Env | 143 | 151 | 4 | 17.9207 | SSGRMIMEK |
| Env | 147 | 155 | 1 | 0.1989 | MIMEKGEIK |
| **Env** | **151** | **159** | **3** | **55.173** | **KGEIKNCSF** |
| Env | 157 | 165 | 1 | 2.3736 | CSFNISTSI |
| Env | 168 | 176 | 3 | 9.7943 | KVQKEYAFF |
| Env | 171 | 179 | 5 | 31.33 | KEYAFFYKL |
| Env | 176 | 184 | 2 | 1.4618 | FYKLDIIPI |
| **Env** | **192** | **200** | **15** | **48.6357** | **KLTSCNTSV** |
| **Env** | **199** | **207** | **10** | **24.5702** | **SVITQACPK** |
| **Env** | **205** | **213** | **12** | **41.8302** | **CPKVSFEPI** |
| **Env** | **207** | **215** | **9** | **66.3863** | **KVSFEPIPI** |
| **Env** | **211** | **219** | **5** | **25.0245** | **EPIPIHYCA** |
| **Env** | **213** | **221** | **15** | **44.4742** | **IPIHYCAPA** |
| **Env** | **219** | **227** | **7** | **23.3396** | **APAGFAILK** |
| Env | 223 | 231 | 1 | 0.9528 | FAILKCNNK |
| Env | 225 | 233 | 7 | 19.481 | ILKCNNKTF |
| Env | 237 | 245 | 2 | 1.9585 | GPCTNVSTV |
| **Env** | **243** | **251** | **21** | **79.7225** | **STVQCTHGI** |
| **Env** | **247** | **255** | **12** | **43.0051** | **CTHGIRPVV** |
| **Env** | **252** | **260** | **20** | **78.7697** | **RPVVSTQLL** |
| Env | 257 | 265 | 1 | 1.3288 | TQLLLNGSL |
| Env | 267 | 275 | 3 | 11.4143 | EEEVVIRSV |
| **Env** | **274** | **282** | **18** | **86.313** | **SVNFTDNAK** |
| Env | 284 | 292 | 1 | 0.1788 | IIVQLNTSV |
| **Env** | **296** | **304** | **5** | **30.9364** | **CTRPNNNTR** |
| Env | 298 | 306 | 6 | 18.5679 | RPNNNTRKR |
| **Env** | **309** | **317** | **14** | **55.1656** | **IQRGPGRAF** |
| Env | 312 | 320 | 1 | 0.477 | GPGRAFVTI |
| **Env** | **318** | **326** | **6** | **28.0844** | **VTIGKIGNM** |
| Env | 325 | 333 | 1 | 2.4892 | NMRQAHCNI |
| **Env** | **327** | **335** | **11** | **49.3563** | **RQAHCNISR** |
| **Env** | **335** | **343** | **4** | **34.2476** | **RAKWNNTLK** |
| **Env** | **340** | **348** | **7** | **36.0043** | **NTLKQIASK** |
| **Env** | **368** | **376** | **18** | **64.8286** | **DPEIVTHSF** |
| **Env** | **374** | **382** | **20** | **62.774** | **HSFNCGGEF** |
| Env | 375 | 383 | 1 | 0.2325 | SFNCGGEFF |
| **Env** | **383** | **391** | **6** | **55.9483** | **FYCNSTQLF** |
| **Env** | **387** | **395** | **24** | **85.5879** | **STQLFNSTW** |
| Env | 392 | 400 | 2 | 3.3541 | NSTWFNSTW |
| Env | 408 | 416 | 3 | 4.1935 | TEGSDTITL |
| Env | 419 | 427 | 7 | 18.7851 | RIKQIINMW |
| **Env** | **421** | **429** | **15** | **52.5427** | **KQIINMWQK** |
| Env | 441 | 449 | 1 | 0.1788 | GQIRCSSNI |
| Env | 492 | 500 | 8 | 19.1017 | EPLGVAPTK |
| **Env** | **511** | **519** | **7** | **28.2343** | **RAVGIGALF** |
| Env | 514 | 522 | 1 | 2.4892 | GIGALFLGF |
| Env | 517 | 525 | 4 | 6.6489 | ALFLGFLGA |
| Env | 557 | 565 | 1 | 2.5897 | RAIEAQQHL |
| **Env** | **562** | **570** | **10** | **24.645** | **QQHLLQLTV** |
| **Env** | **565** | **573** | **8** | **42.9082** | **LLQLTVWGI** |
| **Env** | **575** | **583** | **8** | **41.1371** | **QLQARILAV** |
| Env | 585 | 593 | 3 | 1.1596 | RYLKDQQLL |
| **Env** | **604** | **612** | **5** | **25.0245** | **CTTAVPWNA** |
| Env | 606 | 614 | 2 | 0.3774 | TAVPWNASW |
| **Env** | **620** | **628** | **21** | **63.1368** | **EQIWNHTTW** |
| Env | 627 | 635 | 1 | 0.2146 | TWMEWDREI |
| **Env** | **630** | **638** | **10** | **75.4808** | **EWDREINNY** |
| **Env** | **633** | **641** | **21** | **78.9651** | **REINNYTSL** |
| Env | 637 | 645 | 1 | 1.2485 | NYTSLIHSL |
| **Env** | **647** | **655** | **10** | **24.5702** | **EESQNQQEK** |
| Env | 653 | 661 | 1 | 1.3288 | QEKNEQELL |
| Env | 662 | 670 | 1 | 0.7144 | ELDKWASLW |
| Env | 665 | 673 | 2 | 0.4468 | KWASLWNWF |
| Env | 675 | 683 | 1 | 0.1989 | ITNWLWYIK |
| **Env** | **685** | **693** | **6** | **36.9753** | **FIMIVGGLV** |
| Env | 694 | 702 | 1 | 11.0205 | GLRIVFAVL |
| Env | 696 | 704 | 2 | 6.401 | RIVFAVLSI |
| **Env** | **704** | **712** | **20** | **85.0586** | **IVNRVRQGY** |
| Env | 707 | 715 | 1 | 11.0205 | RVRQGYSPL |
| **Env** | **709** | **717** | **16** | **70.8442** | **RQGYSPLSF** |
| **Env** | **713** | **721** | **11** | **60.2114** | **SPLSFQTHL** |
| Env | 738 | 746 | 2 | 12.2737 | GERDRDRSI |
| Env | 745 | 753 | 1 | 11.0205 | SIRLVNGSL |
| **Env** | **747** | **755** | **10** | **60.0102** | **RLVNGSLAL** |
| Env | 782 | 790 | 1 | 0.7144 | VELLGRRGW |
| **Env** | **784** | **792** | **7** | **40.9998** | **LLGRRGWEA** |
| **Env** | **794** | **802** | **5** | **31.0467** | **KYWWNLLQY** |
| **Env** | **799** | **807** | **9** | **43.0433** | **LLQYWSQEL** |
| **Env** | **804** | **812** | **9** | **22.5579** | **SQELKNSAV** |
| Env | 811 | 819 | 1 | 0.477 | AVSLLNATA |
| **Env** | **814** | **822** | **8** | **41.1371** | **LLNATAIAV** |
| Env | 825 | 833 | 1 | 1.2485 | GTDRVIEVV |
| **Env** | **828** | **836** | **4** | **22.9427** | **RVIEVVQGA** |
| Env | 830 | 838 | 2 | 5.484 | IEVVQGACR |
| Env | 839 | 847 | 1 | 11.0205 | AIRHIPRRI |
| **Env** | **843** | **851** | **6** | **43.9923** | **IPRRIRQGL** |
| Env | 848 | 856 | 1 | 1.3288 | RQGLERILL |
| Gag | 18 | 26 | 2 | 1.4838 | KIRLRPGGK |
| Gag | 22 | 30 | 4 | 17.3199 | RPGGKKKYK |
| **Gag** | **28** | **36** | **5** | **56.4811** | **KYKLKHIVW** |
| **Gag** | **60** | **68** | **9** | **43.4409** | **ILGQLQPSL** |
| **Gag** | **67** | **75** | **9** | **43.0433** | **SLQTGSEEL** |
| Gag | 71 | 79 | 1 | 0.7144 | GSEELRSLY |
| **Gag** | **77** | **85** | **10** | **44.5183** | **SLYNTVATL** |
| Gag | 104 | 112 | 1 | 0.1989 | IEEEQNKSK |
| **Gag** | **124** | **132** | **22** | **63.2337** | **HSNQVSQNY** |
| **Gag** | **126** | **134** | **5** | **31.1485** | **NQVSQNYPI** |
| Gag | 134 | 142 | 5 | 18.0463 | IVQNIQGQM |
| Gag | 135 | 143 | 1 | 1.3288 | VQNIQGQMV |
| Gag | 142 | 150 | 3 | 6.3855 | MVHQAISPR |
| Gag | 144 | 152 | 2 | 12.2737 | HQAISPRTL |
| Gag | 147 | 155 | 4 | 12.3375 | ISPRTLNAW |
| **Gag** | **148** | **156** | **11** | **32.4645** | **SPRTLNAWV** |
| Gag | 150 | 158 | 2 | 1.9585 | RTLNAWVKV |
| Gag | 160 | 168 | 4 | 12.0312 | EEKAFSPEV |
| Gag | 167 | 175 | 11 | 51 | EVIPMFSAL |
| Gag | 179 | 187 | 1 | 0.1788 | ATPQDLNTM |
| Gag | 183 | 191 | 1 | 0.1788 | DLNTMLNTV |
| **Gag** | **189** | **197** | **5** | **25.0245** | **NTVGGHQAA** |
| Gag | 194 | 202 | 1 | 5.2957 | HQAAMQMLK |
| Gag | 202 | 210 | 3 | 22.5229 | KETINEEAA |
| **Gag** | **204** | **212** | **8** | **28.5785** | **TINEEAAEW** |
| **Gag** | **210** | **218** | **31** | **94.9018** | **AEWDRVHPV** |
| **Gag** | **216** | **224** | **5** | **25.0245** | **HPVHAGPIA** |
| Gag | 224 | 232 | 5 | 18.0828 | APGQMREPR |
| **Gag** | **241** | **249** | **14** | **51.4901** | **STLQEQIGW** |
| **Gag** | **248** | **256** | **6** | **56.6226** | **GWMTNNPPI** |
| **Gag** | **250** | **258** | **37** | **97.3786** | **MTNNPPIPV** |
| **Gag** | **264** | **272** | **10** | **75.4808** | **RWIILGLNK** |
| Gag | 275 | 283 | 2 | 3.2856 | RMYSPTSIL |
| **Gag** | **282** | **290** | **13** | **69.5222** | **ILDIRQGPK** |
| **Gag** | **286** | **294** | **4** | **30.5257** | **RQGPKEPFR** |
| **Gag** | **288** | **296** | **12** | **36.2318** | **GPKEPFRDY** |
| Gag | 293 | 301 | 1 | 0.7144 | FRDYVDRFY |
| Gag | 306 | 314 | 3 | 1.6053 | AEQASQEVK |
| **Gag** | **327** | **335** | **7** | **23.3396** | **NPDCKTILK** |
| Gag | 338 | 346 | 10 | 46.15 | GPAATLEEM |
| **Gag** | **345** | **353** | **18** | **54.9017** | **EMMTACQGV** |
| Gag | 355 | 363 | 1 | 11.0205 | GPGHKARVL |
| Gag | 359 | 367 | 3 | 12.5158 | KARVLAEAM |
| Gag | 362 | 370 | 3 | 5.4349 | VLAEAMSQV |
| **Gag** | **368** | **376** | **6** | **38.7524** | **SQVTNSATI** |
| Gag | 375 | 383 | 2 | 1.1683 | TIMMQRGNF |
| **Gag** | **376** | **384** | **3** | **29.823** | **IMMQRGNFR** |
| Gag | 385 | 393 | 6 | 18.6396 | NQRKIVKCF |
| **Gag** | **389** | **397** | **5** | **34.8741** | **IVKCFNCGK** |
| Gag | 407 | 415 | 3 | 1.6053 | APRKKGCWK |
| Gag | 415 | 423 | 1 | 0.933 | KCGKEGHQM |
| **Gag** | **421** | **429** | **5** | **34.6821** | **HQMKDCTER** |
| **Gag** | **433** | **441** | **7** | **32.975** | **FLGKIWPSY** |
| Gag | 440 | 448 | 3 | 1.6925 | SYKGRPGNF |
| Gag | 444 | 452 | 7 | 18.9401 | RPGNFLQSR |
| Gag | 467 | 475 | 2 | 0.6556 | VETTTPPQK |
| **Gag** | **481** | **489** | **16** | **67.6015** | **KELYPLTSL** |
| **Gag** | **484** | **492** | **4** | **34.6526** | **YPLTSLRSL** |
| Nef | 5 | 13 | 1 | 0.6556 | WSKSSVIGW |
| Nef | 24 | 32 | 1 | 2.3736 | EPAADRVGA |
| **Nef** | **50** | **58** | **6** | **37.6351** | **ATNAACAWL** |
| **Nef** | **62** | **70** | **17** | **65.2966** | **EEEEVGFPV** |
| **Nef** | **68** | **76** | **16** | **78.1429** | **FPVTPQVPL** |
| Nef | 71 | 79 | 1 | 11.0205 | TPQVPLRPM |
| **Nef** | **77** | **85** | **16** | **51.0913** | **RPMTYKAAV** |
| **Nef** | **82** | **90** | **9** | **29.9298** | **KAAVDLSHF** |
| **Nef** | **84** | **92** | **15** | **84.604** | **AVDLSHFLK** |
| Nef | 92 | 100 | 2 | 2.2752 | KEKGGLEGL |
| **Nef** | **106** | **114** | **6** | **56.6344** | **RQDILDLWI** |
| Nef | 113 | 121 | 1 | 2.4892 | WIYHTQGYF |
| Nef | 116 | 124 | 5 | 18.0463 | HTQGYFPDW |
| **Nef** | **119** | **127** | **5** | **31.0467** | **GYFPDWQNY** |
| **Nef** | **135** | **143** | **31** | **73.4159** | **YPLTFGWCY** |
| Nef | 140 | 148 | 2 | 5.2608 | GWCYKLVPV |
| **Nef** | **172** | **180** | **8** | **41.1371** | **GMDDPEREV** |
| Nef | 175 | 183 | 3 | 1.3653 | DPEREVLEW |
| **Nef** | **181** | **189** | **4** | **34.6526** | **LEWRFDSRL** |
| Nef | 186 | 194 | 2 | 1.9585 | DSRLAFHHV |
| Nef | 194 | 202 | 2 | 1.1456 | VARELHPEY |
| Nef | 196 | 204 | 1 | 5.2957 | RELHPEYFK |
| Pol | 8 | 16 | 1 | 2.4892 | FLQGKAREF |
| Pol | 14 | 22 | 4 | 17.5358 | REFSSEQTR |
| **Pol** | **25** | **33** | **8** | **21.3802** | **SPTRRELQV** |
| Pol | 40 | 48 | 4 | 6.8045 | SPSEAGADR |
| **Pol** | **48** | **56** | **7** | **56.0954** | **RQGTVSFNF** |
| Pol | 51 | 59 | 2 | 1.9585 | TVSFNFPQV |
| Pol | 56 | 64 | 3 | 1.2141 | FPQVTLWQR |
| Pol | 62 | 70 | 1 | 0.9528 | WQRPLVTIK |
| Pol | 67 | 75 | 1 | 2.5897 | VTIKIGGQL |
| **Pol** | **76** | **84** | **6** | **25.2827** | **KEALLDTGA** |
| Pol | 86 | 94 | 1 | 2.5897 | DTVLEEMSL |
| **Pol** | **90** | **98** | **13** | **32.2676** | **EEMSLPGRW** |
| **Pol** | **101** | **109** | **18** | **47.1093** | **KMIGGIGGF** |
| Pol | 111 | 119 | 1 | 11.0205 | KVRQYDQIL |
| **Pol** | **114** | **122** | **6** | **55.9483** | **QYDQILIEI** |
| Pol | 118 | 126 | 1 | 0.9528 | ILIEICGHK |
| **Pol** | **120** | **128** | **5** | **35.7259** | **IEICGHKAI** |
| Pol | 130 | 138 | 8 | 13.5893 | TVLVGPTPV |
| Pol | 145 | 153 | 3 | 6.476 | LLTQIGCTL |
| **Pol** | **152** | **160** | **7** | **40.1723** | **TLNFPISPI** |
| **Pol** | **155** | **163** | **14** | **44.1213** | **FPISPIETV** |
| Pol | 158 | 166 | 5 | 7.3292 | SPIETVPVK |
| **Pol** | **168** | **176** | **14** | **44.1213** | **KPGMDGPKV** |
| **Pol** | **173** | **181** | **23** | **79.2136** | **GPKVKQWPL** |
| **Pol** | **177** | **185** | **10** | **38.2323** | **KQWPLTEEK** |
| Pol | 190 | 198 | 1 | 5.2957 | VEICTEMEK |
| Pol | 193 | 201 | 1 | 0.1788 | CTEMEKEGK |
| Pol | 196 | 204 | 2 | 6.198 | MEKEGKISK |
| Pol | 207 | 215 | 1 | 1.3288 | PENPYNTPV |
| Pol | 217 | 225 | 1 | 0.9528 | AIKKKDSTK |
| Pol | 229 | 237 | 5 | 18.0828 | LVDFRELNK |
| Pol | 234 | 242 | 1 | 0.7144 | ELNKRTQDF |
| **Pol** | **245** | **253** | **6** | **26.1744** | **VQLGIPHPA** |
| Pol | 249 | 257 | 8 | 19.505 | IPHPAGLKK |
| **Pol** | **262** | **270** | **3** | **55.173** | **TVLDVGDAY** |
| Pol | 263 | 271 | 3 | 4.0979 | VLDVGDAYF |
| Pol | 273 | 281 | 2 | 1.373 | VPLDEDFRK |
| **Pol** | **282** | **290** | **17** | **79.254** | **YTAFTIPSI** |
| **Pol** | **289** | **297** | **5** | **36.0057** | **SINNETPGI** |
| **Pol** | **293** | **301** | **4** | **55.6509** | **ETPGIRYQY** |
| Pol | 296 | 304 | 1 | 11.0205 | GIRYQYNVL |
| Pol | 300 | 308 | 2 | 0.4468 | QYNVLPQGW |
| **Pol** | **311** | **319** | **29** | **93.7286** | **SPAIFQSSM** |
| **Pol** | **319** | **327** | **4** | **30.272** | **MTKILEPFR** |
| **Pol** | **328** | **336** | **5** | **28.5589** | **KQNPDIVIY** |
| **Pol** | **330** | **338** | **12** | **44.7693** | **NPDIVIYQY** |
| Pol | 335 | 343 | 1 | 0.2325 | IYQYMDDLY |
| **Pol** | **336** | **344** | **8** | **38.5281** | **YQYMDDLYV** |
| Pol | 357 | 365 | 6 | 36.98 | IEELRQHLL |
| Pol | 358 | 366 | 3 | 17.1571 | EELRQHLLR |
| **Pol** | **361** | **369** | **29** | **88.9911** | **RQHLLRWGL** |
| **Pol** | **366** | **374** | **8** | **74.7399** | **RWGLTTPDK** |
| **Pol** | **370** | **378** | **4** | **29.9728** | **TTPDKKHQK** |
| Pol | 376 | 384 | 1 | 0.6556 | HQKEPPFLW |
| Pol | 379 | 387 | 3 | 2.0006 | EPPFLWMGY |
| **Pol** | **385** | **393** | **3** | **29.823** | **MGYELHPDK** |
| **Pol** | **401** | **409** | **15** | **60.8239** | **LPEKDSWTV** |
| **Pol** | **406** | **414** | **3** | **29.823** | **SWTVNDIQK** |
| **Pol** | **407** | **415** | **4** | **34.6526** | **WTVNDIQKL** |
| Pol | 418 | 426 | 4 | 6.6543 | KLNWASQIY |
| Pol | 423 | 431 | 1 | 1.3288 | SQIYPGIKV |
| Pol | 426 | 434 | 1 | 11.0205 | YPGIKVRQL |
| Pol | 430 | 438 | 1 | 11.0205 | KVRQLCKLL |
| Pol | 436 | 444 | 3 | 6.1448 | KLLRGTKAL |
| Pol | 448 | 456 | 1 | 2.5897 | IPLTEEAEL |
| **Pol** | **464** | **472** | **8** | **41.1371** | **ILKEPVHGV** |
| **Pol** | **466** | **474** | **8** | **32.922** | **KEPVHGVYY** |
| Pol | 473 | 481 | 1 | 0.2325 | YYDPSKDLI |
| **Pol** | **484** | **492** | **8** | **26.8516** | **IQKQGQGQW** |
| **Pol** | **486** | **494** | **26** | **89.8549** | **KQGQGQWTY** |
| **Pol** | **493** | **501** | **6** | **56.6344** | **TYQIYQEPF** |
| **Pol** | **494** | **502** | **3** | **29.823** | **YQIYQEPFK** |
| Pol | 527 | 535 | 1 | 1.3288 | VQKITTESI |
| **Pol** | **532** | **540** | **5** | **22.3273** | **TESIVIWGK** |
| **Pol** | **535** | **543** | **4** | **30.4917** | **IVIWGKTPK** |
| **Pol** | **540** | **548** | **3** | **55.173** | **KTPKFKLPI** |
| Pol | 545 | 553 | 1 | 0.933 | KLPIQKETW |
| Pol | 548 | 556 | 7 | 19.481 | IQKETWETW |
| **Pol** | **557** | **565** | **6** | **30.9831** | **WTEYWQATW** |
| **Pol** | **561** | **569** | **19** | **62.5723** | **WQATWIPEW** |
| **Pol** | **563** | **571** | **14** | **56.5953** | **ATWIPEWEF** |
| **Pol** | **569** | **577** | **30** | **93.7338** | **WEFVNTPPL** |
| Pol | 571 | 579 | 1 | 0.1989 | FVNTPPLVK |
| **Pol** | **587** | **595** | **21** | **76.4398** | **EPIVGAETF** |
| Pol | 589 | 597 | 2 | 5.2608 | IVGAETFYV |
| **Pol** | **593** | **601** | **5** | **25.0245** | **ETFYVDGAA** |
| **Pol** | **604** | **612** | **11** | **36.1029** | **ETKLGKAGY** |
| Pol | 629 | 637 | 1 | 1.3288 | NQKTELQAI |
| **Pol** | **638** | **646** | **9** | **43.0433** | **YLALQDSGL** |
| **Pol** | **640** | **648** | **8** | **41.1371** | **ALQDSGLEV** |
| Pol | 650 | 658 | 1 | 2.5897 | IVTDSQYAL |
| **Pol** | **664** | **672** | **4** | **34.6526** | **QPDQSESEL** |
| **Pol** | **668** | **676** | **4** | **34.6526** | **SESELVNQI** |
| Pol | 683 | 691 | 1 | 1.3288 | KEKVYLAWV |
| **Pol** | **685** | **693** | **5** | **25.0245** | **KVYLAWVPA** |
| Pol | 687 | 695 | 1 | 2.4892 | YLAWVPAHK |
| **Pol** | **701** | **709** | **5** | **25.0245** | **EQVDKLVSA** |
| Pol | 735 | 743 | 5 | 31.33 | RAMASDFNL |
| Pol | 738 | 746 | 4 | 11.9146 | ASDFNLPPV |
| **Pol** | **749** | **757** | **7** | **23.2232** | **KEIVASCDK** |
| Pol | 753 | 761 | 1 | 0.8392 | ASCDKCQLK |
| **Pol** | **767** | **775** | **4** | **29.3035** | **GQVDCSPGI** |
| Pol | 786 | 794 | 2 | 5.2608 | KVILVAVHV |
| Pol | 806 | 814 | 3 | 0.8332 | AETGQETAY |
| **Pol** | **807** | **815** | **7** | **22.3022** | **ETGQETAYF** |
| Pol | 811 | 819 | 1 | 2.5897 | ETAYFLLKL |
| **Pol** | **833** | **841** | **15** | **53.2913** | **GSNFTGATV** |
| Pol | 842 | 850 | 1 | 11.0205 | RAACWWAGI |
| Pol | 872 | 880 | 1 | 0.1788 | ELKKIIGQV |
| Pol | 879 | 887 | 1 | 11.0205 | QVRDQAEHL |
| **Pol** | **888** | **896** | **20** | **84.883** | **KTAVQMAVF** |
| Pol | 892 | 900 | 5 | 12.7208 | QMAVFIHNF |
| **Pol** | **893** | **901** | **4** | **30.272** | **MAVFIHNFK** |
| Pol | 901 | 909 | 1 | 0.6556 | KRKGGIGGY |
| Pol | 920 | 928 | 4 | 2.8952 | ATDIQTKEL |
| **Pol** | **926** | **934** | **7** | **22.9525** | **KELQKQITK** |
| **Pol** | **930** | **938** | **19** | **46.3082** | **KQITKIQNF** |
| Pol | 932 | 940 | 2 | 1.9585 | ITKIQNFRV |
| Pol | 941 | 949 | 1 | 1.2485 | YYRDSRNPL |
| Pol | 947 | 955 | 9 | 19.8751 | NPLWKGPAK |
| **Pol** | **955** | **963** | **7** | **40.9998** | **KLLWKGEGA** |
| **Pol** | **956** | **964** | **7** | **40.1723** | **LLWKGEGAV** |
| Pol | 966 | 974 | 1 | 1.2485 | IQDNSDIKV |
| Pol | 982 | 990 | 1 | 11.0205 | IIRDYGKQM |
| **Pol** | **988** | **996** | **23** | **65.9362** | **KQMAGDDCV** |
| **Rev** | **4** | **12** | **28** | **87.5172** | **RSGDSDEEL** |
| **Rev** | **10** | **18** | **10** | **60.5994** | **EELIRTVRL** |
| **Rev** | **14** | **22** | **6** | **28.0844** | **RTVRLIKLL** |
| Rev | 15 | 23 | 1 | 0.6556 | TVRLIKLLY |
| Rev | 30 | 38 | 7 | 18.6198 | NPEGTRQAR |
| **Rev** | **35** | **43** | **5** | **34.6821** | **RQARRNRRR** |
| Rev | 44 | 52 | 2 | 1.4618 | RWRERQRQI |
| **Rev** | **47** | **55** | **5** | **25.0245** | **ERQRQIHSI** |
| **Rev** | **50** | **58** | **13** | **49.9404** | **RQIHSISER** |
| **Rev** | **55** | **63** | **15** | **52.7936** | **ISERILGTY** |
| Rev | 56 | 64 | 12 | 31.264 | SERILGTYL |
| **Rev** | **63** | **71** | **9** | **43.0433** | **YLGRSAEPV** |
| Rev | 69 | 77 | 2 | 2.8449 | EPVPLQLPP |
| **Rev** | **75** | **83** | **6** | **37.3585** | **LPPLERLTL** |
| **Rev** | **94** | **102** | **12** | **68.2611** | **TQGVGSPQI** |
| Rev | 96 | 104 | 3 | 6.476 | GVGSPQILV |
| **Rev** | **101** | **109** | **13** | **44.9517** | **QILVESPTV** |
| **Rev** | **102** | **110** | **5** | **35.4065** | **ILVESPTVL** |
| Tat | 20 | 28 | 1 | 5.2957 | TACTNCYCK |
| Tat | 28 | 36 | 2 | 5.2608 | KKCCFHCQV |
| **Tat** | **39** | **47** | **30** | **86.9814** | **ITKALGISY** |
| **Tat** | **59** | **67** | **31** | **90.9852** | **HQNSQTHQA** |
| Tat | 61 | 69 | 1 | 11.0205 | NSQTHQASL |
| **Tat** | **63** | **71** | **12** | **46.1071** | **QTHQASLSK** |
| **Vif** | **1** | **9** | **7** | **26.4307** | **MENRWQVMI** |
| **Vif** | **5** | **13** | **14** | **44.3639** | **WQVMIVWQV** |
| Vif | 17 | 25 | 1 | 11.0205 | RIRTWKSLV |
| Vif | 22 | 30 | 6 | 31.264 | KSLVKHHMY |
| **Vif** | **23** | **31** | **8** | **41.1371** | **SLVKHHMYV** |
| Vif | 30 | 38 | 1 | 0.7144 | YVSGKARGW |
| Vif | 36 | 44 | 4 | 2.8143 | RGWFYRHHY |
| Vif | 43 | 51 | 2 | 0.4468 | HYESPHPRI |
| **Vif** | **51** | **59** | **4** | **34.6526** | **ISSEVHIPL** |
| Vif | 61 | 69 | 5 | 18.0463 | DARLVITTY |
| **Vif** | **73** | **81** | **21** | **78.4669** | **HTGERDWHL** |
| Vif | 77 | 85 | 2 | 5.2608 | RDWHLGQGV |
| **Vif** | **102** | **110** | **7** | **28.6623** | **LADQLIHLY** |
| **Vif** | **127** | **135** | **14** | **51.4706** | **HIVSPRCEY** |
| **Vif** | **133** | **141** | **10** | **24.5702** | **CEYQAGHNK** |
| Vif | 144 | 152 | 1 | 1.2485 | SLQYLALAA |
| **Vif** | **145** | **153** | **14** | **70.4782** | **LQYLALAAL** |
| Vif | 149 | 157 | 2 | 3.4184 | ALAALITPK |
| Vif | 155 | 163 | 2 | 11.6389 | TPKKIKPPL |
| **Vif** | **158** | **166** | **7** | **37.5422** | **KIKPPLPSV** |
| **Vif** | **160** | **168** | **9** | **23.8422** | **KPPLPSVTK** |
| Vif | 166 | 174 | 1 | 0.6556 | VTKLTEDRW |
| **Vif** | **168** | **176** | **4** | **31.9058** | **KLTEDRWNK** |
| **Vif** | **173** | **181** | **8** | **74.7399** | **RWNKPQKTK** |
| Vpr | 4 | 12 | 5 | 18.0828 | APEDQGPQR |
| **Vpr** | **12** | **20** | **10** | **40.3144** | **REPHNEWTL** |
| Vpr | 23 | 31 | 7 | 14.0907 | LEELKNEAV |
| Vpr | 28 | 36 | 3 | 5.9165 | NEAVRHFPR |
| **Vpr** | **34** | **42** | **21** | **81.3389** | **FPRIWLHGL** |
| **Vpr** | **38** | **46** | **10** | **43.5747** | **WLHGLGQHI** |
| **Vpr** | **46** | **54** | **5** | **55.8057** | **IYETYGDTW** |
| **Vpr** | **52** | **60** | **4** | **23.4902** | **DTWAGVEAI** |
| **Vpr** | **64** | **72** | **6** | **21.6542** | **LQQLLFIHF** |
| Vpr | 65 | 73 | 4 | 30.272 | QQLLFIHFR |
| Vpr | 85 | 93 | 2 | 1.8026 | RQRRARNGA |
| **Vpu** | **4** | **12** | **22** | **73.9292** | **IPIVAIVAL** |
| Vpu | 13 | 21 | 3 | 5.4349 | VVAIIIAIV |
| **Vpu** | **17** | **25** | **12** | **54.6154** | **IIAIVVWSI** |
| Vpu | 22 | 30 | 1 | 0.2146 | VWSIVIIEY |
| Vpu | 29 | 37 | 3 | 29.823 | EYRKILRQR |
| Vpu | 35 | 43 | 2 | 12.2737 | RQRKIDRLI |
| **Vpu** | **38** | **46** | **6** | **56.6344** | **KIDRLIDRL** |
| Vpu | 41 | 49 | 4 | 6.8045 | RLIDRLIER |
| **Vpu** | **69** | **77** | **24** | **70.2441** | **VEMGHHAPW** |

**Table S8.** Full list of HIV-1 specific CD4+ T-cell epitopes predicted by Predivac-3.0 for the Japanese population, using Epitope Discovery mode (PPR=1; PCT=0). Reactive T-cell epitopes are highlighted in bold for those predicted to cover ≥ 20% (black) and ≥ 80% (red) of the Japanese population.

| **Protein** | **Start** | **End** | **N° of HLA**  **class II alleles** | **Population**  **Coverage (%)** | **Putative CD4+**  **T-cell epitopes** |
| --- | --- | --- | --- | --- | --- |
| Env | 24 | 32 | 3 | 3.5286 | MLMICSATE |
| Env | 26 | 34 | 1 | 5.5795 | MICSATEKL |
| Env | 45 | 53 | 1 | 0.3859 | WKEATTTLF |
| Env | 54 | 62 | 3 | 6.8481 | CASDAKAYD |
| **Env** | **61** | **69** | **5** | **20.9545** | **YDTEVHNVW** |
| **Env** | **75** | **83** | **17** | **55.0315** | **VPTDPNPQE** |
| Env | 89 | 97 | 2 | 3.8912 | VTENFNMWK |
| **Env** | **93** | **101** | **10** | **36.7261** | **FNMWKNDMV** |
| **Env** | **96** | **104** | **4** | **24.0882** | **WKNDMVEQM** |
| Env | 101 | 109 | 4 | 12.8429 | VEQMHEDII |
| **Env** | **112** | **120** | **4** | **31.3672** | **WDQSLKPCV** |
| Env | 116 | 124 | 1 | 1.922 | LKPCVKLTP |
| Env | 125 | 133 | 3 | 6.8481 | LCVSLKCTD |
| Env | 127 | 135 | 1 | 5.5795 | VSLKCTDLK |
| **Env** | **134** | **142** | **16** | **50.0467** | **LKNDTNTNS** |
| Env | 147 | 155 | 1 | 0.1511 | MIMEKGEIK |
| **Env** | **159** | **167** | **6** | **29.0099** | **FNISTSIRG** |
| Env | 173 | 181 | 4 | 12.9213 | YAFFYKLDI |
| **Env** | **177** | **185** | **8** | **27.0295** | **YKLDIIPID** |
| **Env** | **181** | **189** | **9** | **35.4102** | **IIPIDNDTT** |
| **Env** | **182** | **190** | **6** | **25.7616** | **IPIDNDTTS** |
| Env | 184 | 192 | 6 | 3.8912 | IDNDTTSYK |
| Env | 191 | 199 | 2 | 1.8222 | YKLTSCNTS |
| Env | 193 | 201 | 1 | 5.5795 | LTSCNTSVI |
| **Env** | **200** | **208** | **1** | **22.0852** | **VITQACPKV** |
| **Env** | **210** | **218** | **1** | **22.0852** | **FEPIPIHYC** |
| Env | 223 | 231 | 4 | 11.4735 | FAILKCNNK |
| **Env** | **233** | **241** | **2** | **22.4258** | **FNGTGPCTN** |
| Env | 275 | 283 | 2 | 10.2686 | VNFTDNAKT |
| **Env** | **277** | **285** | **14** | **29.1796** | **FTDNAKTII** |
| Env | 284 | 292 | 1 | 0 | IIVQLNTSV |
| Env | 285 | 293 | 3 | 3.5286 | IVQLNTSVE |
| Env | 286 | 294 | 2 | 9.1355 | VQLNTSVEI |
| Env | 294 | 302 | 1 | 0.9717 | INCTRPNNN |
| **Env** | **317** | **325** | **16** | **29.1372** | **FVTIGKIGN** |
| **Env** | **320** | **328** | **21** | **41.0448** | **IGKIGNMRQ** |
| **Env** | **338** | **346** | **5** | **20.9545** | **WNNTLKQIA** |
| Env | 349 | 357 | 1 | 0.9717 | LREQFGNNK |
| Env | 372 | 380 | 1 | 4.8274 | VTHSFNCGG |
| **Env** | **382** | **390** | **6** | **36.5523** | **FFYCNSTQL** |
| Env | 396 | 404 | 2 | 5.6611 | FNSTWSTEG |
| **Env** | **400** | **408** | **24** | **72.6682** | **WSTEGSNNT** |
| **Env** | **420** | **428** | **21** | **41.0448** | **IKQIINMWQ** |
| Env | 424 | 432 | 2 | 3.8912 | INMWQKVGK |
| Env | 426 | 434 | 1 | 22.0852 | MWQKVGKAM |
| Env | 427 | 435 | 3 | 10.5547 | WQKVGKAMY |
| Env | 435 | 443 | 1 | 0.3859 | YAPPISGQI |
| Env | 439 | 447 | 5 | 4.3681 | ISGQIRCSS |
| Env | 443 | 451 | 1 | 4.8274 | IRCSSNITG |
| **Env** | **454** | **462** | **9** | **31.6452** | **LTRDGGNSN** |
| Env | 475 | 483 | 5 | 12.984 | MRDNWRSEL |
| **Env** | **488** | **496** | **2** | **22.0852** | **VVKIEPLGV** |
| Env | 494 | 502 | 1 | 5.5795 | LGVAPTKAK |
| **Env** | **506** | **514** | **23** | **60.8233** | **VQREKRAVG** |
| **Env** | **519** | **527** | **3** | **26.0507** | **FLGFLGAAG** |
| Env | 522 | 530 | 2 | 9.9023 | FLGAAGSTM |
| **Env** | **535** | **543** | **1** | **22.0852** | **MTLTVQARQ** |
| Env | 537 | 545 | 2 | 9.1355 | LTVQARQLL |
| Env | 548 | 556 | 2 | 9.1355 | IVQQQNNLL |
| Env | 549 | 557 | 3 | 9.3595 | VQQQNNLLR |
| Env | 556 | 564 | 1 | 0.9717 | LRAIEAQQH |
| Env | 573 | 581 | 1 | 0 | IKQLQARIL |
| **Env** | **586** | **594** | **11** | **43.7846** | **YLKDQQLLG** |
| Env | 595 | 603 | 1 | 0.3859 | IWGCSGKLI |
| **Env** | **602** | **610** | **1** | **22.0852** | **LICTTAVPW** |
| **Env** | **610** | **618** | **17** | **39.8004** | **WNASWSNKS** |
| Env | 619 | 627 | 6 | 27.4735 | LEQIWNHTT |
| Env | 628 | 636 | 6 | 4.9747 | WMEWDREIN |
| **Env** | **629** | **637** | **6** | **31.3394** | **MEWDREINN** |
| **Env** | **631** | **639** | **37** | **81.3498** | **WDREINNYT** |
| Env | 638 | 646 | 1 | 0.3859 | YTSLIHSLI |
| Env | 645 | 653 | 1 | 1.922 | LIEESQNQQ |
| Env | 646 | 654 | 2 | 5.6611 | IEESQNQQE |
| Env | 666 | 674 | 5 | 14.1082 | WASLWNWFN |
| Env | 678 | 686 | 1 | 2.5032 | WLWYIKLFI |
| **Env** | **685** | **693** | **4** | **25.1962** | **FIMIVGGLV** |
| Env | 689 | 697 | 3 | 9.1355 | VGGLVGLRI |
| Env | 695 | 703 | 1 | 0.9717 | LRIVFAVLS |
| Env | 701 | 709 | 4 | 9.5033 | VLSIVNRVR |
| Env | 733 | 741 | 3 | 8.3333 | IEEEGGERD |
| **Env** | **756** | **764** | **9** | **31.6452** | **IWDDLRSLC** |
| **Env** | **757** | **765** | **8** | **53.0161** | **WDDLRSLCL** |
| Env | 768 | 776 | 3 | 1.6557 | YHRLRDLLL |
| Env | 778 | 786 | 7 | 13.297 | VTRIVELLG |
| Env | 781 | 789 | 2 | 9.5353 | IVELLGRRG |
| Env | 793 | 801 | 5 | 4.3681 | LKYWWNLLQ |
| Env | 799 | 807 | 2 | 10.8404 | LLQYWSQEL |
| **Env** | **803** | **811** | **29** | **61.0332** | **WSQELKNSA** |
| Env | 812 | 820 | 2 | 9.5353 | VSLLNATAI |
| Env | 833 | 841 | 1 | 0.1511 | VQGACRAIR |
| **Env** | **840** | **848** | **12** | **20.6705** | **IRHIPRRIR** |
| Gag | 13 | 21 | 2 | 9.1355 | LDRWEKIRL |
| **Gag** | **34** | **42** | **7** | **22.7951** | **IVWASRELE** |
| **Gag** | **35** | **43** | **6** | **25.7616** | **VWASRELER** |
| **Gag** | **42** | **50** | **1** | **22.0852** | **ERFAVNPGL** |
| **Gag** | **44** | **52** | **20** | **59.2628** | **FAVNPGLLE** |
| Gag | 79 | 87 | 1 | 0.8547 | YNTVATLYC |
| Gag | 86 | 94 | 5 | 3.248 | YCVHQRIEI |
| Gag | 93 | 101 | 3 | 0.3691 | EIKDTKEAL |
| Gag | 104 | 112 | 4 | 0.5199 | IEEEQNKSK |
| **Gag** | **128** | **136** | **3** | **25.4429** | **VSQNYPIVQ** |
| **Gag** | **132** | **140** | **23** | **65.893** | **YPIVQNIQG** |
| Gag | 135 | 143 | 4 | 19.8307 | VQNIQGQMV |
| Gag | 143 | 151 | 5 | 11.8366 | VHQAISPRT |
| Gag | 168 | 176 | 11 | 11.5525 | VIPMFSALS |
| Gag | 171 | 179 | 9 | 15.935 | MFSALSEGA |
| Gag | 184 | 192 | 8 | 13.297 | LNTMLNTVG |
| **Gag** | **200** | **208** | **5** | **31.0189** | **MLKETINEE** |
| Gag | 201 | 209 | 2 | 5.6611 | LKETINEEA |
| **Gag** | **205** | **213** | **7** | **32.9304** | **INEEAAEWD** |
| **Gag** | **212** | **220** | **4** | **31.6035** | **WDRVHPVHA** |
| Gag | 215 | 223 | 1 | 0.3859 | VHPVHAGPI |
| Gag | 236 | 244 | 1 | 4.8274 | IAGTTSTLQ |
| Gag | 247 | 255 | 6 | 5.3182 | IGWMTNNPP |
| **Gag** | **249** | **257** | **2** | **22.4258** | **WMTNNPPIP** |
| Gag | 258 | 266 | 5 | 12.984 | VGEIYKRWI |
| Gag | 267 | 275 | 6 | 14.3726 | ILGLNKIVR |
| **Gag** | **270** | **278** | **17** | **24.4536** | **LNKIVRMYS** |
| **Gag** | **273** | **281** | **28** | **65.2723** | **IVRMYSPTS** |
| Gag | 277 | 285 | 1 | 0.3859 | YSPTSILDI |
| Gag | 285 | 293 | 2 | 9.1355 | IRQGPKEPF |
| **Gag** | **300** | **308** | **5** | **20.9545** | **FYKTLRAEQ** |
| **Gag** | **301** | **309** | **15** | **50.3548** | **YKTLRAEQA** |
| Gag | 304 | 312 | 2 | 6.4749 | LRAEQASQE |
| **Gag** | **316** | **324** | **5** | **20.9545** | **WMTETLLVQ** |
| Gag | 321 | 329 | 1 | 0.9717 | LLVQNANPD |
| Gag | 322 | 330 | 3 | 6.8481 | LVQNANPDC |
| **Gag** | **333** | **341** | **1** | **22.0852** | **ILKALGPAA** |
| Gag | 334 | 342 | 2 | 9.5353 | LKALGPAAT |
| **Gag** | **337** | **345** | **10** | **36.5657** | **LGPAATLEE** |
| **Gag** | **343** | **351** | **7** | **27.1585** | **LEEMMTACQ** |
| Gag | 347 | 355 | 3 | 12.6547 | MTACQGVGG |
| Gag | 363 | 371 | 1 | 0.3859 | LAEAMSQVT |
| **Gag** | **367** | **375** | **3** | **26.0507** | **MSQVTNSAT** |
| Gag | 370 | 378 | 1 | 5.5795 | VTNSATIMM |
| Gag | 376 | 384 | 1 | 0.9717 | IMMQRGNFR |
| Gag | 383 | 391 | 4 | 12.9213 | FRNQRKIVK |
| Gag | 389 | 397 | 3 | 8.1564 | IVKCFNCGK |
| Gag | 392 | 400 | 1 | 2.5032 | CFNCGKEGH |
| Gag | 413 | 421 | 1 | 2.5032 | CWKCGKEGH |
| Gag | 423 | 431 | 1 | 1.922 | MKDCTERQA |
| Gag | 438 | 446 | 1 | 16.1504 | WPSYKGRPG |
| Gag | 448 | 456 | 4 | 0.7543 | FLQSRPEPT |
| **Gag** | **466** | **474** | **1** | **22.0852** | **GVETTTPPQ** |
| Gag | 483 | 491 | 2 | 10.8404 | LYPLTSLRS |
| Gag | 486 | 494 | 2 | 9.1355 | LTSLRSLFG |
| Gag | 489 | 497 | 1 | 0.9717 | LRSLFGNDP |
| Nef | 10 | 18 | 4 | 8.269 | VIGWPTVRE |
| Nef | 30 | 38 | 1 | 1.922 | VGAASRDLE |
| Nef | 43 | 51 | 2 | 10.2686 | ITSSNTAAT |
| **Nef** | **68** | **76** | **1** | **22.0852** | **FPVTPQVPL** |
| **Nef** | **79** | **87** | **1** | **22.0852** | **MTYKAAVDL** |
| **Nef** | **81** | **89** | **6** | **29.6593** | **YKAAVDLSH** |
| Nef | 87 | 95 | 2 | 10.8404 | LSHFLKEKG |
| **Nef** | **90** | **98** | **9** | **35.2616** | **FLKEKGGLE** |
| Nef | 100 | 108 | 3 | 6.8481 | LIHSQRRQD |
| Nef | 101 | 109 | 2 | 9.1355 | IHSQRRQDI |
| Nef | 120 | 128 | 6 | 2.0218 | YFPDWQNYT |
| Nef | 121 | 129 | 1 | 16.1504 | FPDWQNYTP |
| Nef | 127 | 135 | 1 | 0.9717 | YTPGPGVRY |
| Nef | 135 | 143 | 1 | 0.3859 | YPLTFGWCY |
| **Nef** | **139** | **147** | **12** | **27.0295** | **FGWCYKLVP** |
| Nef | 143 | 151 | 1 | 0.8547 | YKLVPVEPD |
| Nef | 145 | 153 | 3 | 9.9023 | LVPVEPDKI |
| **Nef** | **148** | **156** | **3** | **25.4429** | **VEPDKIEEA** |
| Nef | 151 | 159 | 1 | 0.9717 | DKIEEANKG |
| Nef | 170 | 178 | 9 | 18.5474 | LHGMDDPER |
| Nef | 182 | 190 | 1 | 16.1504 | EWRFDSRLA |
| **Nef** | **183** | **191** | **19** | **50.4258** | **WRFDSRLAF** |
| Nef | 191 | 199 | 22 | 42.9505 | FHHVARELH |
| Nef | 194 | 202 | 1 | 0.1511 | VARELHPEY |
| **Pol** | **2** | **10** | **7** | **46.0159** | **FREDLAFLQ** |
| Pol | 6 | 14 | 1 | 0.9717 | LAFLQGKAR |
| Pol | 9 | 17 | 1 | 1.922 | LQGKAREFS |
| **Pol** | **16** | **24** | **14** | **45.7812** | **FSSEQTRAN** |
| **Pol** | **34** | **42** | **33** | **81.574** | **WGRDNNSPS** |
| Pol | 59 | 67 | 4 | 12.8429 | VTLWQRPLV |
| **Pol** | **88** | **96** | **17** | **48.5159** | **VLEEMSLPG** |
| **Pol** | **119** | **127** | **6** | **51.8696** | **LIEICGHKA** |
| Pol | 120 | 128 | 1 | 0.3859 | IEICGHKAI |
| Pol | 131 | 139 | 1 | 4.8274 | VLVGPTPVN |
| Pol | 133 | 141 | 1 | 5.5795 | VGPTPVNII |
| Pol | 138 | 146 | 5 | 12.984 | VNIIGRNLL |
| Pol | 149 | 157 | 2 | 5.9545 | IGCTLNFPI |
| **Pol** | **157** | **165** | **5** | **39.4612** | **ISPIETVPV** |
| Pol | 163 | 171 | 1 | 0.1511 | VPVKLKPGM |
| **Pol** | **189** | **197** | **8** | **31.2698** | **LVEICTEME** |
| Pol | 190 | 198 | 1 | 0.1511 | VEICTEMEK |
| Pol | 192 | 200 | 1 | 1.922 | ICTEMEKEG |
| Pol | 196 | 204 | 2 | 2.6524 | MEKEGKISK |
| Pol | 205 | 213 | 2 | 9.5353 | IGPENPYNT |
| Pol | 215 | 223 | 6 | 6.2474 | VFAIKKKDS |
| **Pol** | **216** | **224** | **6** | **23.1783** | **FAIKKKDST** |
| **Pol** | **232** | **240** | **20** | **39.4873** | **FRELNKRTQ** |
| **Pol** | **245** | **253** | **2** | **22.0852** | **VQLGIPHPA** |
| **Pol** | **247** | **255** | **1** | **22.0852** | **LGIPHPAGL** |
| **Pol** | **275** | **283** | **9** | **31.6452** | **LDEDFRKYT** |
| Pol | 279 | 287 | 2 | 16.5038 | FRKYTAFTI |
| Pol | 282 | 290 | 1 | 16.1504 | YTAFTIPSI |
| Pol | 308 | 316 | 1 | 0.8547 | WKGSPAIFQ |
| **Pol** | **315** | **323** | **2** | **22.4258** | **FQSSMTKIL** |
| Pol | 319 | 327 | 2 | 3.8912 | MTKILEPFR |
| Pol | 326 | 334 | 3 | 9.9023 | FRKQNPDIV |
| **Pol** | **336** | **344** | **10** | **43.9733** | **YQYMDDLYV** |
| Pol | 338 | 346 | 1 | 0.3859 | YMDDLYVGS |
| **Pol** | **344** | **352** | **6** | **25.7616** | **VGSDLEIGQ** |
| Pol | 385 | 393 | 1 | 0.1511 | MGYELHPDK |
| Pol | 401 | 409 | 1 | 0.3859 | LPEKDSWTV |
| **Pol** | **409** | **417** | **17** | **23.2813** | **VNDIQKLVG** |
| Pol | 412 | 420 | 1 | 0.9717 | IQKLVGKLN |
| Pol | 431 | 439 | 11 | 18.411 | VRQLCKLLR |
| Pol | 438 | 446 | 1 | 4.8274 | LRGTKALTE |
| Pol | 444 | 452 | 1 | 0.9717 | LTEVIPLTE |
| Pol | 456 | 464 | 2 | 5.9545 | LELAENREI |
| Pol | 474 | 482 | 1 | 0.8547 | YDPSKDLIA |
| **Pol** | **481** | **489** | **19** | **33.9852** | **IAEIQKQGQ** |
| Pol | 498 | 506 | 2 | 10.8404 | QEPFKNLKT |
| **Pol** | **509** | **517** | **15** | **39.3043** | **YARMRGAHT** |
| Pol | 512 | 520 | 1 | 5.5795 | MRGAHTNDV |
| **Pol** | **527** | **535** | **5** | **24.4536** | **VQKITTESI** |
| Pol | 556 | 564 | 3 | 1.6557 | WWTEYWQAT |
| **Pol** | **561** | **569** | **1** | **22.0852** | **WQATWIPEW** |
| **Pol** | **569** | **577** | **3** | **40.6962** | **WEFVNTPPL** |
| **Pol** | **581** | **589** | **14** | **29.1796** | **WYQLEKEPI** |
| Pol | 582 | 590 | 2 | 2.8843 | YQLEKEPIV |
| **Pol** | **595** | **603** | **13** | **47.4987** | **FYVDGAANR** |
| Pol | 596 | 604 | 2 | 1.8222 | YVDGAANRE |
| Pol | 597 | 605 | 2 | 10.2686 | VDGAANRET |
| Pol | 612 | 620 | 3 | 10.5547 | YVTNRGRQK |
| Pol | 622 | 630 | 2 | 5.6611 | VTLTDTTNQ |
| Pol | 624 | 632 | 2 | 10.2686 | LTDTTNQKT |
| Pol | 638 | 646 | 1 | 16.1504 | YLALQDSGL |
| Pol | 658 | 666 | 1 | 0.9717 | LGIIQAQPD |
| Pol | 673 | 681 | 2 | 9.1355 | VNQIIEQLI |
| Pol | 677 | 685 | 2 | 3.8912 | IEQLIKKEK |
| Pol | 686 | 694 | 1 | 5.5795 | VYLAWVPAH |
| **Pol** | **690** | **698** | **5** | **20.9545** | **WVPAHKGIG** |
| Pol | 703 | 711 | 3 | 10.8404 | VDKLVSAGI |
| **Pol** | **715** | **723** | **3** | **25.4429** | **LFLDGIDKA** |
| Pol | 717 | 725 | 6 | 6.2474 | LDGIDKAQD |
| **Pol** | **730** | **738** | **21** | **40.2426** | **YHSNWRAMA** |
| **Pol** | **734** | **742** | **16** | **51.0388** | **WRAMASDFN** |
| **Pol** | **737** | **745** | **12** | **34.5571** | **MASDFNLPP** |
| Pol | 752 | 760 | 1 | 0.1511 | VASCDKCQL |
| **Pol** | **776** | **784** | **21** | **67.5878** | **WQLDCTHLE** |
| Pol | 798 | 806 | 1 | 0.8547 | YIEAEVIPA |
| Pol | 803 | 811 | 1 | 4.8274 | VIPAETGQE |
| Pol | 804 | 812 | 6 | 12.0257 | IPAETGQET |
| **Pol** | **825** | **833** | **14** | **51.8463** | **VKTIHTDNG** |
| Pol | 836 | 844 | 1 | 0.8547 | FTGATVRAA |
| **Pol** | **865** | **873** | **1** | **22.0852** | **VVESMNKEL** |
| Pol | 866 | 874 | 10 | 18.2744 | VESMNKELK |
| Pol | 869 | 877 | 6 | 17.7422 | MNKELKKII |
| Pol | 873 | 881 | 1 | 0.9717 | LKKIIGQVR |
| Pol | 876 | 884 | 5 | 4.3681 | IIGQVRDQA |
| Pol | 896 | 904 | 28 | 55.9057 | FIHNFKRKG |
| Pol | 909 | 917 | 6 | 4.9747 | YSAGERIVD |
| **Pol** | **915** | **923** | **5** | **20.1762** | **IVDIIATDI** |
| Pol | 916 | 924 | 9 | 31.978 | VDIIATDIQ |
| Pol | 919 | 927 | 2 | 6.4749 | IATDIQTKE |
| **Pol** | **932** | **940** | **3** | **26.0507** | **ITKIQNFRV** |
| Pol | 938 | 946 | 1 | 2.5032 | FRVYYRDSR |
| **Pol** | **941** | **949** | **22** | **59.0482** | **YYRDSRNPL** |
| **Pol** | **942** | **950** | **2** | **22.8394** | **YRDSRNPLW** |
| **Pol** | **958** | **966** | **8** | **32.9716** | **WKGEGAVVI** |
| Pol | 964 | 972 | 2 | 9.1355 | VVIQDNSDI |
| Pol | 965 | 973 | 6 | 3.8912 | VIQDNSDIK |
| Pol | 982 | 990 | 3 | 0.3691 | IIRDYGKQM |
| **Pol** | **990** | **998** | **12** | **34.5571** | **MAGDDCVAS** |
| Rev | 6 | 14 | 3 | 0.3691 | GDSDEELIR |
| Rev | 21 | 29 | 1 | 0.8547 | LLYQSNPPP |
| **Rev** | **22** | **30** | **3** | **27.7878** | **LYQSNPPPN** |
| **Rev** | **23** | **31** | **16** | **52.3345** | **YQSNPPPNP** |
| **Rev** | **45** | **53** | **14** | **29.2926** | **WRERQRQIH** |
| Rev | 52 | 60 | 4 | 12.8429 | IHSISERIL |
| **Rev** | **60** | **68** | **3** | **26.0507** | **LGTYLGRSA** |
| **Rev** | **63** | **71** | **3** | **25.1962** | **YLGRSAEPV** |
| Rev | 71 | 79 | 7 | 9.0875 | VPLQLPPLE |
| **Rev** | **75** | **83** | **14** | **28.7834** | **LPPLERLTL** |
| Rev | 80 | 88 | 1 | 0.9717 | RLTLDCNED |
| **Rev** | **81** | **89** | **22** | **63.2138** | **LTLDCNEDC** |
| **Rev** | **85** | **93** | **6** | **26.8716** | **CNEDCGTSG** |
| Rev | 89 | 97 | 1 | 5.5795 | CGTSGTQGV |
| Rev | 102 | 110 | 3 | 9.9023 | ILVESPTVL |
| **Rev** | **103** | **111** | **11** | **37.3391** | **LVESPTVLE** |
| Tat | 1 | 9 | 2 | 4.8274 | MEPVDPRLE |
| **Tat** | **2** | **10** | **6** | **31.3394** | **EPVDPRLEP** |
| **Tat** | **8** | **16** | **3** | **26.0507** | **LEPWKHPGS** |
| Tat | 11 | 19 | 1 | 0.9717 | WKHPGSQPK |
| Tat | 32 | 40 | 4 | 11.1097 | FHCQVCFIT |
| **Tat** | **36** | **44** | **17** | **23.2813** | **VCFITKALG** |
| Tat | 37 | 45 | 1 | 0.3859 | CFITKALGI |
| Tat | 39 | 47 | 1 | 5.5795 | ITKALGISY |
| **Tat** | **47** | **55** | **12** | **32.6964** | **YGRKKRRQR** |
| **Tat** | **59** | **67** | **1** | **22.0852** | **HQNSQTHQA** |
| **Tat** | **69** | **77** | **8** | **27.8877** | **LSKQPTSQP** |
| Vif | 10 | 18 | 2 | 9.1355 | VWQVDRMRI |
| Vif | 11 | 19 | 3 | 0.3691 | WQVDRMRIR |
| Vif | 18 | 26 | 1 | 5.5795 | IRTWKSLVK |
| Vif | 21 | 29 | 19 | 56.528 | WKSLVKHHM |
| **Vif** | **29** | **37** | **13** | **42.2762** | **MYVSGKARG** |
| Vif | 58 | 66 | 2 | 6.4749 | PLGDARLVI |
| **Vif** | **69** | **77** | **4** | **24.0882** | **YWGLHTGER** |
| Vif | 70 | 78 | 1 | 0.3859 | WGLHTGERD |
| Vif | 72 | 80 | 7 | 8.1725 | LHTGERDWH |
| Vif | 79 | 87 | 2 | 9.9023 | WHLGQGVSI |
| Vif | 85 | 93 | 8 | 27.545 | VSIEWRKKR |
| Vif | 98 | 106 | 1 | 0 | VDPELADQL |
| **Vif** | **111** | **119** | **13** | **51.5428** | **YFDCFSDSA** |
| **Vif** | **112** | **120** | **3** | **26.0507** | **FDCFSDSAI** |
| **Vif** | **125** | **133** | **5** | **29.3632** | **LGHIVSPRC** |
| **Vif** | **135** | **143** | **15** | **31.3394** | **YQAGHNKVG** |
| **Vif** | **147** | **155** | **9** | **21.9072** | **YLALAALIT** |
| Vif | 150 | 158 | 4 | 9.0074 | LAALITPKK |
| Vpr | 1 | 9 | 3 | 3.5286 | MEQAPEDQG |
| **Vpr** | **22** | **30** | **19** | **58.2599** | **LLEELKNEA** |
| Vpr | 23 | 31 | 6 | 12.4034 | LEELKNEAV |
| Vpr | 34 | 42 | 1 | 22.0852 | FPRIWLHGL |
| Vpr | 38 | 46 | 3 | 10.8245 | WLHGLGQHI |
| Vpr | 47 | 55 | 1 | 16.1504 | YETYGDTWA |
| **Vpr** | **50** | **58** | **5** | **24.8326** | **YGDTWAGVE** |
| Vpr | 54 | 62 | 1 | 5.5795 | WAGVEAIIR |
| Vpr | 57 | 65 | 5 | 4.3681 | VEAIIRILQ |
| Vpr | 72 | 80 | 11 | 18.8352 | FRIGCRHSR |
| Vpr | 74 | 82 | 1 | 4.8274 | IGCRHSRIG |
| **Vpu** | **6** | **14** | **3** | **26.0507** | **IVAIVALVV** |
| Vpu | 16 | 24 | 3 | 11.0781 | IIIAIVVWS |
| Vpu | 26 | 34 | 4 | 0.5199 | VIIEYRKIL |
| **Vpu** | **30** | **38** | **17** | **39.5657** | **YRKILRQRK** |
| Vpu | 33 | 41 | 5 | 4.3681 | ILRQRKIDR |
| Vpu | 39 | 47 | 2 | 9.1355 | IDRLIDRLI |
| **Vpu** | **67** | **75** | **21** | **75.688** | **MGVEMGHHA** |
| Vpu | 71 | 79 | 1 | 0.3859 | MGHHAPWDV |

**Table S9**. T-cell epitope clusters and hotspots predicted by Predivac-3.0 in the HIV-1 proteome (HXB2 isolate), reporting the position in each protein. Putative T-cell epitopes associated with the clusters and population coverage potentially afforded by the epitope sets in the Japanese population. The overlapped regions are presented in bold and underlines for each epitope sequence. Overlapped CD8+ and CD4+ epitope clusters are highlighted in grey color.

| **Prot** | **Cluster** | | | **T-cell epitopes** | |
| --- | --- | --- | --- | --- | --- |
|  | **Sequence** | **Position**  **(start-end)** | **Population**  **coverage (%)** | **CD8+ T-cell epitopes** | **CD4+ T-cell epitopes** |
| Env | VTENMWKNDMVEQMHEDII | 91-109  (94-104) | 0.640 | - | VTENFNMWK;FNMWKNDMV;WKNDMVEQM;VEQMHEDII |
| Env | IISLWDQSLKPCVKLTPLCVSL | 108-129  (114-123) | 0.926 | IISLWDQSL;KPCVKLTPL;CVKLTPLCV;KLTPLCVSL | - |
| Env | NTNSSSGRMIMEKGEIKNCSFNISTSI | 139-165  (144-158) | 0.708 | NTNSSSGRM;SSGRMIMEK;MIMEKGEIK;KGEIKNCSF;CSFNISTSI | - |
| Env | YAFFYKLDIIPIDNDTTSYKLTSCNTSVI | 173-201  (175-197) | 0.819 | - | YAFFYKLDI;YKLDIIPID;IIPIDNDTT;IPIDNDTTS;IDNDTTSYK;YKLTSCNTS;LTSCNTSVI; |
| Env | SVITQACPKVSFEPIPIHYCAPAGFAILKCNNKTF | 199-233  (204-228) | 0.885 | SVITQACPK;CPKVSFEPI;KVSFEPIPI;EPIPIHYCA;IPIHYCAPA;APAGFAILK;FAILKCNNK;ILKCNNKTF | - |
| Env | STVQCTHGIRPVVSTQLLLNGSL | 243-265  (250-258) | 0.920 | STVQCTHGI;CTHGIRPV;  RPVVSTQLL;TQLLLNGSL | - |
| Env | VNFTDNAKTIIVQLNTSVEI | 275-294  (278-292) | 0.406 | - | VNFTDNAKT;FTDNAKTII;IIVQLNTSV;IVQLNTSVE;VQLNTSVEI; |
| Env | HSFNCGGEFFYCNSTQLFNSTW | 374-395  (380-389) | 0.857 | HSFNCGGEF;SFNCGGEFF;FYCNSTQLF;STQLFNSTW; | - |
| Env | IKQIINMWQKVGKAMYAPPISGQIRCSS | 420-447  (420-441) | 0.670 | - | IKQIINMWQ;INMWQKVGK;MWQKVGKAM;WQKVGKAMY;YAPPISGQI;ISGQIRCSS; |
| Env | MTLTVQARQLLSGIVQQQNNLLR | 535-557  (542-550) | 0.379 | - | MTLTVQARQ;LTVQARQLL;IVQQQNNLL;VQQQNNLLR; |
| **Env** | **LEQIWNHTTWMEWDREINNYTSLIHSLI** | **619-646**  **(624-643)** | **0.999** | **EQIWNHTTW;TWMEWDREI;EWDREINNY;REINNYTSL;NYTSLIHSL;LEQIWNHTT;WMEWDREIN;MEWDREINN;WDREINNYT;YTSLIHSLI;** | |
| Env | LEQIWNHTTWMEWDREINNYTSLIHSLI | 619-646  (624-643) | 0.933 | - | LEQIWNHTT;WMEWDREIN;MEWDREINN;WDREINNYT;YTSLIHSLI; |
|  | EQIWNHTTWMEWDREINNYT  SLIHS | 620-644  (626-642) | 0.994 | EQIWNHTTW;TWMEWDREI;EWDREINNY;REINNYTSL;NYTSLIHSL; | - |
| Env | GLRIVFAVLSIVNRVRQGYSP  LSFQTHL | 694-721  (700-719) | 0.971 | GLRIVFAVL;RIVFAVLSI;IVNRVRQGY;RVRQGYSPL;RQGYSPLSF;SPLSFQTHL; | - |
| Env | GTDRVIEVVQGACRAIRHIPRRI | 825-847  (832-840) | 0.371 | GTDRVIEVV;RVIEVVQGA;IEVVQGACR;AIRHIPRRI; | - |
| Gag | IVWASRELERFAVNPGLLE | 34-52  (37-49) | 0.741 | - | IVWASRELE;VWASRELER;ERFAVNPGL;FAVNPGLLE; |
| Gag | HSNQVSQNYPIVQNIQGQMVHQAISPRTLNAWVKVVEEKAFSPEV | 124-168  (128-162) | 0.920 | HSNQVSQNY;NQVSQNYPI;IVQNIQGQM;VQNIQGQMV;MVHQAISPR;HQAISPRTL;ISPRTLNAW;SPRTLNAWV;RTLNAWVKV;EEKAFSPEV; | - |
| **Gag** | MLKETINEEAAEWDRVHPVHAGPIA | 200-224  (205-217) | 0.990 | KETINEEAA;TINEEAAEW;AEWDRVHPV;HPVHAGPIA;MLKETINEE;LKETINEEA;INEEAAEWD;WDRVHPVHA;VHPVHAGPI; | |
| Gag | MLKETINEEAAEWDRVHPVHAGPI | 200-223  (205-216) | 0.619 | - | MLKETINEE;LKETINEEA;INEEAAEWD;WDRVHPVHA;VHPVHAGPI; |
| Gag | KETINEEAAEWDRVHPIA | 202-219  (209-217) | 0.974 | KETINEEAA;TINEEAAEW;AEWDRVHPV;HPVHAGPIA; | - |
| **Gag** | **ILGLNKIVRMYSPTSILDIRQGPKEPFRDYVDRFY** | **267-301**  **(270-297)** | **0.984** | **ILGLNKIVR;LNKIVRMYS;IVRMYSPTS;YSPTSILDI;RMYSPTSIL;ILDIRQGPK;RQGPKEPFR;GPKEPFRDY;FRDYVDRFY** | |
|  |  |  |  |  |  |
| Gag | ILGLNKIVRMYSPTSILDI | 267-285  (270-282) | 0.727 | - | ILGLNKIVR;LNKIVRMYS;IVRMYSPTS;YSPTSILDI; |
| Gag | RMYSPTSILDIRQGPKEPFRDYVDRFY | 275-301  (281-297) | 0.940 | RMYSPTSIL;ILDIRQGPK;RQGPKEPFR;GPKEPFRDY;FRDYVDRFY; | - |
| Gag | LLVQNANPDCKTILKALGPAATLEEMMTACQGVGG | 321-355  (327-349) | 0.724 | - | LLVQNANPD;LVQNANPDC;ILKALGPAA;LKALGPAAT;LGPAATLEE;LEEMMTACQ;MTACQGVGG; |
| **Gag** | **GPGHKARVLAEAMSQVTNSATIMMQRGNFR** | **355-384**  **(361-378)** | **0.736** | **GPGHKARVL;KARVLAEAM;VLAEAMSQV;SQVTNSATI;TIMMQRGNF;IMMQRGNFR;LAEAMSQVT;MSQVTNSAT;VTNSATIMM;IMMQRGNFR;** | |
| Gag | GPGHKARVLAEAMSQVTNSATIMQRGNFR | 355-383  (361-377) | 0.612 | GPGHKARVL;KARVLAEAM;VLAEAMSQV;SQVTNSATI;TIMMQRGNF;IMMQRGNFR; | - |
| Gag | LAEAMSQVTNSATIMMQRGNFR | 363-384  (369-378) | 0.320 | - | LAEAMSQVT;MSQVTNSAT;VTNSATIMM;IMMQRGNFR; |
| Gag | TIMMQRGNFRNQRKIVKCFNCGK | 375-397  (382-390) | 0.478 | TIMMQRGNF;IMMQRGNFR;NQRKIVKCF;IVKCFNCGK; | - |
| **Nef** | **FPVTPQVPLRPMTYKAAVDLSHFLKEKGGLEGLIHSQRRQDI** | **68-109**  **(75-102)** | **0.999** | **FPVTPQVPL;TPQVPLRPM;RPMTYKAAV;KAAVDLSHF;AVDLSHFLK;MTYKAAVDL;YKAAVDLSH;LSHFLKEKG;FLKEKGGLE;LIHSQRRQD;IHSQRRQDI;** | |
| Nef | FPVTPQVPLRPMTYKAAVDLSHFLKPLRPMTYKAAVD | 68-92  (75-86) | 0.999 | FPVTPQVPL;TPQVPLRPM;RPMTYKAAV;KAAVDLSHF;AVDLSHFLK; | - |
| Nef | MTYKAAVDLSHFLKEKGGLEGLIHSQRRQDI | 79-109  (83-102) | 0.732 | - | MTYKAAVDL;YKAAVDLSH;LSHFLKEKG;FLKEKGGLE;LIHSQRRQD;IHSQRRQDI; |
| Nef | RQDILDLWIYHTQGYFPDWQNY | 106-127  (112-121) | 0.814 | RQDILDLWI;WIYHTQGYF;HTQGYFPDW;GYFPDWQNY; | - |
| Nef | YPLTFGWCYKLVPVEPDKIEEANKG | 135-159  (138-158) | 0.567 | - | YPLTFGWCY;FGWCYKLVP;YKLVPVEPD;LVPVEPDKI;VEPDKIEEA;DKIEEANKG; |
| Nef | GMDDPEREVLEWRFDSRLAFHHV | 172-194  (179-187) | 0.632 | GMDDPEREV;DPEREVLEW;LEWRFDSRL;DSRLAFHHV; | - |
| Nef | EWRFDSRLAFHHVARELHPEY | 182-202  (187-197) | 0.805 | - | EWRFDSRLA;WRFDSRLAF;FHHVARELH;VARELHPEY; |
| Pol | FREDLAFLQGKAREFS | 2-17  (9-17) | 0.657 | - | FREDLAFLQ;LAFLQGKAR;LQGKAREFS |
| Pol | RQGTVSFNFPQVTLWQRPLVTIK | 48-70  (55-63) | 0.585 | RQGTVSFNF;TVSFNFPQV;FPQVTLWQR;WQRPLVTIK; | - |
| Pol | KVRQYDQILIEICGHKAI | 111-128  (113-126) | 0.759 | KVRQYDQIL;QYDQILIEI;ILIEICGHK;IEICGHKAI; | - |
| Pol | LIEICGHKAIGTVLVGPTPVNII | 119-141  (126-134) | 0.592 | - | LIEICGHKA;IEICGHKAI;VLVGPTPVN;VGPTPVNII; |
| Pol | LLTQIGCTLNFPISPIETVPVK | 145-166  (151-160) | 0.704 | LLTQIGCTL;TLNFPISPI;FPISPIETV;SPIETVPVK; | - |
| Pol | LVEICTEMEKEGKISK | 189-204 | 0.350 | - | LVEICTEME;VEICTEMEK;ICTEMEKEG;MEKEGKISK; |
| Pol | YTAFTIPSINNETPGIRYQYNVLPQGW | 282-308  (289-304) | 0.956 | YTAFTIPSI;SINNETPGI;ETPGIRYQY;GIRYQYNVL;QYNVLPQGW; | - |
| Pol | KQNPDIVIYQYMDDLYV | 328-344  (329-343) | 0.691 | KQNPDIVIY;NPDIVIYQY;IYQYMDDLY;YQYMDDLYV; | - |
| Pol | IEELRQHLLRWGLTTPD  KKHQKEPPFLWMGY | 357-387  (359-381) | 0.961 | IEELRQHLL;EELRQHLLR;RQHLLRWGL;RWGLTTPDK;TTPDKKHQK;HQKEPPFLW;EPPFLWMGY; | - |
| Pol | IQKQGQGQWTYQIYQEPFK | 484-502  (487-499) | 0.931 | IQKQGQGQW;KQGQGQWTY;TYQIYQEPF;YQIYQEPFK; | - |
| Pol | VQKITTESIVIWGKTPKFKLPIQKETWETW | 527-556  (533-550) | 0.881 | VQKITTESI;TESIVIWGK;IVIWGKTPK;KTPKFKLPI;KLPIQKETW;IQKETWETW | - |
| Pol | WTEYWQATWIPEWEFVNTPPLVK | 557-579  (562-576) | 0.971 | WTEYWQATW;WQATWIPEW;ATWIPEWEF;WEFVNTPPL;FVNTPPLVK; | - |
| Pol | VVESMNKELKKIIGQVRDQA | 865-884  (866-881) | 0.526 | - | VVESMNKEL;VESMNKELK;MNKELKKII;LKKIIGQVR;IIGQVRDQA; |
| Pol | QVRDQAEHLKTAVQMAVFIHNFKRKGGIGGY | 879-909  (886-903) | 0.942 | QVRDQAEHL;KTAVQMAVF;QMAVFIHNF;MAVFIHNFK;KRKGGIGGY; | - |
| Pol | YSAGERIVDIIATDIQTKE | 909-927  (912-924) | 0.564 | - | YSAGERIVD;IVDIIATDI;VDIIATDIQ;IATDIQTKE; |
| Pol | ATDIQTKELQKQITKIQNFRV | 920-940  (925-935) | 0.624 | ATDIQTKEL;KELQKQITK;KQITKIQNF;ITKIQNFRV; | - |
| Pol | ITKIQNFRVYYRDSRNPLW | 932-950  (935-947) | 0.857 | - | ITKIQNFRV;FRVYYRDSR;YYRDSRNPL;YRDSRNPLW; |
| Rev | RSGDSDEELIRTVRLIKLLY | 4-23  (8-19) | 0.940 | RSGDSDEEL;EELIRTVRL;RTVRLIKLL;TVRLIKLLY; | - |
| Rev | RWRERQRQIHSISERILGTYLGRSAEPVPLQLPP | 44-77  (48-70) | 0.952 | RWRERQRQI;ERQRQIHSI;RQIHSISER;ISERILGTY;SERILGTYL;YLGRSAEPV;EPVPLQLPP; | - |
| Rev | VPLQLPPLERLTLDCNEDCGTSGTQGV | 71-97  (74-95) | 0.846 | - | VPLQLPPLE;LPPLERLTL;RLTLDCNED;LTLDCNEDC;CNEDCGTSG;CGTSGTQGV; |
| Rev | TQGVGSPQILVESPTVL | 94-110  (95-109) | 0.939 | TQGVGSPQI;GVGSPQILV;QILVESPTV;ILVESPTVL; | - |
| Tat | MEPVDPRLEPWKHPGSQPK | 1-14  (4-16) | 0.565 | - | MEPVDPRLE;EPVDPRLEP;LEPWKHPGS;WKHPGSQPK; |
| Tat | FHCQVCFITKALGISYGRKKRRQR | 32-55  (32-51) | 0.629 | - | FHCQVCFIT;VCFITKALG;CFITKALGI;ITKALGISY;YGRKKRRQR; |
| **Vif** | **VWQVDRMRIRTWKSLVKHHMYVSGKARGWFYRHHY** | **11-44**  **(14-37)** | **0.885** | **RIRTWKSLV;KSLVKHHMY;SLVKHHMYV;YVSGKARGW;RGWFYRHHY;VWQVDRMRI;WQVDRMRIR;IRTWKSLVK;WKSLVKHHM** | |
| Vif | VWQVDRMRIRTWKSLVKHHM | 10-29  (14-25) | 0.661 | - | VWQVDRMRI;WQVDRMRIR;IRTWKSLVK;WKSLVKHHM; |
| Vif | RIRTWKSLVKHHMYVSGKARGWFYRHHY | 17-44  (23-37) | 0.661 | RIRTWKSLV;KSLVKHHMY;SLVKHHMYV;YVSGKARGW;RGWFYRHHY; | - |
| Vif | PLGDARLVITTYWGLHTGERDWHLGQGVSI | 58-87  (65-84) | 0.447 | - | PLGDARLVI;YWGLHTGER;WGLHTGERD;LHTGERDWH;WHLGQGVSI; |
| Vif | SLQYLALAALITPKKIKPPLPSVTKLTEDRWNKPQKTK | 144-181  (148-175) | 0.995 | SLQYLALAA;LQYLALAAL;ALAALITPK;TPKKIKPPL;KIKPPLPSV;KPPLPSVTK;VTKLTEDRW;KLTEDRWNK;RWNKPQKTK; | - |
| Vpr | YETYGDTWAGVEAIIRILQ | 47-65  (50-62) | 0.464 | - | YETYGDTWA;YGDTWAGVE;WAGVEAIIR;VEAIIRILQ; |
| **Vpu** | **VIIEYRKILRQRKIDRLIDRLIER** | **26-49**  **(32-44)** | **0.907** | **EYRKILRQR;RQRKIDRLI;KIDRLIDRL;RLIDRLIER;VIIEYRKIL;YRKILRQRK;ILRQRKIDR;IDRLIDRLI;** | |
| Vpu | VIIEYRKILRQRKIDRLI | 26-43  (32-41) | 0.502 | - | VIIEYRKIL;YRKILRQRK;ILRQRKIDR;IDRLIDRLI; |
| Vpu | EYRKILRQRKIDRLIDRLIER | 29-49  (34-44) | 0.813 | EYRKILRQR;RQRKIDRLI;KIDRLIDRL;RLIDRLIER; | - |

**Table S10.** Japanese-specific CD8+ T-cell epitopes derived from the HIV-dataset (Los Alamos HIV Molecular Immunology Database) that were colocalized with HIV-1 specific CD8+ T-cell epitope clusters predicted by Predivac-3.0.

| **No.** | **Published**  **epitopes** | **Protein** | **Amino acid position (HXB2)** | **Predicted cluster** |
| --- | --- | --- | --- | --- |
| 1 | TVYYGVPVW | Env | 37-45 |  |
| 2 | VPVWKEATTTL | Env | 42-52 |  |
| 3 | EVHNVWATHA | Env | 63-72 |  |
| 4 | DPNPQEVVL | Env | 78-86 |  |
| 5 | TAVPWNASW | Env | 606-614 |  |
| 6 | DLRSLCLFSY | Env | 758-767 |  |
| 7 | IPRRIRQGL | Env | 843-851 |  |
| 8 | RPGGKKKYK | Gag | 22-30 |  |
| 9 | KYKLKHIVW | Gag | 28-36 |  |
| 10 | LKHIVWASREL | Gag | 31-41 | **IVWASREL**ERFAVNPGLLE |
| 11 | RELERFAV | Gag | 39-46 | IVWAS**RELERFAV**NPGLLE |
| 12 | SLYNTVATL | Gag | 77-85 |  |
| 13 | TVATLYCVH | Gag | 81-89 |  |
| 14 | SPRTLNAWV | Gag | 148-156 | HSNQVSQNYPIVQNIQGQMVHQAI**SPRTLNAWV**KVVEEKAFSPEV |
| 15 | KAFSPEVIPMF | Gag | 162-172 |  |
| 16 | EVIPMFSAL | Gag | 167-175 |  |
| 17 | EGATPQDLNTM | Gag | 177-187 |  |
| 18 | TPQDLNTML | Gag | 180-188 |  |
| 19 | DLNTMLNTV | Gag | 183-191 |  |
| 20 | AMQMLKETI | Gag | 197-205 |  |
| 21 | KETINEEAA | Gag | 202-210 | ML**KETINEEAA**EWDRVHPVHAGPIA |
| 22 | AEWDRVHPV | Gag | 210-218 | MLKETINEEA**AEWDRVHPV**HAGPIA |
| 23 | HPVHAGPIA | Gag | 216-224 | MLKETINEEAAEWDRV**HPVHAGPIA** |
| 24 | DIAGTTSTL | Gag | 235-243 |  |
| 25 | TSTLQEQIGW | Gag | 240-249 |  |
| 26 | NPPIPVGEIY | Gag | 253-262 |  |
| 27 | IYKRWIILG | Gag | 261-269 |  |
| 28 | IILGLNKIV | Gag | 266-274 | **ILGLNKIV**RMYSPTSILDIRQGPKEPFRDYVDRFY |
| 29 | GLNKIVRMY | Gag | 269-277 | IL**GLNKIVRMY**SPTSILDIRQGPKEPFRDYVDRFY |
| 30 | RMYSPTSI | Gag | 275-282 | ILGLNKIV**RMYSPTSI**LDIRQGPKEPFRDYVDRFY |
| 31 | YSPTSILDI | Gag | 277-285 | ILGLNKIVRM**YSPTSILDI**RQGPKEPFRDYVDRFY |
| 32 | DYVDRFYKT | Gag | 295-303 | ILGLNKIVRMYSPTSILDIRQGPKEPFR**DYVDRFY** |
| 33 | DYVDRFYKTLR | Gag | 295-305 |  |
| 34 | VKNWMTETL | Gag | 313-321 |  |
| 35 | WMTETLLV | Gag | 316-323 |  |
| 36 | NANPDCKTI | Gag | 325-333 | LLVQ**NANPDCKTI**LKALGPAATLEEMMTACQGVGG |
| 37 | NPDCKTILKAL | Gag | 327-337 |  |
| 38 | KALGPAATL | Gag | 335-343 | LLVQNANPDCKTIL**KALGPAATL**EEMMTACQGVGG |
| 39 | GPAATLEEM | Gag | 338-346 |  |
| 40 | ATLEEMMTA | Gag | 341-349 | LLVQNANPDCKTILKALGPA**ATLEEMMTA**CQGVGG |
| 41 | ACQGVGGPGHK | Gag | 349-359 | LLVQNANPDCKTILKALGPAATLEEMMT**ACQGVGG** |
| 42 | GPGHKARVL | Gag | 355-363 | **GPGHKARVL**AEAMSQVTNSATIMMQRGNFR |
| 43 | TERQANFL | Gag | 427-434 |  |
| 44 | RQANFLGKI | Gag | 429-437 |  |
| 45 | FLGKIWPS | Gag | 433-440 |  |
| 46 | ELYPLTSLRSL | Gag | 482-492 |  |
| 47 | TPQVPLRPMTY | Nef | 71-81 | FPV**TPQVPLRPMTY**KAAVDLSHFLKPLRPMTYKAAVD |
| 48 | VPLRPMTY | Nef | 74-81 | FPVTPQ**VPLRPMTY**KAAVDLSHFLKEKGGLEGLIHSQRRQDI |
| 49 | RPMTYKAAV | Nef | 77-85 |  |
| 50 | AVDLSHFLK | Nef | 84-92 | FPVTPQVPLRPMTYKA**AVDLSHFLK**PLRPMTYKAAVD |
| 51 | DLWIYHTQGYF | Nef | 111-121 | RQDIL**DLWIYHTQGYF**PDWQNY |
| 52 | FPDWQNYTP | Nef | 121-129 | RQDILDLWIYHTQGY**FPDWQNY** |
| 53 | RYPLTFGW | Nef | 134-141 | **YPLTFGW**CYKLVPVEPDKIEEANKG |
| 54 | YPLTFGWCY | Nef | 135-143 | **YPLTFGWCY**KLVPVEPDKIEEANKG |
| 55 | WQRPLVTI | Pol | 62-69 | RQGTVSFNFPQVTL**WQRPLVTI**K |
| 56 | EICGHKAIGTV | Pol | 121-131 | LI**EICGHKAIGTV**LVGPTPVNII |
| 57 | FPISPIETV | Pol | 155-163 | LLTQIGCTLNF**PISPIETV**PVK |
| 58 | ETVPVKLKPGM | Pol | 161-171 |  |
| 59 | ELNKRTQDF | Pol | 234-242 |  |
| 60 | EVQLGIPHPA | Pol | 244-253 |  |
| 61 | TVLDVGDAY | Pol | 262-270 |  |
| 62 | VPLDEDFRKY | Pol | 273-282 |  |
| 63 | YTAFTIPSI | Pol | 282-290 | **YTAFTIPSI**NNETPGIRYQYNVLPQGW |
| 64 | ETPGIRYQY | Pol | 293-301 | YTAFTIPSINN**ETPGIRYQY**NVLPQGW |
| 65 | LPQGWKGSPA | Pol | 304-313 |  |
| 66 | SPAIFQSSM | Pol | 311-319 |  |
| 67 | AIFQSSMTK | Pol | 313-321 |  |
| 68 | KQNPDIVIY | Pol | 328-336 | **KQNPDIVIY**QYMDDLYV |
| 69 | NPDIVIYQY | Pol | 330-338 | KQ**NPDIVIYQY**MDDLYV |
| 70 | VIYQYMDDL | Pol | 334-342 | KQNPDI**VIYQYMDDL**YV |
| 71 | YQYMDDLYV | Pol | 336-344 | KQNPDIVI**YQYMDDLYV** |
| 72 | ELRQHLLRW | Pol | 359-367 | IE**ELRQHLLRW**GLTTPDKKHQKEPPFLWMGY |
| 73 | DIQKLVGKL | Pol | 411-419 |  |
| 74 | EVIPLTEEA | Pol | 446-454 |  |
| 75 | IPLTEEAEL | Pol | 448-456 |  |
| 76 | ILKEPVHGVYY | Pol | 464-474 |  |
| 77 | IQKQGQGQWTY | Pol | 484-494 | IQ**KQGQGQWTY**QIYQEPFK |
| 78 | IYQEPFKNL | Pol | 496-504 | IQKQGQGQWTYQ**IYQEPFK** |
| 79 | DVKQLTEAV | Pol | 519-527 |  |
| 80 | EPIVGAETFY | Pol | 587-596 |  |
| 81 | FYVDGAANR | Pol | 595-603 |  |
| 82 | ETKLGKAGY | Pol | 604-612 |  |
| 83 | DTTNQKTEL | Pol | 626-634 |  |
| 84 | EVNIVTDSQY | Pol | 647-656 |  |
| 85 | SQYALGII | Pol | 654-661 |  |
| 86 | QIIEQLIKK | Pol | 675-683 |  |
| 87 | LPPVVAKEI | Pol | 743-751 |  |
| 88 | EIVASCDKCQL | Pol | 750-760 |  |
| 89 | GQVDCSPGI | Pol | 767-775 |  |
| 90 | LEGKVILVA | Pol | 783-791 |  |
| 91 | HVASGYIEA | Pol | 793-801 |  |
| 92 | GYIEAEVI | Pol | 797-804 |  |
| 93 | IEAEVIPAET | Pol | 799-808 |  |
| 94 | ETGQETAYFLL | Pol | 807-817 |  |
| 95 | GQETAYFLL | Pol | 809-817 |  |
| 96 | ELKKIIGQV | Pol | 872-880 | VVESMNK**ELKKIIGQV**RDQA |
| 97 | EHLKTAVQMA | Pol | 885-894 | QVRDQA**EHLKTAVQMA**VFIHNFKRKGGIGGY |
| 98 | GERIVDIIA | Pol | 912-920 | YSA**GERIVDIIA**TDIQTKE |
| 99 | IIATDIQTK | Pol | 918-926 | YSAGERIVD**IIATDIQTK**E |
| 100 | TDIQTKEL | Pol | 921-928 | A**TDIQTKEL**QKQITKIQNFRV |
| 101 | LQKQITKI | Pol | 928-935 | ATDIQTKE**LQKQITKI**QNFRV |
| 102 | HPRISSEVHI | Vif | 48-57 |  |
| 103 | AIIRILQQL | Vpr | 59-67 | YETYGDTWAGVE**AIIRILQ** |

**Table S11**. Full list of 9 residues long HIV-1 specific T-cell epitopes associated to the clusters predicted by Predivac-3.0 for the Japanese population, including the corresponding protein, sequence, position (start/end), allele restriction (HLA class I or class II), number of epitopes associated with this region, predicted population coverage and statistical significance of each sequence (p-values).

| **Protein** | **Sequence** | **Start** | **End** | **HLA**  **class I** | **HLA class II** | **Number of**  **epitopes** | **Coverage**  **(%)** | **p-value** |
| --- | --- | --- | --- | --- | --- | --- | --- | --- |
| Env | NMWKNDMVE | 94 | 102 | - | X | 4 | 0.639637 | 4.60E-20 |
| Env | MWKNDMVEQ | 95 | 103 | - | X | 4 | 0.639637 | 4.60E-20 |
| Env | WKNDMVEQM | 96 | 104 | - | X | 4 | 0.639637 | 4.60E-20 |
| Env | QSLKPCVKL | 114 | 122 | X | - | 4 | 0.925968 | 5.41E-43 |
| Env | SLKPCVKLT | 115 | 123 | X | - | 4 | 0.925968 | 5.41E-43 |
| Env | SGRMIMEKG | 144 | 152 | X | - | 4 | 0.700105 | 5.41E-43 |
| Env | GRMIMEKGE | 145 | 153 | X | - | 4 | 0.700105 | 5.41E-43 |
| Env | RMIMEKGEI | 146 | 154 | X | - | 4 | 0.700105 | 5.41E-43 |
| Env | EKGEIKNCS | 150 | 158 | X | - | 4 | 0.677306 | 5.41E-43 |
| Env | FFYKLDIIP | 175 | 183 | - | X | 4 | 0.795078 | 4.60E-20 |
| Env | FYKLDIIPI | 176 | 184 | - | X | 4 | 0.795078 | 4.60E-20 |
| Env | YKLDIIPID | 177 | 185 | - | X | 5 | 0.810878 | 1.49E-118 |
| Env | KLDIIPIDN | 178 | 186 | - | X | 5 | 0.810878 | 1.49E-118 |
| Env | LDIIPIDND | 179 | 187 | - | X | 5 | 0.810878 | 1.49E-118 |
| Env | DIIPIDNDT | 180 | 188 | - | X | 5 | 0.810878 | 1.49E-118 |
| Env | IIPIDNDTT | 181 | 189 | - | X | 4 | 0.748276 | 4.60E-20 |
| Env | IPIDNDTTS | 182 | 190 | - | X | 4 | 0.748276 | 4.60E-20 |
| Env | PIDNDTTSY | 183 | 191 | - | X | 4 | 0.748276 | 4.60E-20 |
| Env | IDNDTTSYK | 184 | 192 | - | X | 5 | 0.757376 | 1.49E-118 |
| Env | DNDTTSYKL | 185 | 193 | - | X | 4 | 0.592521 | 4.60E-20 |
| Env | NDTTSYKLT | 186 | 194 | - | X | 5 | 0.592521 | 1.49E-118 |
| Env | DTTSYKLTS | 187 | 195 | - | X | 5 | 0.592521 | 1.49E-118 |
| Env | TTSYKLTSC | 188 | 196 | - | X | 5 | 0.592521 | 1.49E-118 |
| Env | TSYKLTSCN | 189 | 197 | - | X | 4 | 0.349776 | 4.60E-20 |
| Env | ACPKVSFEP | 204 | 212 | X | - | 4 | 0.841077 | 5.41E-43 |
| Env | CPKVSFEPI | 205 | 213 | X | - | 4 | 0.841077 | 5.41E-43 |
| Env | PKVSFEPIP | 206 | 214 | X | - | 5 | 0.885196 | 4.48E-226 |
| Env | KVSFEPIPI | 207 | 215 | X | - | 4 | 0.820262 | 5.41E-43 |
| Env | VSFEPIPIH | 208 | 216 | X | - | 4 | 0.820262 | 5.41E-43 |
| Env | SFEPIPIHY | 209 | 217 | X | - | 4 | 0.820262 | 5.41E-43 |
| Env | FEPIPIHYC | 210 | 218 | X | - | 4 | 0.820262 | 5.41E-43 |
| Env | EPIPIHYCA | 211 | 219 | X | - | 4 | 0.820262 | 5.41E-43 |
| Env | PIPIHYCAP | 212 | 220 | X | - | 5 | 0.882294 | 4.48E-226 |
| Env | IPIHYCAPA | 213 | 221 | X | - | 4 | 0.844119 | 5.41E-43 |
| Env | PIHYCAPAG | 214 | 222 | X | - | 4 | 0.844119 | 5.41E-43 |
| Env | HYCAPAGFA | 216 | 224 | X | - | 4 | 0.580909 | 5.41E-43 |
| Env | YCAPAGFAI | 217 | 225 | X | - | 4 | 0.580909 | 5.41E-43 |
| Env | CAPAGFAIL | 218 | 226 | X | - | 5 | 0.688096 | 4.48E-226 |
| Env | APAGFAILK | 219 | 227 | X | - | 4 | 0.688096 | 5.41E-43 |
| Env | PAGFAILKC | 220 | 228 | X | - | 4 | 0.688096 | 5.41E-43 |
| Env | GIRPVVSTQ | 250 | 258 | X | - | 4 | 0.919887 | 5.41E-43 |
| Env | TDNAKTIIV | 278 | 286 | - | X | 4 | 0.405538 | 4.60E-20 |
| Env | DNAKTIIVQ | 279 | 287 | - | X | 5 | 0.405538 | 1.49E-118 |
| Env | NAKTIIVQL | 280 | 288 | - | X | 5 | 0.405538 | 1.49E-118 |
| Env | AKTIIVQLN | 281 | 289 | - | X | 5 | 0.405538 | 1.49E-118 |
| Env | KTIIVQLNT | 282 | 290 | - | X | 5 | 0.405538 | 1.49E-118 |
| Env | TIIVQLNTS | 283 | 291 | - | X | 4 | 0.321441 | 4.60E-20 |
| Env | IIVQLNTSV | 284 | 292 | - | X | 4 | 0.321441 | 4.60E-20 |
| Env | GEFFYCNST | 380 | 388 | X | - | 4 | 0.857048 | 5.41E-43 |
| Env | EFFYCNSTQ | 381 | 389 | X | - | 4 | 0.857048 | 5.41E-43 |
| Env | IKQIINMWQ | 420 | 428 | - | X | 4 | 0.667506 | 4.60E-20 |
| Env | KQIINMWQK | 421 | 429 | - | X | 4 | 0.667506 | 4.60E-20 |
| Env | QIINMWQKV | 422 | 430 | - | X | 4 | 0.667506 | 4.60E-20 |
| Env | IINMWQKVG | 423 | 431 | - | X | 4 | 0.667506 | 4.60E-20 |
| Env | INMWQKVGK | 424 | 432 | - | X | 4 | 0.667506 | 4.60E-20 |
| Env | NMWQKVGKA | 425 | 433 | - | X | 4 | 0.667506 | 4.60E-20 |
| Env | MWQKVGKAM | 426 | 434 | - | X | 4 | 0.667506 | 4.60E-20 |
| Env | WQKVGKAMY | 427 | 435 | - | X | 4 | 0.667506 | 4.60E-20 |
| Env | QKVGKAMYA | 428 | 436 | - | X | 4 | 0.348963 | 4.60E-20 |
| Env | KVGKAMYAP | 429 | 437 | - | X | 4 | 0.348963 | 4.60E-20 |
| Env | VGKAMYAPP | 430 | 438 | - | X | 4 | 0.348963 | 4.60E-20 |
| Env | GKAMYAPPI | 431 | 439 | - | X | 4 | 0.348963 | 4.60E-20 |
| Env | KAMYAPPIS | 432 | 440 | - | X | 4 | 0.352887 | 4.60E-20 |
| Env | AMYAPPISG | 433 | 441 | - | X | 4 | 0.352887 | 4.60E-20 |
| Env | RQLLSGIVQ | 542 | 550 | - | X | 4 | 0.379095 | 4.60E-20 |
| Env | NHTTWMEWD | 624 | 632 | - | X | 4 | 0.932153 | 4.60E-20 |
| Env | HTTWMEWDR | 625 | 633 | - | X | 4 | 0.932153 | 4.60E-20 |
| Env | TTWMEWDRE | 626 | 634 | - | X | 4 | 0.932153 | 4.60E-20 |
| Env | TTWMEWDRE | 626 | 634 | X | - | 4 | 0.9939 | 5.41E-43 |
| Env | TWMEWDREI | 627 | 635 | X | - | 4 | 0.9939 | 5.41E-43 |
| Env | EWDREINNY | 630 | 638 | X | - | 4 | 0.960732 | 5.41E-43 |
| Env | WDREINNYT | 631 | 639 | - | X | 4 | 0.933155 | 4.60E-20 |
| Env | WDREINNYT | 631 | 639 | X | - | 4 | 0.960732 | 5.41E-43 |
| Env | DREINNYTS | 632 | 640 | - | X | 4 | 0.933155 | 4.60E-20 |
| Env | DREINNYTS | 632 | 640 | X | - | 4 | 0.960732 | 5.41E-43 |
| Env | REINNYTSL | 633 | 641 | - | X | 4 | 0.933155 | 4.60E-20 |
| Env | REINNYTSL | 633 | 641 | X | - | 4 | 0.960732 | 5.41E-43 |
| Env | EINNYTSLI | 634 | 642 | - | X | 4 | 0.933155 | 4.60E-20 |
| Env | EINNYTSLI | 634 | 642 | X | - | 4 | 0.960732 | 5.41E-43 |
| Env | INNYTSLIH | 635 | 643 | - | X | 4 | 0.933155 | 4.60E-20 |
| Env | AVLSIVNRV | 700 | 708 | X | - | 4 | 0.882917 | 5.41E-43 |
| Env | VLSIVNRVR | 701 | 709 | X | - | 4 | 0.882917 | 5.41E-43 |
| Env | LSIVNRVRQ | 702 | 710 | X | - | 4 | 0.886675 | 5.41E-43 |
| Env | SIVNRVRQG | 703 | 711 | X | - | 4 | 0.886675 | 5.41E-43 |
| Env | NRVRQGYSP | 706 | 714 | X | - | 4 | 0.97066 | 5.41E-43 |
| Env | RVRQGYSPL | 707 | 715 | X | - | 4 | 0.97066 | 5.41E-43 |
| Env | VRQGYSPLS | 708 | 716 | X | - | 4 | 0.97066 | 5.41E-43 |
| Env | RQGYSPLSF | 709 | 717 | X | - | 4 | 0.97066 | 5.41E-43 |
| Env | QGYSPLSFQ | 710 | 718 | X | - | 4 | 0.97066 | 5.41E-43 |
| Env | GYSPLSFQT | 711 | 719 | X | - | 4 | 0.97066 | 5.41E-43 |
| Env | VVQGACRAI | 832 | 840 | X | - | 4 | 0.371197 | 5.41E-43 |
| Gag | ASRELERFA | 37 | 45 | - | X | 4 | 0.741234 | 4.60E-20 |
| Gag | SRELERFAV | 38 | 46 | - | X | 4 | 0.741234 | 4.60E-20 |
| Gag | RELERFAVN | 39 | 47 | - | X | 4 | 0.741234 | 4.60E-20 |
| Gag | ELERFAVNP | 40 | 48 | - | X | 4 | 0.741234 | 4.60E-20 |
| Gag | LERFAVNPG | 41 | 49 | - | X | 4 | 0.741234 | 4.60E-20 |
| Gag | VSQNYPIVQ | 128 | 136 | X | - | 4 | 0.797404 | 5.41E-43 |
| Gag | SQNYPIVQN | 129 | 137 | X | - | 4 | 0.797404 | 5.41E-43 |
| Gag | QNYPIVQNI | 130 | 138 | X | - | 4 | 0.797404 | 5.41E-43 |
| Gag | NYPIVQNIQ | 131 | 139 | X | - | 4 | 0.797404 | 5.41E-43 |
| Gag | NIQGQMVHQ | 137 | 145 | X | - | 4 | 0.3379 | 5.41E-43 |
| Gag | IQGQMVHQA | 138 | 146 | X | - | 4 | 0.3379 | 5.41E-43 |
| Gag | QGQMVHQAI | 139 | 147 | X | - | 4 | 0.3379 | 5.41E-43 |
| Gag | GQMVHQAIS | 140 | 148 | X | - | 5 | 0.434779 | 4.48E-226 |
| Gag | QMVHQAISP | 141 | 149 | X | - | 6 | 0.5966 | 0 |
| Gag | MVHQAISPR | 142 | 150 | X | - | 5 | 0.472494 | 4.48E-226 |
| Gag | VHQAISPRT | 143 | 151 | X | - | 5 | 0.472494 | 4.48E-226 |
| Gag | HQAISPRTL | 144 | 152 | X | - | 5 | 0.472494 | 4.48E-226 |
| Gag | QAISPRTLN | 145 | 153 | X | - | 5 | 0.472494 | 4.48E-226 |
| Gag | AISPRTLNA | 146 | 154 | X | - | 5 | 0.472494 | 4.48E-226 |
| Gag | ISPRTLNAW | 147 | 155 | X | - | 5 | 0.472494 | 4.48E-226 |
| Gag | SPRTLNAWV | 148 | 156 | X | - | 5 | 0.472494 | 4.48E-226 |
| Gag | PRTLNAWVK | 149 | 157 | X | - | 5 | 0.472494 | 4.48E-226 |
| Gag | RTLNAWVKV | 150 | 158 | X | - | 4 | 0.434107 | 5.41E-43 |
| Gag | TLNAWVKVV | 151 | 159 | X | - | 4 | 0.434107 | 5.41E-43 |
| Gag | NAWVKVVEE | 153 | 161 | X | - | 4 | 0.438983 | 5.41E-43 |
| Gag | AWVKVVEEK | 154 | 162 | X | - | 4 | 0.438983 | 5.41E-43 |
| Gag | INEEAAEWD | 205 | 213 | - | X | 4 | 0.618985 | 4.60E-20 |
| Gag | NEEAAEWDR | 206 | 214 | - | X | 4 | 0.618985 | 4.60E-20 |
| Gag | EEAAEWDRV | 207 | 215 | - | X | 4 | 0.618985 | 4.60E-20 |
| Gag | EAAEWDRVH | 208 | 216 | - | X | 4 | 0.618985 | 4.60E-20 |
| Gag | AAEWDRVHP | 209 | 217 | X | - | 4 | 0.974384 | 5.41E-43 |
| Gag | LNKIVRMYS | 270 | 278 | - | X | 4 | 0.72677 | 4.60E-20 |
| Gag | NKIVRMYSP | 271 | 279 | - | X | 4 | 0.72677 | 4.60E-20 |
| Gag | KIVRMYSPT | 272 | 280 | - | X | 4 | 0.72677 | 4.60E-20 |
| Gag | IVRMYSPTS | 273 | 281 | - | X | 4 | 0.72677 | 4.60E-20 |
| Gag | VRMYSPTSI | 274 | 282 | - | X | 4 | 0.72677 | 4.60E-20 |
| Gag | SILDIRQGP | 281 | 289 | X | - | 4 | 0.939702 | 5.41E-43 |
| Gag | ILDIRQGPK | 282 | 290 | X | - | 4 | 0.939702 | 5.41E-43 |
| Gag | RQGPKEPFR | 286 | 294 | X | - | 4 | 0.937911 | 5.41E-43 |
| Gag | QGPKEPFRD | 287 | 295 | X | - | 4 | 0.937911 | 5.41E-43 |
| Gag | GPKEPFRDY | 288 | 296 | X | - | 4 | 0.937911 | 5.41E-43 |
| Gag | PKEPFRDYV | 289 | 297 | X | - | 4 | 0.937911 | 5.41E-43 |
| Gag | NPDCKTILK | 327 | 335 | - | X | 4 | 0.369397 | 4.60E-20 |
| Gag | PDCKTILKA | 328 | 336 | - | X | 4 | 0.369397 | 4.60E-20 |
| Gag | ALGPAATLE | 336 | 344 | - | X | 4 | 0.602748 | 4.60E-20 |
| Gag | LGPAATLEE | 337 | 345 | - | X | 4 | 0.602748 | 4.60E-20 |
| Gag | GPAATLEEM | 338 | 346 | - | X | 4 | 0.602748 | 4.60E-20 |
| Gag | PAATLEEMM | 339 | 347 | - | X | 4 | 0.602748 | 4.60E-20 |
| Gag | AATLEEMMT | 340 | 348 | - | X | 5 | 0.680925 | 1.49E-118 |
| Gag | ATLEEMMTA | 341 | 349 | - | X | 4 | 0.53464 | 4.60E-20 |
| Gag | RVLAEAMSQ | 361 | 369 | X | - | 4 | 0.432532 | 5.41E-43 |
| Gag | VLAEAMSQV | 362 | 370 | X | - | 4 | 0.432532 | 5.41E-43 |
| Gag | QVTNSATIM | 369 | 377 | - | X | 4 | 0.319642 | 4.60E-20 |
| Gag | QVTNSATIM | 369 | 377 | X | - | 4 | 0.603863 | 5.41E-43 |
| Gag | VTNSATIMM | 370 | 378 | - | X | 4 | 0.319642 | 4.60E-20 |
| Gag | NFRNQRKIV | 382 | 390 | X | - | 4 | 0.47802 | 5.41E-43 |
| Nef | PLRPMTYKA | 75 | 83 | X | - | 4 | 0.988186 | 5.41E-43 |
| Nef | RPMTYKAAV | 77 | 85 | X | - | 4 | 0.969978 | 5.41E-43 |
| Nef | PMTYKAAVD | 78 | 86 | X | - | 4 | 0.969978 | 5.41E-43 |
| Nef | AAVDLSHFL | 83 | 91 | - | X | 4 | 0.641248 | 4.60E-20 |
| Nef | AVDLSHFLK | 84 | 92 | - | X | 4 | 0.641248 | 4.60E-20 |
| Nef | VDLSHFLKE | 85 | 93 | - | X | 4 | 0.641248 | 4.60E-20 |
| Nef | DLSHFLKEK | 86 | 94 | - | X | 4 | 0.641248 | 4.60E-20 |
| Nef | KGGLEGLIH | 94 | 102 | - | X | 4 | 0.554809 | 4.60E-20 |
| Nef | LWIYHTQGY | 112 | 120 | X | - | 4 | 0.814014 | 5.41E-43 |
| Nef | WIYHTQGYF | 113 | 121 | X | - | 4 | 0.814014 | 5.41E-43 |
| Nef | TFGWCYKLV | 138 | 146 | - | X | 4 | 0.36137 | 4.60E-20 |
| Nef | FGWCYKLVP | 139 | 147 | - | X | 4 | 0.36137 | 4.60E-20 |
| Nef | GWCYKLVPV | 140 | 148 | - | X | 4 | 0.36137 | 4.60E-20 |
| Nef | WCYKLVPVE | 141 | 149 | - | X | 5 | 0.56095 | 1.49E-118 |
| Nef | CYKLVPVEP | 142 | 150 | - | X | 5 | 0.56095 | 1.49E-118 |
| Nef | YKLVPVEPD | 143 | 151 | - | X | 4 | 0.56095 | 4.60E-20 |
| Nef | KLVPVEPDK | 144 | 152 | - | X | 5 | 0.567381 | 1.49E-118 |
| Nef | LVPVEPDKI | 145 | 153 | - | X | 5 | 0.567381 | 1.49E-118 |
| Nef | VPVEPDKIE | 146 | 154 | - | X | 5 | 0.567381 | 1.49E-118 |
| Nef | PVEPDKIEE | 147 | 155 | - | X | 4 | 0.354372 | 4.60E-20 |
| Nef | VEPDKIEEA | 148 | 156 | - | X | 4 | 0.354372 | 4.60E-20 |
| Nef | EPDKIEEAN | 149 | 157 | - | X | 4 | 0.354372 | 4.60E-20 |
| Nef | PDKIEEANK | 150 | 158 | - | X | 4 | 0.354372 | 4.60E-20 |
| Nef | EVLEWRFDS | 179 | 187 | X | - | 4 | 0.631695 | 5.41E-43 |
| Nef | SRLAFHHVA | 187 | 195 | - | X | 4 | 0.804694 | 4.60E-20 |
| Nef | RLAFHHVAR | 188 | 196 | - | X | 4 | 0.804694 | 4.60E-20 |
| Nef | LAFHHVARE | 189 | 197 | - | X | 4 | 0.804694 | 4.60E-20 |
| Pol | LQGKAREFS | 9 | 17 | - | X | 4 | 0.657457 | 4.60E-20 |
| Pol | NFPQVTLWQ | 55 | 63 | X | - | 4 | 0.58531 | 5.41E-43 |
| Pol | RQYDQILIE | 113 | 121 | X | - | 4 | 0.758626 | 5.41E-43 |
| Pol | QYDQILIEI | 114 | 122 | X | - | 4 | 0.758626 | 5.41E-43 |
| Pol | YDQILIEIC | 115 | 123 | X | - | 4 | 0.758626 | 5.41E-43 |
| Pol | DQILIEICG | 116 | 124 | X | - | 4 | 0.758626 | 5.41E-43 |
| Pol | QILIEICGH | 117 | 125 | X | - | 4 | 0.758626 | 5.41E-43 |
| Pol | ILIEICGHK | 118 | 126 | X | - | 4 | 0.758626 | 5.41E-43 |
| Pol | KAIGTVLVG | 126 | 134 | - | X | 4 | 0.591556 | 4.60E-20 |
| Pol | CTLNFPISP | 151 | 159 | X | - | 4 | 0.704466 | 5.41E-43 |
| Pol | TLNFPISPI | 152 | 160 | X | - | 4 | 0.704466 | 5.41E-43 |
| Pol | LVEICTEME | 189 | 197 | - | X | 4 | 0.350317 | 4.60E-20 |
| Pol | VEICTEMEK | 190 | 198 | - | X | 4 | 0.350317 | 4.60E-20 |
| Pol | EICTEMEKE | 191 | 199 | - | X | 4 | 0.350317 | 4.60E-20 |
| Pol | ICTEMEKEG | 192 | 200 | - | X | 4 | 0.350317 | 4.60E-20 |
| Pol | CTEMEKEGK | 193 | 201 | - | X | 4 | 0.350317 | 4.60E-20 |
| Pol | TEMEKEGKI | 194 | 202 | - | X | 4 | 0.350317 | 4.60E-20 |
| Pol | EMEKEGKIS | 195 | 203 | - | X | 4 | 0.350317 | 4.60E-20 |
| Pol | MEKEGKISK | 196 | 204 | - | X | 4 | 0.350317 | 4.60E-20 |
| Pol | SINNETPGI | 289 | 297 | X | - | 4 | 0.95561 | 5.41E-43 |
| Pol | ETPGIRYQY | 293 | 301 | X | - | 4 | 0.807809 | 5.41E-43 |
| Pol | TPGIRYQYN | 294 | 302 | X | - | 4 | 0.807809 | 5.41E-43 |
| Pol | PGIRYQYNV | 295 | 303 | X | - | 4 | 0.807809 | 5.41E-43 |
| Pol | GIRYQYNVL | 296 | 304 | X | - | 4 | 0.807809 | 5.41E-43 |
| Pol | QNPDIVIYQ | 329 | 337 | X | - | 4 | 0.69129 | 5.41E-43 |
| Pol | NPDIVIYQY | 330 | 338 | X | - | 4 | 0.69129 | 5.41E-43 |
| Pol | PDIVIYQYM | 331 | 339 | X | - | 4 | 0.69129 | 5.41E-43 |
| Pol | DIVIYQYMD | 332 | 340 | X | - | 4 | 0.69129 | 5.41E-43 |
| Pol | IVIYQYMDD | 333 | 341 | X | - | 4 | 0.69129 | 5.41E-43 |
| Pol | VIYQYMDDL | 334 | 342 | X | - | 4 | 0.69129 | 5.41E-43 |
| Pol | IYQYMDDLY | 335 | 343 | X | - | 4 | 0.69129 | 5.41E-43 |
| Pol | ELRQHLLRW | 359 | 367 | X | - | 4 | 0.959075 | 5.41E-43 |
| Pol | LRQHLLRWG | 360 | 368 | X | - | 4 | 0.959075 | 5.41E-43 |
| Pol | RQHLLRWGL | 361 | 369 | X | - | 4 | 0.959075 | 5.41E-43 |
| Pol | QHLLRWGLT | 362 | 370 | X | - | 4 | 0.959075 | 5.41E-43 |
| Pol | HLLRWGLTT | 363 | 371 | X | - | 5 | 0.95926 | 4.48E-226 |
| Pol | LLRWGLTTP | 364 | 372 | X | - | 5 | 0.95926 | 4.48E-226 |
| Pol | LRWGLTTPD | 365 | 373 | X | - | 4 | 0.95926 | 5.41E-43 |
| Pol | PDKKHQKEP | 372 | 380 | X | - | 4 | 0.758757 | 5.41E-43 |
| Pol | DKKHQKEPP | 373 | 381 | X | - | 4 | 0.758757 | 5.41E-43 |
| Pol | QGQGQWTYQ | 487 | 495 | X | - | 4 | 0.930721 | 5.41E-43 |
| Pol | GQGQWTYQI | 488 | 496 | X | - | 4 | 0.930721 | 5.41E-43 |
| Pol | QGQWTYQIY | 489 | 497 | X | - | 4 | 0.930721 | 5.41E-43 |
| Pol | GQWTYQIYQ | 490 | 498 | X | - | 4 | 0.930721 | 5.41E-43 |
| Pol | QWTYQIYQE | 491 | 499 | X | - | 4 | 0.930721 | 5.41E-43 |
| Pol | ESIVIWGKT | 533 | 541 | X | - | 4 | 0.851601 | 5.41E-43 |
| Pol | SIVIWGKTP | 534 | 542 | X | - | 4 | 0.851601 | 5.41E-43 |
| Pol | WGKTPKFKL | 538 | 546 | X | - | 4 | 0.851002 | 5.41E-43 |
| Pol | GKTPKFKLP | 539 | 547 | X | - | 4 | 0.851002 | 5.41E-43 |
| Pol | TPKFKLPIQ | 541 | 549 | X | - | 4 | 0.795025 | 5.41E-43 |
| Pol | PKFKLPIQK | 542 | 550 | X | - | 4 | 0.795025 | 5.41E-43 |
| Pol | QATWIPEWE | 562 | 570 | X | - | 4 | 0.970758 | 5.41E-43 |
| Pol | ATWIPEWEF | 563 | 571 | X | - | 4 | 0.970758 | 5.41E-43 |
| Pol | TWIPEWEFV | 564 | 572 | X | - | 5 | 0.970758 | 4.48E-226 |
| Pol | WIPEWEFVN | 565 | 573 | X | - | 4 | 0.970516 | 5.41E-43 |
| Pol | IPEWEFVNT | 566 | 574 | X | - | 4 | 0.970516 | 5.41E-43 |
| Pol | PEWEFVNTP | 567 | 575 | X | - | 4 | 0.970516 | 5.41E-43 |
| Pol | EWEFVNTPP | 568 | 576 | X | - | 4 | 0.970516 | 5.41E-43 |
| Pol | VESMNKELK | 866 | 874 | - | X | 4 | 0.525545 | 4.60E-20 |
| Pol | ESMNKELKK | 867 | 875 | - | X | 4 | 0.525545 | 4.60E-20 |
| Pol | SMNKELKKI | 868 | 876 | - | X | 4 | 0.525545 | 4.60E-20 |
| Pol | MNKELKKII | 869 | 877 | - | X | 5 | 0.525545 | 1.49E-118 |
| Pol | NKELKKIIG | 870 | 878 | - | X | 5 | 0.525545 | 1.49E-118 |
| Pol | KELKKIIGQ | 871 | 879 | - | X | 5 | 0.525545 | 1.49E-118 |
| Pol | ELKKIIGQV | 872 | 880 | - | X | 5 | 0.525545 | 1.49E-118 |
| Pol | LKKIIGQVR | 873 | 881 | - | X | 4 | 0.350182 | 4.60E-20 |
| Pol | HLKTAVQMA | 886 | 894 | X | - | 4 | 0.941568 | 5.41E-43 |
| Pol | AVFIHNFKR | 894 | 902 | X | - | 4 | 0.931544 | 5.41E-43 |
| Pol | VFIHNFKRK | 895 | 903 | X | - | 4 | 0.931544 | 5.41E-43 |
| Pol | GERIVDIIA | 912 | 920 | - | X | 4 | 0.564283 | 4.60E-20 |
| Pol | ERIVDIIAT | 913 | 921 | - | X | 4 | 0.564283 | 4.60E-20 |
| Pol | RIVDIIATD | 914 | 922 | - | X | 4 | 0.564283 | 4.60E-20 |
| Pol | IVDIIATDI | 915 | 923 | - | X | 4 | 0.564283 | 4.60E-20 |
| Pol | VDIIATDIQ | 916 | 924 | - | X | 4 | 0.564283 | 4.60E-20 |
| Pol | TKELQKQIT | 925 | 933 | X | - | 4 | 0.623973 | 5.41E-43 |
| Pol | KELQKQITK | 926 | 934 | X | - | 4 | 0.623973 | 5.41E-43 |
| Pol | ELQKQITKI | 927 | 935 | X | - | 4 | 0.623973 | 5.41E-43 |
| Pol | IQNFRVYYR | 935 | 943 | - | X | 4 | 0.8569 | 4.60E-20 |
| Pol | QNFRVYYRD | 936 | 944 | - | X | 4 | 0.8569 | 4.60E-20 |
| Pol | NFRVYYRDS | 937 | 945 | - | X | 4 | 0.8569 | 4.60E-20 |
| Pol | FRVYYRDSR | 938 | 946 | - | X | 4 | 0.8569 | 4.60E-20 |
| Pol | RVYYRDSRN | 939 | 947 | - | X | 4 | 0.8569 | 4.60E-20 |
| Rev | SDEELIRTV | 8 | 16 | X | - | 4 | 0.939937 | 5.41E-43 |
| Rev | DEELIRTVR | 9 | 17 | X | - | 4 | 0.939937 | 5.41E-43 |
| Rev | EELIRTVRL | 10 | 18 | X | - | 4 | 0.939937 | 5.41E-43 |
| Rev | ELIRTVRLI | 11 | 19 | X | - | 4 | 0.939937 | 5.41E-43 |
| Rev | RQRQIHSIS | 48 | 56 | X | - | 4 | 0.852286 | 5.41E-43 |
| Rev | QRQIHSISE | 49 | 57 | X | - | 5 | 0.887176 | 4.48E-226 |
| Rev | RQIHSISER | 50 | 58 | X | - | 5 | 0.887176 | 4.48E-226 |
| Rev | QIHSISERI | 51 | 59 | X | - | 5 | 0.887176 | 4.48E-226 |
| Rev | IHSISERIL | 52 | 60 | X | - | 4 | 0.884801 | 5.41E-43 |
| Rev | HSISERILG | 53 | 61 | X | - | 4 | 0.884801 | 5.41E-43 |
| Rev | SISERILGT | 54 | 62 | X | - | 4 | 0.884801 | 5.41E-43 |
| Rev | SERILGTYL | 56 | 64 | X | - | 4 | 0.919434 | 5.41E-43 |
| Rev | ERILGTYLG | 57 | 65 | X | - | 4 | 0.919434 | 5.41E-43 |
| Rev | TYLGRSAEP | 62 | 70 | X | - | 4 | 0.796156 | 5.41E-43 |
| Rev | QLPPLERLT | 74 | 82 | - | X | 4 | 0.822666 | 4.60E-20 |
| Rev | LPPLERLTL | 75 | 83 | - | X | 4 | 0.822666 | 4.60E-20 |
| Rev | PPLERLTLD | 76 | 84 | - | X | 4 | 0.822666 | 4.60E-20 |
| Rev | PLERLTLDC | 77 | 85 | - | X | 4 | 0.822666 | 4.60E-20 |
| Rev | LERLTLDCN | 78 | 86 | - | X | 5 | 0.822666 | 1.49E-118 |
| Rev | ERLTLDCNE | 79 | 87 | - | X | 4 | 0.801489 | 4.60E-20 |
| Rev | RLTLDCNED | 80 | 88 | - | X | 4 | 0.801489 | 4.60E-20 |
| Rev | LTLDCNEDC | 81 | 89 | - | X | 4 | 0.801489 | 4.60E-20 |
| Rev | TLDCNEDCG | 82 | 90 | - | X | 5 | 0.825905 | 1.49E-118 |
| Rev | LDCNEDCGT | 83 | 91 | - | X | 4 | 0.671272 | 4.60E-20 |
| Rev | DCNEDCGTS | 84 | 92 | - | X | 4 | 0.671272 | 4.60E-20 |
| Rev | CNEDCGTSG | 85 | 93 | - | X | 4 | 0.671272 | 4.60E-20 |
| Rev | NEDCGTSGT | 86 | 94 | - | X | 4 | 0.671272 | 4.60E-20 |
| Rev | EDCGTSGTQ | 87 | 95 | - | X | 4 | 0.671272 | 4.60E-20 |
| Rev | QGVGSPQIL | 95 | 103 | X | - | 4 | 0.938573 | 5.41E-43 |
| Rev | GVGSPQILV | 96 | 104 | X | - | 4 | 0.938573 | 5.41E-43 |
| Rev | VGSPQILVE | 97 | 105 | X | - | 4 | 0.938573 | 5.41E-43 |
| Rev | GSPQILVES | 98 | 106 | X | - | 4 | 0.938573 | 5.41E-43 |
| Rev | SPQILVESP | 99 | 107 | X | - | 4 | 0.938573 | 5.41E-43 |
| Rev | PQILVESPT | 100 | 108 | X | - | 4 | 0.938573 | 5.41E-43 |
| Rev | QILVESPTV | 101 | 109 | X | - | 4 | 0.938573 | 5.41E-43 |
| Tat | VDPRLEPWK | 4 | 12 | - | X | 4 | 0.56539 | 4.60E-20 |
| Tat | DPRLEPWKH | 5 | 13 | - | X | 4 | 0.56539 | 4.60E-20 |
| Tat | PRLEPWKHP | 6 | 14 | - | X | 4 | 0.56539 | 4.60E-20 |
| Tat | RLEPWKHPG | 7 | 15 | - | X | 4 | 0.56539 | 4.60E-20 |
| Tat | LEPWKHPGS | 8 | 16 | - | X | 4 | 0.56539 | 4.60E-20 |
| Tat | FHCQVCFIT | 32 | 40 | - | X | 4 | 0.3783 | 4.60E-20 |
| Tat | HCQVCFITK | 33 | 41 | - | X | 4 | 0.3783 | 4.60E-20 |
| Tat | CQVCFITKA | 34 | 42 | - | X | 4 | 0.3783 | 4.60E-20 |
| Tat | QVCFITKAL | 35 | 43 | - | X | 4 | 0.3783 | 4.60E-20 |
| Tat | VCFITKALG | 36 | 44 | - | X | 4 | 0.3783 | 4.60E-20 |
| Tat | CFITKALGI | 37 | 45 | - | X | 4 | 0.3783 | 4.60E-20 |
| Tat | FITKALGIS | 38 | 46 | - | X | 4 | 0.3783 | 4.60E-20 |
| Tat | ITKALGISY | 39 | 47 | - | X | 4 | 0.3783 | 4.60E-20 |
| Tat | TKALGISYG | 40 | 48 | - | X | 4 | 0.556376 | 4.60E-20 |
| Tat | KALGISYGR | 41 | 49 | - | X | 4 | 0.556376 | 4.60E-20 |
| Tat | ALGISYGRK | 42 | 50 | - | X | 4 | 0.556376 | 4.60E-20 |
| Tat | LGISYGRKK | 43 | 51 | - | X | 4 | 0.556376 | 4.60E-20 |
| Vif | DRMRIRTWK | 14 | 22 | - | X | 4 | 0.660791 | 4.60E-20 |
| Vif | RMRIRTWKS | 15 | 23 | - | X | 4 | 0.660791 | 4.60E-20 |
| Vif | MRIRTWKSL | 16 | 24 | - | X | 4 | 0.660791 | 4.60E-20 |
| Vif | RIRTWKSLV | 17 | 25 | - | X | 4 | 0.660791 | 4.60E-20 |
| Vif | SLVKHHMYV | 23 | 31 | X | - | 4 | 0.652127 | 5.41E-43 |
| Vif | LVKHHMYVS | 24 | 32 | X | - | 4 | 0.652127 | 5.41E-43 |
| Vif | MYVSGKARG | 29 | 37 | X | - | 4 | 0.60883 | 5.41E-43 |
| Vif | VITTYWGLH | 65 | 73 | - | X | 4 | 0.368463 | 4.60E-20 |
| Vif | LHTGERDWH | 72 | 80 | - | X | 4 | 0.39657 | 4.60E-20 |
| Vif | HTGERDWHL | 73 | 81 | - | X | 4 | 0.39657 | 4.60E-20 |
| Vif | TGERDWHLG | 74 | 82 | - | X | 4 | 0.39657 | 4.60E-20 |
| Vif | GERDWHLGQ | 75 | 83 | - | X | 4 | 0.39657 | 4.60E-20 |
| Vif | ERDWHLGQG | 76 | 84 | - | X | 4 | 0.39657 | 4.60E-20 |
| Vif | LALAALITP | 148 | 156 | X | - | 4 | 0.779449 | 5.41E-43 |
| Vif | ALAALITPK | 149 | 157 | X | - | 4 | 0.779449 | 5.41E-43 |
| Vif | LAALITPKK | 150 | 158 | X | - | 4 | 0.779449 | 5.41E-43 |
| Vif | AALITPKKI | 151 | 159 | X | - | 5 | 0.863713 | 4.48E-226 |
| Vif | ALITPKKIK | 152 | 160 | X | - | 4 | 0.861489 | 5.41E-43 |
| Vif | LITPKKIKP | 153 | 161 | X | - | 4 | 0.629271 | 5.41E-43 |
| Vif | ITPKKIKPP | 154 | 162 | X | - | 4 | 0.629271 | 5.41E-43 |
| Vif | TPKKIKPPL | 155 | 163 | X | - | 4 | 0.629271 | 5.41E-43 |
| Vif | PKKIKPPLP | 156 | 164 | X | - | 4 | 0.629271 | 5.41E-43 |
| Vif | IKPPLPSVT | 159 | 167 | X | - | 4 | 0.610964 | 5.41E-43 |
| Vif | KPPLPSVTK | 160 | 168 | X | - | 4 | 0.610964 | 5.41E-43 |
| Vif | PPLPSVTKL | 161 | 169 | X | - | 5 | 0.788417 | 4.48E-226 |
| Vif | PLPSVTKLT | 162 | 170 | X | - | 5 | 0.788417 | 4.48E-226 |
| Vif | LPSVTKLTE | 163 | 171 | X | - | 4 | 0.762023 | 5.41E-43 |
| Vif | PSVTKLTED | 164 | 172 | X | - | 4 | 0.762023 | 5.41E-43 |
| Vif | SVTKLTEDR | 165 | 173 | X | - | 4 | 0.762023 | 5.41E-43 |
| Vif | VTKLTEDRW | 166 | 174 | X | - | 4 | 0.868065 | 5.41E-43 |
| Vif | TKLTEDRWN | 167 | 175 | X | - | 4 | 0.868065 | 5.41E-43 |
| Vpr | YGDTWAGVE | 50 | 58 | - | X | 4 | 0.463732 | 4.60E-20 |
| Vpr | GDTWAGVEA | 51 | 59 | - | X | 4 | 0.463732 | 4.60E-20 |
| Vpr | DTWAGVEAI | 52 | 60 | - | X | 4 | 0.463732 | 4.60E-20 |
| Vpr | TWAGVEAII | 53 | 61 | - | X | 4 | 0.463732 | 4.60E-20 |
| Vpr | WAGVEAIIR | 54 | 62 | - | X | 4 | 0.463732 | 4.60E-20 |
| Vpu | KILRQRKID | 32 | 40 | - | X | 4 | 0.501653 | 4.60E-20 |
| Vpu | ILRQRKIDR | 33 | 41 | - | X | 4 | 0.501653 | 4.60E-20 |
| Vpu | LRQRKIDRL | 34 | 42 | X | - | 4 | 0.813061 | 5.41E-43 |
| Vpu | RQRKIDRLI | 35 | 43 | X | - | 4 | 0.813061 | 5.41E-43 |
| Vpu | QRKIDRLID | 36 | 44 | X | - | 4 | 0.813061 | 5.41E-43 |

**Table S12**. Full list of 30 residues long HIV-1 specific regions associated with the clusters predicted by Predivac-3.0 for the Japanese population, including the corresponding protein, sequence, position (start/end), allele restriction (HLA class I or class II), number of epitopes associated with this region, predicted population coverage and statistical significance of each sequence (p-values).

| **Protein** | **Sequence** | **Start** | **End** | **HLA**  **class I** | **HLA**  **Class II** | **Number of**  **epitopes** | **Coverage**  **(%)** | **p-value** |
| --- | --- | --- | --- | --- | --- | --- | --- | --- |
| Env | MVEQMHEDIISLWDQSLKPCVKLTPLCVSL | 100 | 129 | X | - | 5 | 0.925968 | 2.24E-21 |
| Env | VEQMHEDIISLWDQSLKPCVKLTPLCVSLK | 101 | 130 | X | - | 5 | 0.925968 | 2.24E-21 |
| Env | EQMHEDIISLWDQSLKPCVKLTPLCVSLKC | 102 | 131 | X | - | 5 | 0.925968 | 2.24E-21 |
| Env | QMHEDIISLWDQSLKPCVKLTPLCVSLKCT | 103 | 132 | X | - | 5 | 0.925968 | 2.24E-21 |
| Env | NDTNTNSSSGRMIMEKGEIKNCSFNISTSI | 136 | 165 | X | - | 5 | 0.707572 | 2.24E-21 |
| Env | DTNTNSSSGRMIMEKGEIKNCSFNISTSIR | 137 | 166 | X | - | 5 | 0.707572 | 2.24E-21 |
| Env | TNTNSSSGRMIMEKGEIKNCSFNISTSIRG | 138 | 167 | X | - | 5 | 0.707572 | 2.24E-21 |
| Env | NTNSSSGRMIMEKGEIKNCSFNISTSIRGK | 139 | 168 | X | - | 5 | 0.707572 | 2.24E-21 |
| Env | TSIRGKVQKEYAFFYKLDIIPIDNDTTSYK | 163 | 192 | - | X | 5 | 0.810878 | 2.02E-15 |
| Env | SIRGKVQKEYAFFYKLDIIPIDNDTTSYKL | 164 | 193 | - | X | 5 | 0.810878 | 2.02E-15 |
| Env | IRGKVQKEYAFFYKLDIIPIDNDTTSYKLT | 165 | 194 | - | X | 5 | 0.810878 | 2.02E-15 |
| Env | RGKVQKEYAFFYKLDIIPIDNDTTSYKLTS | 166 | 195 | - | X | 5 | 0.810878 | 2.02E-15 |
| Env | GKVQKEYAFFYKLDIIPIDNDTTSYKLTSC | 167 | 196 | - | X | 5 | 0.810878 | 2.02E-15 |
| Env | KVQKEYAFFYKLDIIPIDNDTTSYKLTSCN | 168 | 197 | - | X | 5 | 0.810878 | 2.02E-15 |
| Env | VQKEYAFFYKLDIIPIDNDTTSYKLTSCNT | 169 | 198 | - | X | 5 | 0.810878 | 2.02E-15 |
| Env | QKEYAFFYKLDIIPIDNDTTSYKLTSCNTS | 170 | 199 | - | X | 6 | 0.818755 | 5.09E-97 |
| Env | KEYAFFYKLDIIPIDNDTTSYKLTSCNTSV | 171 | 200 | - | X | 6 | 0.818755 | 5.09E-97 |
| Env | EYAFFYKLDIIPIDNDTTSYKLTSCNTSVI | 172 | 201 | - | X | 7 | 0.818755 | 5.60E-252 |
| Env | YAFFYKLDIIPIDNDTTSYKLTSCNTSVIT | 173 | 202 | - | X | 7 | 0.818755 | 5.60E-252 |
| Env | AFFYKLDIIPIDNDTTSYKLTSCNTSVITQ | 174 | 203 | - | X | 6 | 0.757376 | 5.09E-97 |
| Env | FFYKLDIIPIDNDTTSYKLTSCNTSVITQA | 175 | 204 | - | X | 6 | 0.757376 | 5.09E-97 |
| Env | FYKLDIIPIDNDTTSYKLTSCNTSVITQAC | 176 | 205 | - | X | 6 | 0.757376 | 5.09E-97 |
| Env | YKLDIIPIDNDTTSYKLTSCNTSVITQACP | 177 | 206 | - | X | 6 | 0.757376 | 5.09E-97 |
| Env | KLDIIPIDNDTTSYKLTSCNTSVITQACPK | 178 | 207 | - | X | 5 | 0.592521 | 2.02E-15 |
| Env | LDIIPIDNDTTSYKLTSCNTSVITQACPKV | 179 | 208 | - | X | 6 | 0.728523 | 5.09E-97 |
| Env | DIIPIDNDTTSYKLTSCNTSVITQACPKVS | 180 | 209 | - | X | 6 | 0.728523 | 5.09E-97 |
| Env | IIPIDNDTTSYKLTSCNTSVITQACPKVSF | 181 | 210 | - | X | 6 | 0.728523 | 5.09E-97 |
| Env | IPIDNDTTSYKLTSCNTSVITQACPKVSFE | 182 | 211 | - | X | 5 | 0.525198 | 2.02E-15 |
| Env | SYKLTSCNTSVITQACPKVSFEPIPIHYCA | 190 | 219 | X | - | 5 | 0.958052 | 2.24E-21 |
| Env | YKLTSCNTSVITQACPKVSFEPIPIHYCAP | 191 | 220 | X | - | 5 | 0.958052 | 2.24E-21 |
| Env | KLTSCNTSVITQACPKVSFEPIPIHYCAPA | 192 | 221 | X | - | 6 | 0.964118 | 1.60E-131 |
| Env | LTSCNTSVITQACPKVSFEPIPIHYCAPAG | 193 | 222 | X | - | 5 | 0.885196 | 2.24E-21 |
| Env | TSCNTSVITQACPKVSFEPIPIHYCAPAGF | 194 | 223 | X | - | 5 | 0.885196 | 2.24E-21 |
| Env | SCNTSVITQACPKVSFEPIPIHYCAPAGFA | 195 | 224 | X | - | 5 | 0.885196 | 2.24E-21 |
| Env | CNTSVITQACPKVSFEPIPIHYCAPAGFAI | 196 | 225 | X | - | 5 | 0.885196 | 2.24E-21 |
| Env | NTSVITQACPKVSFEPIPIHYCAPAGFAIL | 197 | 226 | X | - | 5 | 0.885196 | 2.24E-21 |
| Env | TSVITQACPKVSFEPIPIHYCAPAGFAILK | 198 | 227 | X | - | 6 | 0.885196 | 1.60E-131 |
| Env | SVITQACPKVSFEPIPIHYCAPAGFAILKC | 199 | 228 | X | - | 6 | 0.885196 | 1.60E-131 |
| Env | VITQACPKVSFEPIPIHYCAPAGFAILKCN | 200 | 229 | X | - | 5 | 0.882294 | 2.24E-21 |
| Env | ITQACPKVSFEPIPIHYCAPAGFAILKCNN | 201 | 230 | X | - | 5 | 0.882294 | 2.24E-21 |
| Env | TQACPKVSFEPIPIHYCAPAGFAILKCNNK | 202 | 231 | X | - | 6 | 0.884018 | 1.60E-131 |
| Env | QACPKVSFEPIPIHYCAPAGFAILKCNNKT | 203 | 232 | X | - | 6 | 0.884018 | 1.60E-131 |
| Env | ACPKVSFEPIPIHYCAPAGFAILKCNNKTF | 204 | 233 | X | - | 7 | 0.884018 | 0 |
| Env | CPKVSFEPIPIHYCAPAGFAILKCNNKTFN | 205 | 234 | X | - | 7 | 0.884018 | 0 |
| Env | PKVSFEPIPIHYCAPAGFAILKCNNKTFNG | 206 | 235 | X | - | 6 | 0.884018 | 1.60E-131 |
| Env | KVSFEPIPIHYCAPAGFAILKCNNKTFNGT | 207 | 236 | X | - | 6 | 0.884018 | 1.60E-131 |
| Env | VSFEPIPIHYCAPAGFAILKCNNKTFNGTG | 208 | 237 | X | - | 5 | 0.688096 | 2.24E-21 |
| Env | SFEPIPIHYCAPAGFAILKCNNKTFNGTGP | 209 | 238 | X | - | 5 | 0.688096 | 2.24E-21 |
| Env | FEPIPIHYCAPAGFAILKCNNKTFNGTGPC | 210 | 239 | X | - | 5 | 0.688096 | 2.24E-21 |
| Env | EPIPIHYCAPAGFAILKCNNKTFNGTGPCT | 211 | 240 | X | - | 5 | 0.688096 | 2.24E-21 |
| Env | TGPCTNVSTVQCTHGIRPVVSTQLLLNGSL | 236 | 265 | X | - | 5 | 0.919887 | 2.24E-21 |
| Env | GPCTNVSTVQCTHGIRPVVSTQLLLNGSLA | 237 | 266 | X | - | 5 | 0.919887 | 2.24E-21 |
| Env | LAEEEVVIRSVNFTDNAKTIIVQLNTSVEI | 265 | 294 | - | X | 5 | 0.405538 | 2.02E-15 |
| Env | AEEEVVIRSVNFTDNAKTIIVQLNTSVEIN | 266 | 295 | - | X | 5 | 0.405538 | 2.02E-15 |
| Env | EEEVVIRSVNFTDNAKTIIVQLNTSVEINC | 267 | 296 | - | X | 5 | 0.405538 | 2.02E-15 |
| Env | EEVVIRSVNFTDNAKTIIVQLNTSVEINCT | 268 | 297 | - | X | 5 | 0.405538 | 2.02E-15 |
| Env | EVVIRSVNFTDNAKTIIVQLNTSVEINCTR | 269 | 298 | - | X | 5 | 0.405538 | 2.02E-15 |
| Env | VVIRSVNFTDNAKTIIVQLNTSVEINCTRP | 270 | 299 | - | X | 5 | 0.405538 | 2.02E-15 |
| Env | VIRSVNFTDNAKTIIVQLNTSVEINCTRPN | 271 | 300 | - | X | 5 | 0.405538 | 2.02E-15 |
| Env | IRSVNFTDNAKTIIVQLNTSVEINCTRPNN | 272 | 301 | - | X | 5 | 0.405538 | 2.02E-15 |
| Env | RSVNFTDNAKTIIVQLNTSVEINCTRPNNN | 273 | 302 | - | X | 6 | 0.413025 | 5.09E-97 |
| Env | SVNFTDNAKTIIVQLNTSVEINCTRPNNNT | 274 | 303 | - | X | 6 | 0.413025 | 5.09E-97 |
| Env | VNFTDNAKTIIVQLNTSVEINCTRPNNNTR | 275 | 304 | - | X | 6 | 0.413025 | 5.09E-97 |
| Env | NFTDNAKTIIVQLNTSVEINCTRPNNNTRK | 276 | 305 | - | X | 5 | 0.329441 | 2.02E-15 |
| Env | FTDNAKTIIVQLNTSVEINCTRPNNNTRKR | 277 | 306 | - | X | 5 | 0.329441 | 2.02E-15 |
| Env | RIRIQRGPGRAFVTIGKIGNMRQAHCNISR | 306 | 335 | X | - | 5 | 0.866809 | 2.24E-21 |
| Env | IRIQRGPGRAFVTIGKIGNMRQAHCNISRA | 307 | 336 | X | - | 5 | 0.866809 | 2.24E-21 |
| Env | RIQRGPGRAFVTIGKIGNMRQAHCNISRAK | 308 | 337 | X | - | 5 | 0.866809 | 2.24E-21 |
| Env | IQRGPGRAFVTIGKIGNMRQAHCNISRAKW | 309 | 338 | X | - | 5 | 0.866809 | 2.24E-21 |
| Env | GGDPEIVTHSFNCGGEFFYCNSTQLFNSTW | 366 | 395 | X | - | 5 | 0.915859 | 2.24E-21 |
| Env | GDPEIVTHSFNCGGEFFYCNSTQLFNSTWF | 367 | 396 | X | - | 5 | 0.915859 | 2.24E-21 |
| Env | DPEIVTHSFNCGGEFFYCNSTQLFNSTWFN | 368 | 397 | X | - | 5 | 0.915859 | 2.24E-21 |
| Env | IVTHSFNCGGEFFYCNSTQLFNSTWFNSTW | 371 | 400 | X | - | 5 | 0.857048 | 2.24E-21 |
| Env | VTHSFNCGGEFFYCNSTQLFNSTWFNSTWS | 372 | 401 | X | - | 5 | 0.857048 | 2.24E-21 |
| Env | THSFNCGGEFFYCNSTQLFNSTWFNSTWST | 373 | 402 | X | - | 5 | 0.857048 | 2.24E-21 |
| Env | HSFNCGGEFFYCNSTQLFNSTWFNSTWSTE | 374 | 403 | X | - | 5 | 0.857048 | 2.24E-21 |
| Env | ITLPCRIKQIINMWQKVGKAMYAPPISGQI | 414 | 443 | - | X | 5 | 0.66973 | 2.02E-15 |
| Env | TLPCRIKQIINMWQKVGKAMYAPPISGQIR | 415 | 444 | - | X | 5 | 0.66973 | 2.02E-15 |
| Env | LPCRIKQIINMWQKVGKAMYAPPISGQIRC | 416 | 445 | - | X | 5 | 0.66973 | 2.02E-15 |
| Env | PCRIKQIINMWQKVGKAMYAPPISGQIRCS | 417 | 446 | - | X | 5 | 0.66973 | 2.02E-15 |
| Env | CRIKQIINMWQKVGKAMYAPPISGQIRCSS | 418 | 447 | - | X | 6 | 0.66973 | 5.09E-97 |
| Env | RIKQIINMWQKVGKAMYAPPISGQIRCSSN | 419 | 448 | - | X | 6 | 0.66973 | 5.09E-97 |
| Env | IKQIINMWQKVGKAMYAPPISGQIRCSSNI | 420 | 449 | - | X | 6 | 0.66973 | 5.09E-97 |
| Env | KQIINMWQKVGKAMYAPPISGQIRCSSNIT | 421 | 450 | - | X | 5 | 0.384113 | 2.02E-15 |
| Env | QIINMWQKVGKAMYAPPISGQIRCSSNITG | 422 | 451 | - | X | 6 | 0.421869 | 5.09E-97 |
| Env | IINMWQKVGKAMYAPPISGQIRCSSNITGL | 423 | 452 | - | X | 6 | 0.421869 | 5.09E-97 |
| Env | INMWQKVGKAMYAPPISGQIRCSSNITGLL | 424 | 453 | - | X | 6 | 0.421869 | 5.09E-97 |
| Env | NMWQKVGKAMYAPPISGQIRCSSNITGLLL | 425 | 454 | - | X | 5 | 0.391603 | 2.02E-15 |
| Env | MWQKVGKAMYAPPISGQIRCSSNITGLLLT | 426 | 455 | - | X | 5 | 0.391603 | 2.02E-15 |
| Env | MTLTVQARQLLSGIVQQQNNLLRAIEAQQH | 535 | 564 | - | X | 5 | 0.386746 | 2.02E-15 |
| Env | WNASWSNKSLEQIWNHTTWMEWDREINNYT | 610 | 639 | - | X | 5 | 0.956843 | 2.02E-15 |
| Env | NKSLEQIWNHTTWMEWDREINNYTSLIHSL | 616 | 645 | X | - | 5 | 0.9939 | 2.24E-21 |
| Env | KSLEQIWNHTTWMEWDREINNYTSLIHSLI | 617 | 646 | - | X | 5 | 0.933155 | 2.02E-15 |
| Env | KSLEQIWNHTTWMEWDREINNYTSLIHSLI | 617 | 646 | X | - | 5 | 0.9939 | 2.24E-21 |
| Env | SLEQIWNHTTWMEWDREINNYTSLIHSLIE | 618 | 647 | - | X | 5 | 0.933155 | 2.02E-15 |
| Env | SLEQIWNHTTWMEWDREINNYTSLIHSLIE | 618 | 647 | X | - | 5 | 0.9939 | 2.24E-21 |
| Env | LEQIWNHTTWMEWDREINNYTSLIHSLIEE | 619 | 648 | - | X | 5 | 0.933155 | 2.02E-15 |
| Env | LEQIWNHTTWMEWDREINNYTSLIHSLIEE | 619 | 648 | X | - | 5 | 0.9939 | 2.24E-21 |
| Env | EQIWNHTTWMEWDREINNYTSLIHSLIEES | 620 | 649 | X | - | 5 | 0.9939 | 2.24E-21 |
| Env | NHTTWMEWDREINNYTSLIHSLIEESQNQQ | 624 | 653 | - | X | 5 | 0.933155 | 2.02E-15 |
| Env | HTTWMEWDREINNYTSLIHSLIEESQNQQE | 625 | 654 | - | X | 6 | 0.945193 | 5.09E-97 |
| Env | TTWMEWDREINNYTSLIHSLIEESQNQQEK | 626 | 655 | - | X | 6 | 0.945193 | 5.09E-97 |
| Env | TTWMEWDREINNYTSLIHSLIEESQNQQEK | 626 | 655 | X | - | 5 | 0.982455 | 2.24E-21 |
| Env | TWMEWDREINNYTSLIHSLIEESQNQQEKN | 627 | 656 | - | X | 6 | 0.945193 | 5.09E-97 |
| Env | TWMEWDREINNYTSLIHSLIEESQNQQEKN | 627 | 656 | X | - | 5 | 0.982455 | 2.24E-21 |
| Env | WMEWDREINNYTSLIHSLIEESQNQQEKNE | 628 | 657 | - | X | 6 | 0.945193 | 5.09E-97 |
| Env | MEWDREINNYTSLIHSLIEESQNQQEKNEQ | 629 | 658 | - | X | 5 | 0.945193 | 2.02E-15 |
| Env | IVGGLVGLRIVFAVLSIVNRVRQGYSPLSF | 688 | 717 | X | - | 5 | 0.886675 | 2.24E-21 |
| Env | VGGLVGLRIVFAVLSIVNRVRQGYSPLSFQ | 689 | 718 | X | - | 5 | 0.886675 | 2.24E-21 |
| Env | GGLVGLRIVFAVLSIVNRVRQGYSPLSFQT | 690 | 719 | X | - | 5 | 0.886675 | 2.24E-21 |
| Env | GLVGLRIVFAVLSIVNRVRQGYSPLSFQTH | 691 | 720 | X | - | 5 | 0.886675 | 2.24E-21 |
| Env | LVGLRIVFAVLSIVNRVRQGYSPLSFQTHL | 692 | 721 | X | - | 6 | 0.971052 | 1.60E-131 |
| Env | VGLRIVFAVLSIVNRVRQGYSPLSFQTHLP | 693 | 722 | X | - | 6 | 0.971052 | 1.60E-131 |
| Env | GLRIVFAVLSIVNRVRQGYSPLSFQTHLPT | 694 | 723 | X | - | 6 | 0.971052 | 1.60E-131 |
| Env | LRIVFAVLSIVNRVRQGYSPLSFQTHLPTP | 695 | 724 | X | - | 5 | 0.971052 | 2.24E-21 |
| Env | RIVFAVLSIVNRVRQGYSPLSFQTHLPTPR | 696 | 725 | X | - | 5 | 0.971052 | 2.24E-21 |
| Env | LKYWWNLLQYWSQELKNSAVSLLNATAIAV | 793 | 822 | X | - | 5 | 0.73634 | 2.24E-21 |
| Env | KYWWNLLQYWSQELKNSAVSLLNATAIAVA | 794 | 823 | X | - | 5 | 0.73634 | 2.24E-21 |
| Env | NSAVSLLNATAIAVAEGTDRVIEVVQGACR | 809 | 838 | X | - | 5 | 0.578554 | 2.24E-21 |
| Env | SAVSLLNATAIAVAEGTDRVIEVVQGACRA | 810 | 839 | X | - | 5 | 0.578554 | 2.24E-21 |
| Env | AVSLLNATAIAVAEGTDRVIEVVQGACRAI | 811 | 840 | X | - | 5 | 0.578554 | 2.24E-21 |
| Env | VAEGTDRVIEVVQGACRAIRHIPRRIRQGL | 822 | 851 | X | - | 5 | 0.63456 | 2.24E-21 |
| Env | AEGTDRVIEVVQGACRAIRHIPRRIRQGLE | 823 | 852 | X | - | 5 | 0.63456 | 2.24E-21 |
| Env | EGTDRVIEVVQGACRAIRHIPRRIRQGLER | 824 | 853 | X | - | 5 | 0.63456 | 2.24E-21 |
| Env | GTDRVIEVVQGACRAIRHIPRRIRQGLERI | 825 | 854 | X | - | 5 | 0.63456 | 2.24E-21 |
| Gag | DTGHSNQVSQNYPIVQNIQGQMVHQAISPR | 121 | 150 | X | - | 5 | 0.813818 | 2.24E-21 |
| Gag | TGHSNQVSQNYPIVQNIQGQMVHQAISPRT | 122 | 151 | X | - | 5 | 0.813818 | 2.24E-21 |
| Gag | GHSNQVSQNYPIVQNIQGQMVHQAISPRTL | 123 | 152 | X | - | 6 | 0.853372 | 1.60E-131 |
| Gag | HSNQVSQNYPIVQNIQGQMVHQAISPRTLN | 124 | 153 | X | - | 6 | 0.853372 | 1.60E-131 |
| Gag | SNQVSQNYPIVQNIQGQMVHQAISPRTLNA | 125 | 154 | X | - | 5 | 0.580054 | 2.24E-21 |
| Gag | NQVSQNYPIVQNIQGQMVHQAISPRTLNAW | 126 | 155 | X | - | 6 | 0.656251 | 1.60E-131 |
| Gag | QVSQNYPIVQNIQGQMVHQAISPRTLNAWV | 127 | 156 | X | - | 6 | 0.5966 | 1.60E-131 |
| Gag | VSQNYPIVQNIQGQMVHQAISPRTLNAWVK | 128 | 157 | X | - | 6 | 0.5966 | 1.60E-131 |
| Gag | SQNYPIVQNIQGQMVHQAISPRTLNAWVKV | 129 | 158 | X | - | 7 | 0.5966 | 0 |
| Gag | QNYPIVQNIQGQMVHQAISPRTLNAWVKVV | 130 | 159 | X | - | 7 | 0.5966 | 0 |
| Gag | NYPIVQNIQGQMVHQAISPRTLNAWVKVVE | 131 | 160 | X | - | 7 | 0.5966 | 0 |
| Gag | YPIVQNIQGQMVHQAISPRTLNAWVKVVEE | 132 | 161 | X | - | 7 | 0.5966 | 0 |
| Gag | PIVQNIQGQMVHQAISPRTLNAWVKVVEEK | 133 | 162 | X | - | 7 | 0.5966 | 0 |
| Gag | IVQNIQGQMVHQAISPRTLNAWVKVVEEKA | 134 | 163 | X | - | 7 | 0.5966 | 0 |
| Gag | VQNIQGQMVHQAISPRTLNAWVKVVEEKAF | 135 | 164 | X | - | 6 | 0.472494 | 1.60E-131 |
| Gag | QNIQGQMVHQAISPRTLNAWVKVVEEKAFS | 136 | 165 | X | - | 5 | 0.472494 | 2.24E-21 |
| Gag | NIQGQMVHQAISPRTLNAWVKVVEEKAFSP | 137 | 166 | X | - | 5 | 0.472494 | 2.24E-21 |
| Gag | IQGQMVHQAISPRTLNAWVKVVEEKAFSPE | 138 | 167 | X | - | 5 | 0.472494 | 2.24E-21 |
| Gag | QGQMVHQAISPRTLNAWVKVVEEKAFSPEV | 139 | 168 | X | - | 6 | 0.477074 | 1.60E-131 |
| Gag | GQMVHQAISPRTLNAWVKVVEEKAFSPEVI | 140 | 169 | X | - | 6 | 0.477074 | 1.60E-131 |
| Gag | QMVHQAISPRTLNAWVKVVEEKAFSPEVIP | 141 | 170 | X | - | 6 | 0.477074 | 1.60E-131 |
| Gag | MVHQAISPRTLNAWVKVVEEKAFSPEVIPM | 142 | 171 | X | - | 6 | 0.477074 | 1.60E-131 |
| Gag | VHQAISPRTLNAWVKVVEEKAFSPEVIPMF | 143 | 172 | X | - | 5 | 0.438983 | 2.24E-21 |
| Gag | HQAISPRTLNAWVKVVEEKAFSPEVIPMFS | 144 | 173 | X | - | 5 | 0.438983 | 2.24E-21 |
| Gag | AISPRTLNAWVKVVEEKAFSPEVIPMFSAL | 146 | 175 | X | - | 5 | 0.795297 | 2.24E-21 |
| Gag | ISPRTLNAWVKVVEEKAFSPEVIPMFSALS | 147 | 176 | X | - | 5 | 0.795297 | 2.24E-21 |
| Gag | DLNTMLNTVGGHQAAMQMLKETINEEAAEW | 183 | 212 | X | - | 5 | 0.505439 | 2.24E-21 |
| Gag | NTVGGHQAAMQMLKETINEEAAEWDRVHPV | 189 | 218 | X | - | 5 | 0.975924 | 2.24E-21 |
| Gag | HQAAMQMLKETINEEAAEWDRVHPVHAGPI | 194 | 223 | - | X | 5 | 0.618985 | 2.02E-15 |
| Gag | QAAMQMLKETINEEAAEWDRVHPVHAGPIA | 195 | 224 | - | X | 5 | 0.618985 | 2.02E-15 |
| Gag | AAMQMLKETINEEAAEWDRVHPVHAGPIAP | 196 | 225 | - | X | 5 | 0.618985 | 2.02E-15 |
| Gag | AMQMLKETINEEAAEWDRVHPVHAGPIAPG | 197 | 226 | - | X | 5 | 0.618985 | 2.02E-15 |
| Gag | MQMLKETINEEAAEWDRVHPVHAGPIAPGQ | 198 | 227 | - | X | 5 | 0.618985 | 2.02E-15 |
| Gag | QMLKETINEEAAEWDRVHPVHAGPIAPGQM | 199 | 228 | - | X | 5 | 0.618985 | 2.02E-15 |
| Gag | MLKETINEEAAEWDRVHPVHAGPIAPGQMR | 200 | 229 | - | X | 5 | 0.618985 | 2.02E-15 |
| Gag | IPVGEIYKRWIILGLNKIVRMYSPTSILDI | 256 | 285 | - | X | 5 | 0.773479 | 2.02E-15 |
| Gag | PVGEIYKRWIILGLNKIVRMYSPTSILDIR | 257 | 286 | - | X | 5 | 0.773479 | 2.02E-15 |
| Gag | VGEIYKRWIILGLNKIVRMYSPTSILDIRQ | 258 | 287 | - | X | 5 | 0.773479 | 2.02E-15 |
| Gag | RWIILGLNKIVRMYSPTSILDIRQGPKEPF | 264 | 293 | - | X | 5 | 0.773479 | 2.02E-15 |
| Gag | WIILGLNKIVRMYSPTSILDIRQGPKEPFR | 265 | 294 | - | X | 5 | 0.773479 | 2.02E-15 |
| Gag | IILGLNKIVRMYSPTSILDIRQGPKEPFRD | 266 | 295 | - | X | 5 | 0.773479 | 2.02E-15 |
| Gag | ILGLNKIVRMYSPTSILDIRQGPKEPFRDY | 267 | 296 | - | X | 5 | 0.773479 | 2.02E-15 |
| Gag | KIVRMYSPTSILDIRQGPKEPFRDYVDRFY | 272 | 301 | X | - | 5 | 0.939702 | 2.24E-21 |
| Gag | IVRMYSPTSILDIRQGPKEPFRDYVDRFYK | 273 | 302 | X | - | 5 | 0.939702 | 2.24E-21 |
| Gag | VRMYSPTSILDIRQGPKEPFRDYVDRFYKT | 274 | 303 | X | - | 5 | 0.939702 | 2.24E-21 |
| Gag | RMYSPTSILDIRQGPKEPFRDYVDRFYKTL | 275 | 304 | X | - | 5 | 0.939702 | 2.24E-21 |
| Gag | FYKTLRAEQASQEVKNWMTETLLVQNANPD | 300 | 329 | - | X | 5 | 0.690995 | 2.02E-15 |
| Gag | YKTLRAEQASQEVKNWMTETLLVQNANPDC | 301 | 330 | - | X | 5 | 0.693139 | 2.02E-15 |
| Gag | VKNWMTETLLVQNANPDCKTILKALGPAAT | 313 | 342 | - | X | 5 | 0.533264 | 2.02E-15 |
| Gag | KNWMTETLLVQNANPDCKTILKALGPAATL | 314 | 343 | - | X | 5 | 0.533264 | 2.02E-15 |
| Gag | NWMTETLLVQNANPDCKTILKALGPAATLE | 315 | 344 | - | X | 5 | 0.533264 | 2.02E-15 |
| Gag | WMTETLLVQNANPDCKTILKALGPAATLEE | 316 | 345 | - | X | 6 | 0.769948 | 5.09E-97 |
| Gag | MTETLLVQNANPDCKTILKALGPAATLEEM | 317 | 346 | - | X | 5 | 0.651237 | 2.02E-15 |
| Gag | TETLLVQNANPDCKTILKALGPAATLEEMM | 318 | 347 | - | X | 5 | 0.651237 | 2.02E-15 |
| Gag | ETLLVQNANPDCKTILKALGPAATLEEMMT | 319 | 348 | - | X | 5 | 0.651237 | 2.02E-15 |
| Gag | TLLVQNANPDCKTILKALGPAATLEEMMTA | 320 | 349 | - | X | 5 | 0.651237 | 2.02E-15 |
| Gag | LLVQNANPDCKTILKALGPAATLEEMMTAC | 321 | 350 | - | X | 5 | 0.651237 | 2.02E-15 |
| Gag | LVQNANPDCKTILKALGPAATLEEMMTACQ | 322 | 351 | - | X | 5 | 0.645461 | 2.02E-15 |
| Gag | ANPDCKTILKALGPAATLEEMMTACQGVGG | 326 | 355 | - | X | 5 | 0.680925 | 2.02E-15 |
| Gag | NPDCKTILKALGPAATLEEMMTACQGVGGP | 327 | 356 | - | X | 5 | 0.680925 | 2.02E-15 |
| Gag | PDCKTILKALGPAATLEEMMTACQGVGGPG | 328 | 357 | - | X | 5 | 0.680925 | 2.02E-15 |
| Gag | DCKTILKALGPAATLEEMMTACQGVGGPGH | 329 | 358 | - | X | 5 | 0.680925 | 2.02E-15 |
| Gag | CKTILKALGPAATLEEMMTACQGVGGPGHK | 330 | 359 | - | X | 5 | 0.680925 | 2.02E-15 |
| Gag | KTILKALGPAATLEEMMTACQGVGGPGHKA | 331 | 360 | - | X | 5 | 0.680925 | 2.02E-15 |
| Gag | TILKALGPAATLEEMMTACQGVGGPGHKAR | 332 | 361 | - | X | 5 | 0.680925 | 2.02E-15 |
| Gag | ILKALGPAATLEEMMTACQGVGGPGHKARV | 333 | 362 | - | X | 5 | 0.680925 | 2.02E-15 |
| Gag | GGPGHKARVLAEAMSQVTNSATIMMQRGNF | 354 | 383 | X | - | 5 | 0.440024 | 2.24E-21 |
| Gag | GPGHKARVLAEAMSQVTNSATIMMQRGNFR | 355 | 384 | X | - | 6 | 0.611903 | 1.60E-131 |
| Gag | PGHKARVLAEAMSQVTNSATIMMQRGNFRN | 356 | 385 | X | - | 5 | 0.611903 | 2.24E-21 |
| Gag | GHKARVLAEAMSQVTNSATIMMQRGNFRNQ | 357 | 386 | X | - | 5 | 0.611903 | 2.24E-21 |
| Gag | HKARVLAEAMSQVTNSATIMMQRGNFRNQR | 358 | 387 | X | - | 5 | 0.611903 | 2.24E-21 |
| Gag | KARVLAEAMSQVTNSATIMMQRGNFRNQRK | 359 | 388 | X | - | 5 | 0.611903 | 2.24E-21 |
| Gag | VLAEAMSQVTNSATIMMQRGNFRNQRKIVK | 362 | 391 | - | X | 5 | 0.425439 | 2.02E-15 |
| Gag | LAEAMSQVTNSATIMMQRGNFRNQRKIVKC | 363 | 392 | - | X | 5 | 0.425439 | 2.02E-15 |
| Gag | SQVTNSATIMMQRGNFRNQRKIVKCFNCGK | 368 | 397 | X | - | 5 | 0.700811 | 2.24E-21 |
| Nef | QEEEEVGFPVTPQVPLRPMTYKAAVDLSHF | 61 | 90 | X | - | 5 | 0.991449 | 2.24E-21 |
| Nef | EEEEVGFPVTPQVPLRPMTYKAAVDLSHFL | 62 | 91 | X | - | 5 | 0.991449 | 2.24E-21 |
| Nef | EEEVGFPVTPQVPLRPMTYKAAVDLSHFLK | 63 | 92 | X | - | 5 | 0.998915 | 2.24E-21 |
| Nef | EEVGFPVTPQVPLRPMTYKAAVDLSHFLKE | 64 | 93 | X | - | 5 | 0.998915 | 2.24E-21 |
| Nef | EVGFPVTPQVPLRPMTYKAAVDLSHFLKEK | 65 | 94 | X | - | 5 | 0.998915 | 2.24E-21 |
| Nef | VGFPVTPQVPLRPMTYKAAVDLSHFLKEKG | 66 | 95 | X | - | 5 | 0.998915 | 2.24E-21 |
| Nef | GFPVTPQVPLRPMTYKAAVDLSHFLKEKGG | 67 | 96 | X | - | 5 | 0.998915 | 2.24E-21 |
| Nef | FPVTPQVPLRPMTYKAAVDLSHFLKEKGGL | 68 | 97 | X | - | 5 | 0.998915 | 2.24E-21 |
| Nef | TPQVPLRPMTYKAAVDLSHFLKEKGGLEGL | 71 | 100 | X | - | 5 | 0.971016 | 2.24E-21 |
| Nef | MTYKAAVDLSHFLKEKGGLEGLIHSQRRQD | 79 | 108 | - | X | 5 | 0.681779 | 2.02E-15 |
| Nef | TYKAAVDLSHFLKEKGGLEGLIHSQRRQDI | 80 | 109 | - | X | 5 | 0.597224 | 2.02E-15 |
| Nef | YKAAVDLSHFLKEKGGLEGLIHSQRRQDIL | 81 | 110 | - | X | 5 | 0.597224 | 2.02E-15 |
| Nef | QGYFPDWQNYTPGPGVRYPLTFGWCYKLVP | 118 | 147 | - | X | 5 | 0.433051 | 2.02E-15 |
| Nef | GYFPDWQNYTPGPGVRYPLTFGWCYKLVPV | 119 | 148 | - | X | 5 | 0.433051 | 2.02E-15 |
| Nef | YFPDWQNYTPGPGVRYPLTFGWCYKLVPVE | 120 | 149 | - | X | 5 | 0.433051 | 2.02E-15 |
| Nef | WQNYTPGPGVRYPLTFGWCYKLVPVEPDKI | 124 | 153 | - | X | 5 | 0.36913 | 2.02E-15 |
| Nef | QNYTPGPGVRYPLTFGWCYKLVPVEPDKIE | 125 | 154 | - | X | 5 | 0.36913 | 2.02E-15 |
| Nef | NYTPGPGVRYPLTFGWCYKLVPVEPDKIEE | 126 | 155 | - | X | 5 | 0.36913 | 2.02E-15 |
| Nef | YTPGPGVRYPLTFGWCYKLVPVEPDKIEEA | 127 | 156 | - | X | 6 | 0.567381 | 5.09E-97 |
| Nef | TPGPGVRYPLTFGWCYKLVPVEPDKIEEAN | 128 | 157 | - | X | 5 | 0.56095 | 2.02E-15 |
| Nef | PGPGVRYPLTFGWCYKLVPVEPDKIEEANK | 129 | 158 | - | X | 5 | 0.56095 | 2.02E-15 |
| Nef | GPGVRYPLTFGWCYKLVPVEPDKIEEANKG | 130 | 159 | - | X | 6 | 0.567381 | 5.09E-97 |
| Nef | PGVRYPLTFGWCYKLVPVEPDKIEEANKGE | 131 | 160 | - | X | 6 | 0.567381 | 5.09E-97 |
| Nef | GVRYPLTFGWCYKLVPVEPDKIEEANKGEN | 132 | 161 | - | X | 6 | 0.567381 | 5.09E-97 |
| Nef | VRYPLTFGWCYKLVPVEPDKIEEANKGENT | 133 | 162 | - | X | 6 | 0.567381 | 5.09E-97 |
| Nef | RYPLTFGWCYKLVPVEPDKIEEANKGENTS | 134 | 163 | - | X | 6 | 0.567381 | 5.09E-97 |
| Nef | YPLTFGWCYKLVPVEPDKIEEANKGENTSL | 135 | 164 | - | X | 6 | 0.567381 | 5.09E-97 |
| Nef | PLTFGWCYKLVPVEPDKIEEANKGENTSLL | 136 | 165 | - | X | 5 | 0.567381 | 2.02E-15 |
| Nef | LTFGWCYKLVPVEPDKIEEANKGENTSLLH | 137 | 166 | - | X | 5 | 0.567381 | 2.02E-15 |
| Nef | TFGWCYKLVPVEPDKIEEANKGENTSLLHP | 138 | 167 | - | X | 5 | 0.567381 | 2.02E-15 |
| Nef | FGWCYKLVPVEPDKIEEANKGENTSLLHPV | 139 | 168 | - | X | 5 | 0.567381 | 2.02E-15 |
| Nef | DPEREVLEWRFDSRLAFHHVARELHPEYFK | 175 | 204 | X | - | 5 | 0.411196 | 2.24E-21 |
| Pol | ADRQGTVSFNFPQVTLWQRPLVTIKIGGQL | 46 | 75 | X | - | 5 | 0.596147 | 2.24E-21 |
| Pol | DRQGTVSFNFPQVTLWQRPLVTIKIGGQLK | 47 | 76 | X | - | 5 | 0.596147 | 2.24E-21 |
| Pol | RQGTVSFNFPQVTLWQRPLVTIKIGGQLKE | 48 | 77 | X | - | 5 | 0.596147 | 2.24E-21 |
| Pol | KPKMIGGIGGFIKVRQYDQILIEICGHKAI | 99 | 128 | X | - | 5 | 0.905134 | 2.24E-21 |
| Pol | PKMIGGIGGFIKVRQYDQILIEICGHKAIG | 100 | 129 | X | - | 5 | 0.905134 | 2.24E-21 |
| Pol | KMIGGIGGFIKVRQYDQILIEICGHKAIGT | 101 | 130 | X | - | 5 | 0.905134 | 2.24E-21 |
| Pol | FIKVRQYDQILIEICGHKAIGTVLVGPTPV | 109 | 138 | X | - | 5 | 0.80286 | 2.24E-21 |
| Pol | IKVRQYDQILIEICGHKAIGTVLVGPTPVN | 110 | 139 | X | - | 5 | 0.80286 | 2.24E-21 |
| Pol | KVRQYDQILIEICGHKAIGTVLVGPTPVNI | 111 | 140 | X | - | 5 | 0.80286 | 2.24E-21 |
| Pol | QILIEICGHKAIGTVLVGPTPVNIIGRNLL | 117 | 146 | - | X | 5 | 0.672907 | 2.02E-15 |
| Pol | ILIEICGHKAIGTVLVGPTPVNIIGRNLLT | 118 | 147 | - | X | 5 | 0.672907 | 2.02E-15 |
| Pol | LIEICGHKAIGTVLVGPTPVNIIGRNLLTQ | 119 | 148 | - | X | 5 | 0.672907 | 2.02E-15 |
| Pol | TLNFPISPIETVPVKLKPGMDGPKVKQWPL | 152 | 181 | X | - | 5 | 0.94379 | 2.24E-21 |
| Pol | EKIKALVEICTEMEKEGKISKIGPENPYNT | 184 | 213 | - | X | 5 | 0.426711 | 2.02E-15 |
| Pol | KIKALVEICTEMEKEGKISKIGPENPYNTP | 185 | 214 | - | X | 5 | 0.426711 | 2.02E-15 |
| Pol | IKALVEICTEMEKEGKISKIGPENPYNTPV | 186 | 215 | - | X | 5 | 0.426711 | 2.02E-15 |
| Pol | KALVEICTEMEKEGKISKIGPENPYNTPVF | 187 | 216 | - | X | 5 | 0.426711 | 2.02E-15 |
| Pol | ALVEICTEMEKEGKISKIGPENPYNTPVFA | 188 | 217 | - | X | 5 | 0.426711 | 2.02E-15 |
| Pol | LVEICTEMEKEGKISKIGPENPYNTPVFAI | 189 | 218 | - | X | 5 | 0.426711 | 2.02E-15 |
| Pol | FRKYTAFTIPSINNETPGIRYQYNVLPQGW | 279 | 308 | X | - | 5 | 0.955835 | 2.24E-21 |
| Pol | RKYTAFTIPSINNETPGIRYQYNVLPQGWK | 280 | 309 | X | - | 5 | 0.955835 | 2.24E-21 |
| Pol | KYTAFTIPSINNETPGIRYQYNVLPQGWKG | 281 | 310 | X | - | 5 | 0.955835 | 2.24E-21 |
| Pol | YTAFTIPSINNETPGIRYQYNVLPQGWKGS | 282 | 311 | X | - | 5 | 0.955835 | 2.24E-21 |
| Pol | FQSSMTKILEPFRKQNPDIVIYQYMDDLYV | 315 | 344 | X | - | 5 | 0.824867 | 2.24E-21 |
| Pol | QSSMTKILEPFRKQNPDIVIYQYMDDLYVG | 316 | 345 | X | - | 5 | 0.824867 | 2.24E-21 |
| Pol | SSMTKILEPFRKQNPDIVIYQYMDDLYVGS | 317 | 346 | X | - | 5 | 0.824867 | 2.24E-21 |
| Pol | SMTKILEPFRKQNPDIVIYQYMDDLYVGSD | 318 | 347 | X | - | 5 | 0.824867 | 2.24E-21 |
| Pol | MTKILEPFRKQNPDIVIYQYMDDLYVGSDL | 319 | 348 | X | - | 5 | 0.824867 | 2.24E-21 |
| Pol | EIGQHRTKIEELRQHLLRWGLTTPDKKHQK | 349 | 378 | X | - | 5 | 0.95926 | 2.24E-21 |
| Pol | IGQHRTKIEELRQHLLRWGLTTPDKKHQKE | 350 | 379 | X | - | 5 | 0.95926 | 2.24E-21 |
| Pol | GQHRTKIEELRQHLLRWGLTTPDKKHQKEP | 351 | 380 | X | - | 5 | 0.95926 | 2.24E-21 |
| Pol | QHRTKIEELRQHLLRWGLTTPDKKHQKEPP | 352 | 381 | X | - | 5 | 0.95926 | 2.24E-21 |
| Pol | HRTKIEELRQHLLRWGLTTPDKKHQKEPPF | 353 | 382 | X | - | 5 | 0.95926 | 2.24E-21 |
| Pol | RTKIEELRQHLLRWGLTTPDKKHQKEPPFL | 354 | 383 | X | - | 5 | 0.95926 | 2.24E-21 |
| Pol | TKIEELRQHLLRWGLTTPDKKHQKEPPFLW | 355 | 384 | X | - | 6 | 0.959781 | 1.60E-131 |
| Pol | KIEELRQHLLRWGLTTPDKKHQKEPPFLWM | 356 | 385 | X | - | 6 | 0.959781 | 1.60E-131 |
| Pol | IEELRQHLLRWGLTTPDKKHQKEPPFLWMG | 357 | 386 | X | - | 6 | 0.959781 | 1.60E-131 |
| Pol | EELRQHLLRWGLTTPDKKHQKEPPFLWMGY | 358 | 387 | X | - | 6 | 0.961352 | 1.60E-131 |
| Pol | ELRQHLLRWGLTTPDKKHQKEPPFLWMGYE | 359 | 388 | X | - | 5 | 0.941321 | 2.24E-21 |
| Pol | LRQHLLRWGLTTPDKKHQKEPPFLWMGYEL | 360 | 389 | X | - | 5 | 0.941321 | 2.24E-21 |
| Pol | RQHLLRWGLTTPDKKHQKEPPFLWMGYELH | 361 | 390 | X | - | 5 | 0.941321 | 2.24E-21 |
| Pol | LLRWGLTTPDKKHQKEPPFLWMGYELHPDK | 364 | 393 | X | - | 5 | 0.758757 | 2.24E-21 |
| Pol | LRWGLTTPDKKHQKEPPFLWMGYELHPDKW | 365 | 394 | X | - | 5 | 0.758757 | 2.24E-21 |
| Pol | RWGLTTPDKKHQKEPPFLWMGYELHPDKWT | 366 | 395 | X | - | 5 | 0.758757 | 2.24E-21 |
| Pol | DSWTVNDIQKLVGKLNWASQIYPGIKVRQL | 405 | 434 | X | - | 5 | 0.643647 | 2.24E-21 |
| Pol | SWTVNDIQKLVGKLNWASQIYPGIKVRQLC | 406 | 435 | X | - | 5 | 0.643647 | 2.24E-21 |
| Pol | LVGKLNWASQIYPGIKVRQLCKLLRGTKAL | 415 | 444 | X | - | 5 | 0.227045 | 2.24E-21 |
| Pol | VGKLNWASQIYPGIKVRQLCKLLRGTKALT | 416 | 445 | X | - | 5 | 0.227045 | 2.24E-21 |
| Pol | GKLNWASQIYPGIKVRQLCKLLRGTKALTE | 417 | 446 | X | - | 5 | 0.227045 | 2.24E-21 |
| Pol | KLNWASQIYPGIKVRQLCKLLRGTKALTEV | 418 | 447 | X | - | 5 | 0.227045 | 2.24E-21 |
| Pol | YYDPSKDLIAEIQKQGQGQWTYQIYQEPFK | 473 | 502 | X | - | 5 | 0.930721 | 2.24E-21 |
| Pol | TEAVQKITTESIVIWGKTPKFKLPIQKETW | 524 | 553 | X | - | 5 | 0.853005 | 2.24E-21 |
| Pol | EAVQKITTESIVIWGKTPKFKLPIQKETWE | 525 | 554 | X | - | 5 | 0.853005 | 2.24E-21 |
| Pol | AVQKITTESIVIWGKTPKFKLPIQKETWET | 526 | 555 | X | - | 5 | 0.853005 | 2.24E-21 |
| Pol | VQKITTESIVIWGKTPKFKLPIQKETWETW | 527 | 556 | X | - | 6 | 0.880891 | 1.60E-131 |
| Pol | QKITTESIVIWGKTPKFKLPIQKETWETWW | 528 | 557 | X | - | 5 | 0.879087 | 2.24E-21 |
| Pol | KITTESIVIWGKTPKFKLPIQKETWETWWT | 529 | 558 | X | - | 5 | 0.879087 | 2.24E-21 |
| Pol | ITTESIVIWGKTPKFKLPIQKETWETWWTE | 530 | 559 | X | - | 5 | 0.879087 | 2.24E-21 |
| Pol | TTESIVIWGKTPKFKLPIQKETWETWWTEY | 531 | 560 | X | - | 5 | 0.879087 | 2.24E-21 |
| Pol | TESIVIWGKTPKFKLPIQKETWETWWTEYW | 532 | 561 | X | - | 5 | 0.879087 | 2.24E-21 |
| Pol | KTPKFKLPIQKETWETWWTEYWQATWIPEW | 540 | 569 | X | - | 5 | 0.856529 | 2.24E-21 |
| Pol | PKFKLPIQKETWETWWTEYWQATWIPEWEF | 542 | 571 | X | - | 5 | 0.795135 | 2.24E-21 |
| Pol | KFKLPIQKETWETWWTEYWQATWIPEWEFV | 543 | 572 | X | - | 5 | 0.795135 | 2.24E-21 |
| Pol | FKLPIQKETWETWWTEYWQATWIPEWEFVN | 544 | 573 | X | - | 5 | 0.795135 | 2.24E-21 |
| Pol | KLPIQKETWETWWTEYWQATWIPEWEFVNT | 545 | 574 | X | - | 5 | 0.795135 | 2.24E-21 |
| Pol | IQKETWETWWTEYWQATWIPEWEFVNTPPL | 548 | 577 | X | - | 5 | 0.972125 | 2.24E-21 |
| Pol | KETWETWWTEYWQATWIPEWEFVNTPPLVK | 550 | 579 | X | - | 5 | 0.970758 | 2.24E-21 |
| Pol | ETWETWWTEYWQATWIPEWEFVNTPPLVKL | 551 | 580 | X | - | 5 | 0.970758 | 2.24E-21 |
| Pol | TWETWWTEYWQATWIPEWEFVNTPPLVKLW | 552 | 581 | X | - | 5 | 0.970758 | 2.24E-21 |
| Pol | WETWWTEYWQATWIPEWEFVNTPPLVKLWY | 553 | 582 | X | - | 5 | 0.970758 | 2.24E-21 |
| Pol | ETWWTEYWQATWIPEWEFVNTPPLVKLWYQ | 554 | 583 | X | - | 5 | 0.970758 | 2.24E-21 |
| Pol | TWWTEYWQATWIPEWEFVNTPPLVKLWYQL | 555 | 584 | X | - | 5 | 0.970758 | 2.24E-21 |
| Pol | WWTEYWQATWIPEWEFVNTPPLVKLWYQLE | 556 | 585 | X | - | 5 | 0.970758 | 2.24E-21 |
| Pol | WTEYWQATWIPEWEFVNTPPLVKLWYQLEK | 557 | 586 | X | - | 5 | 0.970758 | 2.24E-21 |
| Pol | PLVKLWYQLEKEPIVGAETFYVDGAANRET | 576 | 605 | - | X | 5 | 0.747685 | 2.02E-15 |
| Pol | LVKLWYQLEKEPIVGAETFYVDGAANRETK | 577 | 606 | - | X | 5 | 0.747685 | 2.02E-15 |
| Pol | VKLWYQLEKEPIVGAETFYVDGAANRETKL | 578 | 607 | - | X | 5 | 0.747685 | 2.02E-15 |
| Pol | KLWYQLEKEPIVGAETFYVDGAANRETKLG | 579 | 608 | - | X | 5 | 0.747685 | 2.02E-15 |
| Pol | LWYQLEKEPIVGAETFYVDGAANRETKLGK | 580 | 609 | - | X | 5 | 0.747685 | 2.02E-15 |
| Pol | WYQLEKEPIVGAETFYVDGAANRETKLGKA | 581 | 610 | - | X | 5 | 0.747685 | 2.02E-15 |
| Pol | GIPYNPQSQGVVESMNKELKKIIGQVRDQA | 855 | 884 | - | X | 5 | 0.525545 | 2.02E-15 |
| Pol | IPYNPQSQGVVESMNKELKKIIGQVRDQAE | 856 | 885 | - | X | 5 | 0.525545 | 2.02E-15 |
| Pol | PYNPQSQGVVESMNKELKKIIGQVRDQAEH | 857 | 886 | - | X | 5 | 0.525545 | 2.02E-15 |
| Pol | YNPQSQGVVESMNKELKKIIGQVRDQAEHL | 858 | 887 | - | X | 5 | 0.525545 | 2.02E-15 |
| Pol | NPQSQGVVESMNKELKKIIGQVRDQAEHLK | 859 | 888 | - | X | 5 | 0.525545 | 2.02E-15 |
| Pol | PQSQGVVESMNKELKKIIGQVRDQAEHLKT | 860 | 889 | - | X | 5 | 0.525545 | 2.02E-15 |
| Pol | QSQGVVESMNKELKKIIGQVRDQAEHLKTA | 861 | 890 | - | X | 5 | 0.525545 | 2.02E-15 |
| Pol | SQGVVESMNKELKKIIGQVRDQAEHLKTAV | 862 | 891 | - | X | 5 | 0.525545 | 2.02E-15 |
| Pol | QGVVESMNKELKKIIGQVRDQAEHLKTAVQ | 863 | 892 | - | X | 5 | 0.525545 | 2.02E-15 |
| Pol | GVVESMNKELKKIIGQVRDQAEHLKTAVQM | 864 | 893 | - | X | 5 | 0.525545 | 2.02E-15 |
| Pol | VVESMNKELKKIIGQVRDQAEHLKTAVQMA | 865 | 894 | - | X | 5 | 0.525545 | 2.02E-15 |
| Pol | ELKKIIGQVRDQAEHLKTAVQMAVFIHNFK | 872 | 901 | X | - | 5 | 0.941848 | 2.24E-21 |
| Pol | ATDIQTKELQKQITKIQNFRVYYRDSRNPL | 920 | 949 | X | - | 5 | 0.623973 | 2.24E-21 |
| Pol | KELQKQITKIQNFRVYYRDSRNPLWKGPAK | 926 | 955 | X | - | 5 | 0.622295 | 2.24E-21 |
| Rev | PNPEGTRQARRNRRRRWRERQRQIHSISER | 29 | 58 | X | - | 5 | 0.633281 | 2.24E-21 |
| Rev | NPEGTRQARRNRRRRWRERQRQIHSISERI | 30 | 59 | X | - | 5 | 0.633281 | 2.24E-21 |
| Rev | TRQARRNRRRRWRERQRQIHSISERILGTY | 34 | 63 | X | - | 5 | 0.852286 | 2.24E-21 |
| Rev | RQARRNRRRRWRERQRQIHSISERILGTYL | 35 | 64 | X | - | 6 | 0.887176 | 1.60E-131 |
| Rev | QARRNRRRRWRERQRQIHSISERILGTYLG | 36 | 65 | X | - | 5 | 0.887176 | 2.24E-21 |
| Rev | ARRNRRRRWRERQRQIHSISERILGTYLGR | 37 | 66 | X | - | 5 | 0.887176 | 2.24E-21 |
| Rev | RRNRRRRWRERQRQIHSISERILGTYLGRS | 38 | 67 | X | - | 5 | 0.887176 | 2.24E-21 |
| Rev | RNRRRRWRERQRQIHSISERILGTYLGRSA | 39 | 68 | X | - | 5 | 0.887176 | 2.24E-21 |
| Rev | NRRRRWRERQRQIHSISERILGTYLGRSAE | 40 | 69 | X | - | 5 | 0.887176 | 2.24E-21 |
| Rev | RRRRWRERQRQIHSISERILGTYLGRSAEP | 41 | 70 | X | - | 5 | 0.887176 | 2.24E-21 |
| Rev | RRRWRERQRQIHSISERILGTYLGRSAEPV | 42 | 71 | X | - | 6 | 0.952353 | 1.60E-131 |
| Rev | RRWRERQRQIHSISERILGTYLGRSAEPVP | 43 | 72 | X | - | 6 | 0.952353 | 1.60E-131 |
| Rev | RWRERQRQIHSISERILGTYLGRSAEPVPL | 44 | 73 | X | - | 6 | 0.952353 | 1.60E-131 |
| Rev | WRERQRQIHSISERILGTYLGRSAEPVPLQ | 45 | 74 | X | - | 5 | 0.950806 | 2.24E-21 |
| Rev | RERQRQIHSISERILGTYLGRSAEPVPLQL | 46 | 75 | X | - | 5 | 0.950806 | 2.24E-21 |
| Rev | ERQRQIHSISERILGTYLGRSAEPVPLQLP | 47 | 76 | X | - | 5 | 0.950806 | 2.24E-21 |
| Rev | RQRQIHSISERILGTYLGRSAEPVPLQLPP | 48 | 77 | X | - | 5 | 0.923153 | 2.24E-21 |
| Rev | QRQIHSISERILGTYLGRSAEPVPLQLPPL | 49 | 78 | X | - | 5 | 0.923153 | 2.24E-21 |
| Rev | RQIHSISERILGTYLGRSAEPVPLQLPPLE | 50 | 79 | X | - | 5 | 0.923153 | 2.24E-21 |
| Rev | SISERILGTYLGRSAEPVPLQLPPLERLTL | 54 | 83 | X | - | 5 | 0.905196 | 2.24E-21 |
| Rev | ISERILGTYLGRSAEPVPLQLPPLERLTLD | 55 | 84 | X | - | 5 | 0.905196 | 2.24E-21 |
| Rev | ILGTYLGRSAEPVPLQLPPLERLTLDCNED | 59 | 88 | - | X | 5 | 0.610961 | 2.02E-15 |
| Rev | LGTYLGRSAEPVPLQLPPLERLTLDCNEDC | 60 | 89 | - | X | 6 | 0.918203 | 5.09E-97 |
| Rev | GTYLGRSAEPVPLQLPPLERLTLDCNEDCG | 61 | 90 | - | X | 5 | 0.918203 | 2.02E-15 |
| Rev | TYLGRSAEPVPLQLPPLERLTLDCNEDCGT | 62 | 91 | - | X | 5 | 0.918203 | 2.02E-15 |
| Rev | YLGRSAEPVPLQLPPLERLTLDCNEDCGTS | 63 | 92 | - | X | 5 | 0.918203 | 2.02E-15 |
| Rev | LGRSAEPVPLQLPPLERLTLDCNEDCGTSG | 64 | 93 | - | X | 5 | 0.822666 | 2.02E-15 |
| Rev | GRSAEPVPLQLPPLERLTLDCNEDCGTSGT | 65 | 94 | - | X | 5 | 0.822666 | 2.02E-15 |
| Rev | RSAEPVPLQLPPLERLTLDCNEDCGTSGTQ | 66 | 95 | - | X | 5 | 0.822666 | 2.02E-15 |
| Rev | SAEPVPLQLPPLERLTLDCNEDCGTSGTQG | 67 | 96 | - | X | 5 | 0.822666 | 2.02E-15 |
| Rev | AEPVPLQLPPLERLTLDCNEDCGTSGTQGV | 68 | 97 | - | X | 6 | 0.845699 | 5.09E-97 |
| Rev | EPVPLQLPPLERLTLDCNEDCGTSGTQGVG | 69 | 98 | - | X | 6 | 0.845699 | 5.09E-97 |
| Rev | PVPLQLPPLERLTLDCNEDCGTSGTQGVGS | 70 | 99 | - | X | 6 | 0.845699 | 5.09E-97 |
| Rev | VPLQLPPLERLTLDCNEDCGTSGTQGVGSP | 71 | 100 | - | X | 6 | 0.845699 | 5.09E-97 |
| Rev | PLQLPPLERLTLDCNEDCGTSGTQGVGSPQ | 72 | 101 | - | X | 5 | 0.825905 | 2.02E-15 |
| Rev | LQLPPLERLTLDCNEDCGTSGTQGVGSPQI | 73 | 102 | - | X | 5 | 0.825905 | 2.02E-15 |
| Rev | QLPPLERLTLDCNEDCGTSGTQGVGSPQIL | 74 | 103 | - | X | 5 | 0.825905 | 2.02E-15 |
| Rev | LPPLERLTLDCNEDCGTSGTQGVGSPQILV | 75 | 104 | - | X | 5 | 0.825905 | 2.02E-15 |
| Tat | YCKKCCFHCQVCFITKALGISYGRKKRRQR | 26 | 55 | - | X | 5 | 0.629281 | 2.02E-15 |
| Tat | CKKCCFHCQVCFITKALGISYGRKKRRQRR | 27 | 56 | - | X | 5 | 0.629281 | 2.02E-15 |
| Tat | KKCCFHCQVCFITKALGISYGRKKRRQRRR | 28 | 57 | - | X | 5 | 0.629281 | 2.02E-15 |
| Tat | KCCFHCQVCFITKALGISYGRKKRRQRRRA | 29 | 58 | - | X | 5 | 0.629281 | 2.02E-15 |
| Tat | CCFHCQVCFITKALGISYGRKKRRQRRRAH | 30 | 59 | - | X | 5 | 0.629281 | 2.02E-15 |
| Tat | CFHCQVCFITKALGISYGRKKRRQRRRAHQ | 31 | 60 | - | X | 5 | 0.629281 | 2.02E-15 |
| Tat | FHCQVCFITKALGISYGRKKRRQRRRAHQN | 32 | 61 | - | X | 5 | 0.629281 | 2.02E-15 |
| Vif | MIVWQVDRMRIRTWKSLVKHHMYVSGKARG | 8 | 37 | - | X | 5 | 0.848718 | 2.02E-15 |
| Vif | IVWQVDRMRIRTWKSLVKHHMYVSGKARGW | 9 | 38 | - | X | 5 | 0.848718 | 2.02E-15 |
| Vif | VWQVDRMRIRTWKSLVKHHMYVSGKARGWF | 10 | 39 | - | X | 5 | 0.848718 | 2.02E-15 |
| Vif | RMRIRTWKSLVKHHMYVSGKARGWFYRHHY | 15 | 44 | X | - | 5 | 0.660712 | 2.24E-21 |
| Vif | MRIRTWKSLVKHHMYVSGKARGWFYRHHYE | 16 | 45 | X | - | 5 | 0.660712 | 2.24E-21 |
| Vif | RIRTWKSLVKHHMYVSGKARGWFYRHHYES | 17 | 46 | X | - | 5 | 0.660712 | 2.24E-21 |
| Vif | KSLVKHHMYVSGKARGWFYRHHYESPHPRI | 22 | 51 | X | - | 5 | 0.611136 | 2.24E-21 |
| Vif | PLGDARLVITTYWGLHTGERDWHLGQGVSI | 58 | 87 | - | X | 5 | 0.446626 | 2.02E-15 |
| Vif | LVITTYWGLHTGERDWHLGQGVSIEWRKKR | 64 | 93 | - | X | 5 | 0.6056 | 2.02E-15 |
| Vif | VITTYWGLHTGERDWHLGQGVSIEWRKKRY | 65 | 94 | - | X | 5 | 0.6056 | 2.02E-15 |
| Vif | ITTYWGLHTGERDWHLGQGVSIEWRKKRYS | 66 | 95 | - | X | 5 | 0.6056 | 2.02E-15 |
| Vif | TTYWGLHTGERDWHLGQGVSIEWRKKRYST | 67 | 96 | - | X | 5 | 0.6056 | 2.02E-15 |
| Vif | TYWGLHTGERDWHLGQGVSIEWRKKRYSTQ | 68 | 97 | - | X | 5 | 0.6056 | 2.02E-15 |
| Vif | YWGLHTGERDWHLGQGVSIEWRKKRYSTQV | 69 | 98 | - | X | 5 | 0.6056 | 2.02E-15 |
| Vif | AGHNKVGSLQYLALAALITPKKIKPPLPSV | 137 | 166 | X | - | 5 | 0.863713 | 2.24E-21 |
| Vif | GHNKVGSLQYLALAALITPKKIKPPLPSVT | 138 | 167 | X | - | 5 | 0.863713 | 2.24E-21 |
| Vif | HNKVGSLQYLALAALITPKKIKPPLPSVTK | 139 | 168 | X | - | 6 | 0.905484 | 1.60E-131 |
| Vif | NKVGSLQYLALAALITPKKIKPPLPSVTKL | 140 | 169 | X | - | 6 | 0.905484 | 1.60E-131 |
| Vif | KVGSLQYLALAALITPKKIKPPLPSVTKLT | 141 | 170 | X | - | 6 | 0.905484 | 1.60E-131 |
| Vif | VGSLQYLALAALITPKKIKPPLPSVTKLTE | 142 | 171 | X | - | 6 | 0.905484 | 1.60E-131 |
| Vif | GSLQYLALAALITPKKIKPPLPSVTKLTED | 143 | 172 | X | - | 6 | 0.905484 | 1.60E-131 |
| Vif | SLQYLALAALITPKKIKPPLPSVTKLTEDR | 144 | 173 | X | - | 6 | 0.905484 | 1.60E-131 |
| Vif | LQYLALAALITPKKIKPPLPSVTKLTEDRW | 145 | 174 | X | - | 6 | 0.903643 | 1.60E-131 |
| Vif | QYLALAALITPKKIKPPLPSVTKLTEDRWN | 146 | 175 | X | - | 5 | 0.629271 | 2.24E-21 |
| Vif | YLALAALITPKKIKPPLPSVTKLTEDRWNK | 147 | 176 | X | - | 6 | 0.790569 | 1.60E-131 |
| Vif | LALAALITPKKIKPPLPSVTKLTEDRWNKP | 148 | 177 | X | - | 6 | 0.790569 | 1.60E-131 |
| Vif | ALAALITPKKIKPPLPSVTKLTEDRWNKPQ | 149 | 178 | X | - | 6 | 0.790569 | 1.60E-131 |
| Vif | LAALITPKKIKPPLPSVTKLTEDRWNKPQK | 150 | 179 | X | - | 5 | 0.788417 | 2.24E-21 |
| Vif | AALITPKKIKPPLPSVTKLTEDRWNKPQKT | 151 | 180 | X | - | 5 | 0.788417 | 2.24E-21 |
| Vif | ALITPKKIKPPLPSVTKLTEDRWNKPQKTK | 152 | 181 | X | - | 6 | 0.978665 | 1.60E-131 |
| Vif | LITPKKIKPPLPSVTKLTEDRWNKPQKTKG | 153 | 182 | X | - | 6 | 0.978665 | 1.60E-131 |
| Vif | ITPKKIKPPLPSVTKLTEDRWNKPQKTKGH | 154 | 183 | X | - | 6 | 0.978665 | 1.60E-131 |
| Vif | TPKKIKPPLPSVTKLTEDRWNKPQKTKGHR | 155 | 184 | X | - | 6 | 0.978665 | 1.60E-131 |
| Vif | PKKIKPPLPSVTKLTEDRWNKPQKTKGHRG | 156 | 185 | X | - | 5 | 0.976004 | 2.24E-21 |
| Vif | KKIKPPLPSVTKLTEDRWNKPQKTKGHRGS | 157 | 186 | X | - | 5 | 0.976004 | 2.24E-21 |
| Vif | KIKPPLPSVTKLTEDRWNKPQKTKGHRGSH | 158 | 187 | X | - | 5 | 0.976004 | 2.24E-21 |
| Vpr | HFPRIWLHGLGQHIYETYGDTWAGVEAIIR | 33 | 62 | - | X | 5 | 0.661964 | 2.02E-15 |
| Vpr | FPRIWLHGLGQHIYETYGDTWAGVEAIIRI | 34 | 63 | - | X | 5 | 0.661964 | 2.02E-15 |
| Vpr | RIWLHGLGQHIYETYGDTWAGVEAIIRILQ | 36 | 65 | - | X | 5 | 0.54217 | 2.02E-15 |
| Vpr | IWLHGLGQHIYETYGDTWAGVEAIIRILQQ | 37 | 66 | - | X | 5 | 0.54217 | 2.02E-15 |
| Vpr | WLHGLGQHIYETYGDTWAGVEAIIRILQQL | 38 | 67 | - | X | 5 | 0.54217 | 2.02E-15 |
| Vpu | IIAIVVWSIVIIEYRKILRQRKIDRLIDRL | 17 | 46 | X | - | 5 | 0.951011 | 2.24E-21 |
| Vpu | IVVWSIVIIEYRKILRQRKIDRLIDRLIER | 20 | 49 | X | - | 5 | 0.813929 | 2.24E-21 |
| Vpu | VVWSIVIIEYRKILRQRKIDRLIDRLIERA | 21 | 50 | X | - | 5 | 0.813929 | 2.24E-21 |
| Vpu | VWSIVIIEYRKILRQRKIDRLIDRLIERAE | 22 | 51 | X | - | 5 | 0.813929 | 2.24E-21 |

**Table S13**. Optimal combinations of CD8+ and CD4+ T-cell epitopes predicted by Predivac-3.0 in the SARS-CoV-2 spike glycoprotein at each generation of the genetic algorithm (GA) to maximize population coverage in the United Kingdom, South Africa, Brazil and Japan (using the Epitope Optimization mode).

| **GA generation** | **T-cell epitopes** | **Amino  acid position** | **Individual  population  coverage** | **Combined population coverage** |
| --- | --- | --- | --- | --- |
|  |  |  |  |  |
| **United Kingdom** | | | | |
| **CD8+ T-cell epitopes** | | | | |
| 1 | ESNKKFLPF | 554-562 | 0.869 | **0.869** |
| 2 | KIYSKHTPI | 202-210 | 0.762 | **0.992** |
|  | RVDFCGKGY | 1039-1047 | 0.821 |  |
| **CD4+ T-cell epitopes** | | | | |
| 1 | YECDIPIGA | 660-668 | 0.638 | **0.638** |
| 2 | YECDIPIGA | 660-668 | 0.638 | **0.889** |
|  | VKQLSSNFG | 963-971 | 0.464 |  |
| 3 | YECDIPIGA | 660-668 | 0.638 | **0.968** |
|  | VKQLSSNFG | 963-971 | 0.464 |  |
|  | YGDCLGDIA | 837-845 | 0.485 |  |
| 4 | YHKNNKSWM | 145-153 | 0.283 | **0.999** |
|  | FPQSAPHGV | 1052-1060 | 0.449 |  |
|  | VKQLSSNFG | 963-971 | 0.464 |  |
|  | ICGDSTECS | 742-750 | 0.567 |  |
| **South Africa** | | | | |
| **CD8+ T-cell epitopes** | | | | |
| 1 | KQIYKTPPI | 786-794 | 0.885 | **0.885** |
| 2 | KQIYKTPPI | 786-794 | 0.885 | **0.981** |
|  | KSNLKPFER | 458-466 | 0.633 |  |
| 3 | KQIYKTPPI | 786-794 | 0.885 | **1** |
|  | ESNKKFLPF | 554-562 | 0.861 |  |
|  | MIAQYTSAL | 869-877 | 0.499 |  |
| **CD4+ T-cell epitopes** | | | | |
| 1 | MESEFRVYS | 153-161 | **0.567** | **0.567** |
| 2 | FQTLLALHR | 238-246 | **0.298** | **0.715** |
|  | FERDISTEI | 464-472 | **0.516** |  |
| 3 | FQTLLALHR | 238-246 | **0.298** | **0.724** |
|  | VEQDKNTQE | 772-780 | **0.516** |  |
|  | FNSAIGKIQ | 927-935 | **0.026** |  |
| 4 | FQTLLALHR | 238-246 | 0.298 | **0.724** |
|  | YECDIPIGA | 660-668 | 0.516 |  |
|  | FKNHTSPDV | 1156-1164 | 0.0178 |  |
|  | FGAISSVLN | 970-978 | 0.008 |  |
| **Brazil** | | | | |
| **CD8+ T-cell epitopes** | | | | |
| 1 | KQIYKTPPI | 786-794 | 0.906 | **0.906** |
| 2 | KQIYKTPPI | 786-794 | 0.906 | **0.992** |
|  | IPFAMQMAY | 896-904 | 0.672 |  |
| **CD4+ T-cell epitopes** | | | | |
| 1 | YSVLYNSAS | 365-373 | 0.766 | **0.766** |
| 2 | YSVLYNSAS | 365-373 | 0.766 | **0.936** |
|  | VKQLSSNFG | 963-971 | 0.408 |  |
| 3 | YSVLYNSAS | 365-373 | 0.766 | **0.979** |
|  | VKQLSSNFG | 963-971 | 0.408 |  |
|  | ICHDGKAHF | 1081-1089 | 0.217 |  |
| 4 | YSVLYNSAS | 365-373 | 0.766 | **0.992** |
|  | VKQLSSNFG | 963-971 | 0.408 |  |
|  | FKNHTSPDV | 1156-1164 | 0.179 |  |
|  | FDEDDSEPV | 1256-1264 | 0.411 |  |
| **Japan** | | | | |
| **CD8+ T-cell epitopes** | | | | |
| 1 | ESNKKFLPF | 554-562 | **0.938** | **0.938** |
| 2 | ESNKKFLPF | 554-562 | 0.938 | **0.999** |
|  | KQIYKTPPI | 786-794 | 0.909 |  |
| **CD4+ T-cell epitopes** | | | | |
| 1 | YSVLYNSAS | 365-373 | 0.727 | **0.727** |
| 2 | YSVLYNSAS | 365-373 | 0.727 | **0.89** |
|  | VEQDKNTQE | 772-780 | 0.346 |  |
| 3 | YSVLYNSAS | 365-373 | 0.727 | **0.984** |
|  | MESEFRVYS | 153-161 | 0.574 |  |
|  | IAQYTSALL | 870-878 | 0.316 |  |
| 4 | YSVLYNSAS | 365-373 | 0.727 | **0.994** |
|  | MESEFRVYS | 153-161 | 0.574 |  |
|  | IAQYTSALL | 870-878 | 0.316 |  |
|  | LYENQKLIA | 916-924 | 0.231 |  |

**Table S14**. Putative T-cell epitope clusters predicted by Predivac-3.0 in the spike glycoprotein of SARS-CoV-2 when targeting the population of the United Kingdom. In each cluster, the core region of overlapped T-cell epitopes is highlighted in bold/underscored, reporting the amino acid position in the protein, the population coverage predicted in the target population and the sequence of CD8+ or CD4+ T-cell epitopes associated with each cluster.

| **Cluster** | | | **T-cell epitopes** | |
| --- | --- | --- | --- | --- |
| **Sequence** | **Amino acid position (start-end)** | **Population coverage (%)** | **CD8+ T-cell epitopes** | **CD4+ T-cell epitopes** |
| ST**QDLFLPFFSNVTWF**HA | 52-65 (50-67) | 0.715011 | STQDLFLPF,TQDLFLPFF, LPFFSNVTW,FSNVTWFHA | - |
| GTKRFDN**PVLPFNDGV**YFASTEK | 82-90 (75-97) | 0.999593 | GTKRFDNPV,RFDNPVLPF, LPFNDGVYF,GVYFASTEK | - |
| EFQ**FCNDPFLGVYYHK**NNK | 135-147 (132-150) | 0.696428 | EFQFCNDPF,FQFCNDPFL, FCNDPFLGV,GVYYHKNNK | - |
| KSWME**SEFRVYSSANNCTFEYVSQP**FL | 155-175 (150-176) | 0.66784 | KSWMESEFR,SEFRVYSSA, YSSANNCTF,SANNCTFEY, TFEYVSQPF,FEYVSQPFL | - |
| KIY**SKHTPINLVRDLP**QGF | 205-217 (202-220) | 0.848833 | KIYSKHTPI,YSKHTPINL, TPINLVRDL,LVRDLPQGF | - |
| NENGT**ITDAVDCALDPLSETKCTL**KSFTVEK | 285-303 (280-310) | 0.93656 | NENGTITDA,ITDAVDCAL, AVDCALDPL,ALDPLSETK, ETKCTLKSF,TLKSFTVEK | - |
| NYLYRLF**RKSNLKPFE**RDISTEI | 457-465 (450-472) | 0.905872 | NYLYRLFRK,RLFRKSNLK, KSNLKPFER,FERDISTEI | - |
| HAD**QLTPTWRVYSTGS**NVF | 628-640 (625-643) | 0.394533 | HADQLTPTW,DQLTPTWRV,RVYSTGSNV,VYSTGSNVF | - |
| SVAYSNN**SIAIPTNFT**ISVTTEI | 711-719 (704-726) | 0.886912 | SVAYSNNSI,AYSNNSIAI,IPTNFTISV,FTISVTTEI | - |
| RSFIE**DLLFNKVTLAD**AGFIK | 820-830 (815-835) | 0.387947 | RSFIEDLLF,IEDLLFNKV,KVTLADAGF,TLADAGFIK | - |
| YEQYIKW**PWYIWLGFI**AGLIAIV | 1213-1221 (1206-1228) | 0.707735 | YEQYIKWPW,EQYIKWPWY,WPWYIWLGF,FIAGLIAIV | - |
| FLPFFS**NVTWFHAIH**VSGTNGT | 61-70 (55-76) | 0.401981 | - | FLPFFSNVT,FFSNVTWFH, VTWFHAIHV,IHVSGTNGT |
| YHKNNKS**WMESEFRVYSSANNCTFEYVSQPFLMDLEGKQ**GN | 152-183 (145-185) | 0.758712 | - | YHKNNKSWM,MESEFRVYS,FRVYSSANN,VYSSANNCT, YSSANNCTF,FEYVSQPFL, FLMDLEGKQ,LMDLEGKQG,MDLEGKQGN |
| VTLADAG**FIKQYGDCL**GDIAARD | 833-841 (826-848) | 0.52966 | - | VTLADAGFI,FIKQYGDCL, YGDCLGDIA,CLGDIAARD |
| GLTVLP**PLLTDEMIAQ**YTSALL | 863-872 (857-878) | 0.306009 | - | GLTVLPPLL,LTVLPPLLT, LLTDEMIAQ,IAQYTSALL |
| LNTLVKQ**LSSNFGAISSVLNDILSRLD**KVEA | 966-985 (959-989) | 0.662081 | - | LNTLVKQLS,VKQLSSNFG, FGAISSVLN,ISSVLNDIL, LNDILSRLD,LSRLDKVEA |
| I**ITTDNTFVSGNCDVVI**GIVNNTV | 1115-1130 (1114-1137) | 0.896955 | - | IITTDNTFV,ITTDNTFVS, FVSGNCDVV,VSGNCDVVI, VIGIVNNTV |
| YFKNHTS**PDVDLGDIS**GINASVV | 1162-1170 (1155-1177) | 0.608279 | - | YFKNHTSPD,FKNHTSPDV, LGDISGINA,ISGINASVV |

**Table S15**. Putative T-cell epitope clusters predicted by Predivac-3.0 in the spike glycoprotein of SARS-CoV-2 when targeting the population of Brazil. In each cluster, the core region of overlapped T-cell epitopes is highlighted in bold/underscored, reporting the amino acid position in the protein, the population coverage predicted in the target population and the sequence of CD8+ or CD4+ T-cell epitopes associated with each cluster.

| **Cluster** | | | **T-cell epitopes** | |
| --- | --- | --- | --- | --- |
| **Sequence** | **Amino acid position (start-end)** | **Population coverage (%)** | **CD8+ T-cell epitopes** | **CD4+ T-cell epitopes** |
| GTKRFDN**PVLPFNDGV**YFASTEK | 82-90 (75-97) | 0.9985 | GTKRFDNPV,RFDNPVLPF, VLPFNDGVY,LPFNDGVYF, GVYFASTEK | - |
| KSWME**SEFRVYSSANNCTFEY**VSQPFL | 155-170 (150-176) | 0.7284 | KSWMESEFR, SWMESEFRV,  SEFRVYSSA,YSSANNCTF, SANNCTFEY,TFEYVSQPF, FEYVSQPFL | - |
| AVDCALD**PLSETKCTL**KSFTVEK | 295-303 (288-310) | 0.5059 | AVDCALDPL,ALDPLSETK, ETKCTLKSF,KCTLKSFTV, TLKSFTVEK | - |
| ETKCTLK**SFTVEKGIY**QTSNFRV | 305-313 (298-320) | 0.3229 | ETKCTLKSF,KCTLKSFTV, TLKSFTVEK,GIYQTSNFR, IYQTSNFRV | - |
| FL**PFFSNVTWFHAIHVSG**TNGT | 57-73 (55-76) | 0.6588 | - | FLPFFSNVT,FFSNVTWFH, VTWFHAIHV,WFHAIHVSG, IHVSGTNGT |
| VYYHKNN**KSWMESEFRVYSSANNCTFEYVSQPFLMDLEGKQGNFKNLR**EFVF | 150-190 (143-194) | 0.8421 | - | VYYHKNNKS,YHKNNKSW,MESEFRVYS,FRVYSSANN, VYSSANNCT,YSSANNCTF, FEYVSQPFL,FLMDLEGKQ, LMDLEGKQG,MDLEGKQGN,FKNLREFVF |
| VGYLQ**PRTFLLKYNEN**GTITD | 272-282 (267-287) | 0.4789 | - | VGYLQPRTF,FLLKYNENG, LKYNENGTI,YNENGTITD |
| IVRFP**NITNLCPFGEV**FNATR | 331-341 (326-346) | 0.2582 | - | IVRFPNITN,FPNITNLCP, ITNLCPFGE,FGEVFNATR |
| YNYKLP**DDFTGCVIAWNSNN**LD | 427-440 (421-442) | 0.3067 | - | YNYKLPDDF,LPDDFTGCV, FTGCVIAWN,VIAWNSNNL, IAWNSNNLD |
| YNYLY**RLFRKSNLKPFERD**ISTEI | 454-467 (449-472) | 0.8428 | - | YNYLYRLFR,LYRLFRKSN, YRLFRKSNL,LKPFERDIS, FERDISTEI |
| FNCY**FPLQSYGFQPTN**GVGY | 490-501 (486-505) | 0.3476 | - | FNCYFPLQS,YFPLQSYGF, YGFQPTNGV,FQPTNGVGY |
| VLTESNK**KFLPFQQFG**RDIADTT | 558-566 (551-573) | 0.8752 | - | VLTESNKKF,LTESNKKFL, FQQFGRDIA,FGRDIADTT |
| YGSF**CTQLNRALTGIAVEQ**DKNTQE | 760-774 (756-780) | 0.6102 | - | YGSFCTQLN,FCTQLNRAL, LNRALTGIA,LTGIAVEQD, VEQDKNTQE |
| VTLADAG**FIKQYGDCL**GDIAARD | 833-841 (826-848) | 0.6594 | - | VTLADAGFI,FIKQYGDCL, YGDCLGDIA,CLGDIAARD |
| LNTLVKQ**LSSNFGAISSVLNDILSRLDKVE**AEVQIDR | 966-988 (959-995) | 0.52 | - | LNTLVKQLS,VKQLSSNFG, FGAISSVLN,ISSVLNDIL, VLNDILSRL,LNDILSRLD, LSRLDKVEA,VEAEVQIDR |
| I**ITTDNTFVSGNCDVVI**GIVNNTV | 1115-1130 (1114-1137) | 0.6991 | - | IITTDNTFV,ITTDNTFVS, FVSGNCDVV,VSGNCDVVI, VIGIVNNTV |
| YFKNHTS**PDVDLGDIS**GINASVV | 1162-1170 (1155-1177) | 0.3627 | - | YFKNHTSPD,FKNHTSPDV, LGDISGINA,ISGINASVV |

**Table S16**. Putative T-cell epitope clusters predicted by Predivac-3.0 in the spike glycoprotein of SARS-CoV-2 when targeting the population of South Africa. In each cluster, the core region of overlapped T-cell epitopes is highlighted in bold/underscored, reporting the amino acid position in the protein, the population coverage predicted in the target population and the sequence of CD8+ or CD4+ T-cell epitopes associated with each cluster.

| **Cluster** | | | **T-cell epitopes** | |
| --- | --- | --- | --- | --- |
| **Sequence** | **Amino acid position (start-end)** | **Population coverage (%)** | **CD8+ T-cell epitopes** | **CD4+ T-cell epitopes** |
| GTKRFDN**PVLPFNDGV**YFASTEK | 82-90 (75-97) | 0.998776 | GTKRFDNPV,RFDNPVLPF, VLPFNDGVY,LPFNDGVYF, GVYFASTEK | - |
| KSWME**SEFRVYSSANNCTFEY**VSQPFL | 155-170 (150-176) | 0.690381 | KSWMESEFR,SWMESEFRV,SEFRVYSSA,YSSANNCTF, SANNCTFEY,TFEYVSQPF, FEYVSQPFL | - |
| AVDCALD**PLSETKCTL**KSFTVEK | 295-303 (288-310) | 0.61776 | AVDCALDPL,ALDPLSETK, ETKCTLKSF,KCTLKSFTV, TLKSFTVEK | - |
| ETKCTLK**SFTVEKGIY**QTSNFRV | 305-313 (298-320) | 0.348166 | ETKCTLKSF,KCTLKSFTV, TLKSFTVEK,GIYQTSNFR,  IYQTSNFRV | - |
| F**EYVSQPFLMDLEGKQ**G | 169-183 (168-184) | 0.533511 | - | FEYVSQPFL,FLMDLEGKQ, LMDLEGKQG |
| FGEVFN**ATRFASVYAW**NRKRIS | 344-353 (338-359) | 0.434496 | - | FGEVFNATR,VYAWNRKRI, YAWNRKRIS, |
| LNTL**VKQLSSNFGAI**  **S**SVLN | 963-974 (959-978) | 0.246576 | - | LNTLVKQLS,VKQLSSNFG, FGAISSVLN, |

**Table S17**. Putative T-cell epitope clusters predicted by Predivac-3.0 in the spike glycoprotein of SARS-CoV-2 when targeting the population of Japan. In each cluster, the core region of overlapped T-cell epitopes is highlighted in bold/underscored, reporting the amino acid position in the protein, the population coverage predicted in the target population and the sequence of CD8+ or CD4+ T-cell epitopes associated with each cluster.

| **Cluster** | | | **T-cell epitopes** | |
| --- | --- | --- | --- | --- |
| **Sequence** | **Amino acid position (start-end)** | **Population coverage (%)** | **CD8+ T-cell epitopes** | **CD4+ T-cell epitopes** |
| ST**QDLFLPFFSNVTWF**HA | 52-65 (50-67) | 0.868279 | STQDLFLPF,TQDLFLPFF,  LPFFSNVTW,FSNVTWFHA, | - |
| GTKRFDN**PVLPFNDGV**YFASTEK | 82-90 (75-97) | 0.989373 | GTKRFDNPV,RFDNPVLPF,  LPFNDGVYF,GVYFASTEK, | - |
| EFQ**FCNDPFLGVYYHK**NNK | 135-147 (132-150) | 0.93527 | EFQFCNDPF,FQFCNDPFL,  FCNDPFLGV,GVYYHKNNK, | - |
| KSWME**SEFRVYSSANNCTFEYVSQPF**L | 155-175 (150-176) | 0.933248 | KSWMESEFR,SEFRVYSSA,  YSSANNCTF,SANNCTFEY,  TFEYVSQPF,FEYVSQPFL, | - |
| WTAGAA**AYYVGYLQPR**TFLLKY | 264-273 (258-279) | 0.75569 | WTAGAAAYY,YYVGYLQPR,  YLQPRTFLL,QPRTFLLKY, | - |
| NENGT**ITDAVDCALDPLSET**KCTLKSF | 285-300 (280-306) | 0.955502 | NENGTITDA,ITDAVDCAL,  AVDCALDPL,ALDPLSETK,ETKCTLKSF, | - |
| NYLYRLF**RKSNLKPFE**RDISTEI | 457-465 (450-472) | 0.583106 | NYLYRLFRK,RLFRKSNLK,  KSNLKPFER,FERDISTEI, | - |
| HAD**QLTPTWRVYSTGS**NVF | 628-640 (625-643) | 0.709395 | HADQLTPTW,DQLTPTWRV,  RVYSTGSNV,VYSTGSNVF, | - |
| SVAYSNN**SIAIPTNFT**ISVTTEI | 711-719 (704-726) | 0.926436 | SVAYSNNSI,AYSNNSIAI,  IPTNFTISV,FTISVTTEI, | - |
| VVN**QNAQALNTLVKQLSSNFG**A | 957-971 (951-972) | 0.294634 | VVNQNAQAL,AQALNTLVK  ,ALNTLVKQL,LVKQLSSNF,  KQLSSNFGA | - |
| YEQYIKW**PWYIWLGFI**AGLIAIV | 1213-1221 (1206-1228) | 0.855593 | YEQYIKWPW,EQYIKWPWY  ,WPWYIWLGF,FIAGLIAIV | - |
| FLPFFS**NVTWFHAIHV**SGTNGT | 61-70 (55-76) | 0.452731 |  | FLPFFSNVT,FFSNVTWFH,VTWFHAIHV,IHVSGTNGT, |
| VYYHKNN**KSWMESEFRVYSSANNCTFEYVSQPFLMDLEGKQ**GN | 150-183 (143-185) | 0.887804 |  | VYYHKNNKS,YHKNNKSWM,MESEFRVYS,FRVYSSANN,VYSSANNCT,YSSANNCTF,FEYVSQPFL,FLMDLEGKQ,LMDLEGKQG,MDLEGKQGN, |
| VTLADAG**FIKQYGDCL**GDIAARD | 833-841 (826-848) | 0.518463 |  | VTLADAGFI,FIKQYGDCL,YGDCLGDIA,CLGDIAARD, |
| GLTVLP**PLLTDEMIAQ**YTSALL | 863-872 (857-878) | 0.523345 |  | GLTVLPPLL,LTVLPPLLT,LLTDEMIAQ,IAQYTSALL, |
| LNTLVKQ**LSSNFGAISSVLNDILSRLD**KVEA | 966-985 (959-989) | 0.437343 |  | LNTLVKQLS,VKQLSSNFG,FGAISSVLN,ISSVLNDIL,LNDILSRLD,LSRLDKVEA, |
| I**ITTDNTFVSGNCDVVI**GIVNNTV | 1115-1130 (1114-1137) | 0.779749 |  | IITTDNTFV,ITTDNTFVS,FVSGNCDVV,VSGNCDVVI,VIGIVNNTV, |
| YFKNHTS**PDVDLGDIS**GINASVV | 1162-1170 (1155-1177) | 0.4969 |  | YFKNHTSPD,FKNHTSPDV,LGDISGINA,ISGINASVV, |
